# Cu(II)-tyrosinase enzyme catalyst mediated synthesis of mosquito larvicidal active pyrazolidine-3,5-dione derivatives with molecular docking studies and their ichthyotoxicity analysis

**DOI:** 10.1371/journal.pone.0298232

**Published:** 2024-09-19

**Authors:** Velmurugan Loganathan, SurendraKumar Radhakrishnan, Anis Ahamed, Raman Gurusamy, Omar H. Abd-Elkader, Akbar Idhayadhulla

**Affiliations:** 1 Research Department of Chemistry, Nehru Memorial College (Affiliated Bharathidasan University), Puthanampatti, Tamilnadu, India; 2 Department of Botany and Microbiology, College of Science, King Saudi University, Riyadh, Saudi Arabia; 3 Department of Life Science, Yeungnam University, Gyeongsan, Gyeongbuk-do, South Korea; 4 Department of Physics and Astronomy, College of Science, King Saudi University, Riyadh, Saudi Arabia; Universidade Federal do Para, BRAZIL

## Abstract

The objective of this study was to develop pyrazolidine-3,5-dione derivatives with potential as environmentally friendly pesticides for pest control, specifically focusing on their efficacy as larvicidal agents. A novel one-pot synthesis of multicomponent pyrazolidine-3,5-dione derivatives (**1a-m**) was accomplished via the grindstone method using Cu(II)tyrosinase enzyme as a catalyst under mild reaction conditions, yielding 84%–96%. The synthesised derivatives (**1a-m**) were characterized using various spectroscopic methods (mass spectrometry, elemental analysis, FT-IR, and ^1^H and ^13^C NMR). NMR characterisation using DMSO-*d*_6_ as a solvent. The larvicidal and antifeedant activities of the synthesised compounds were screened and *in silico* computational studies were performed. The larvicidal activity against *Culex quinquefasciatus* and antifeedant activity against *Oreochromis mossambicus* were evaluated. Among the synthesised compounds, compound **1c** demonstrated superior efficacy (LD_50_: 9.7 μg/mL) against *C*. *quinquefasciatus* compared to permethrin (LD_50_: 17.1 μg/mL). Regarding antifeedant activity, compounds **1a**, **1e**, **1f**, **1j**, and **1k** exhibited 100% mortality at 100 μg/mL. Molecular docking analysis was performed to assess the binding capacity of a mosquito odorant-binding protein (3OGN) from *Culex quinquefasciatus* to compound **1c**. The results revealed that compound **1c** had a docking score of -10.4 kcal/mol, surpassing that of standard permethrin (-9.5 kcal/mol). Furthermore, DFT calculations were conducted to acquire theoretical data aligned with the experimental FT-IR results. According to experimental research, compound **1c** demonstrates promising larvicidal activity against mosquito larvae of *C*. *quinquefasciatus*.

## Introduction

Millions of people succumb annually to mosquito-borne illnesses like malaria, filariasis, dengue, and yellow fever, rendering mosquitoes the most perilous insects [1]. The most effective preventive measure is controlling mosquito larvae, pivotal as vectors for disease transmission. Regular application of larvicidal chemicals, notably organophosphates and insect development inhibitors, is commonly employed for this purpose [2]. However, the frequent utilization of these chemicals adversely affects unintended populations and may potentially lead to the emergence of resistant strains, raising concerns [3]. Hence, there is an imperative need for safer and more efficient approaches to control mosquito larvae.

Among these vectors, *Culex quinquefasciatus* mosquitoes are the most frequently associated with human habitats in both urban and rural areas [4, 5]. In recent years, research has been directed towards botanical pesticides as a pursuit of natural alternatives to synthetic insecticides. These alternatives are intriguing due to their versatility in roles such as growth inhibitors, insecticides, larvicides, antifeedants, repellents, or oviposition deterrents. They are natural, biodegradable, and exhibit minimal toxicity [6]. Alkaloid isoquinolines have garnered significant interest as genetic precursors to various biologically active compounds, holding promise for applications in disease treatment [7, 8] and insect management [9].

**Fig 1:**
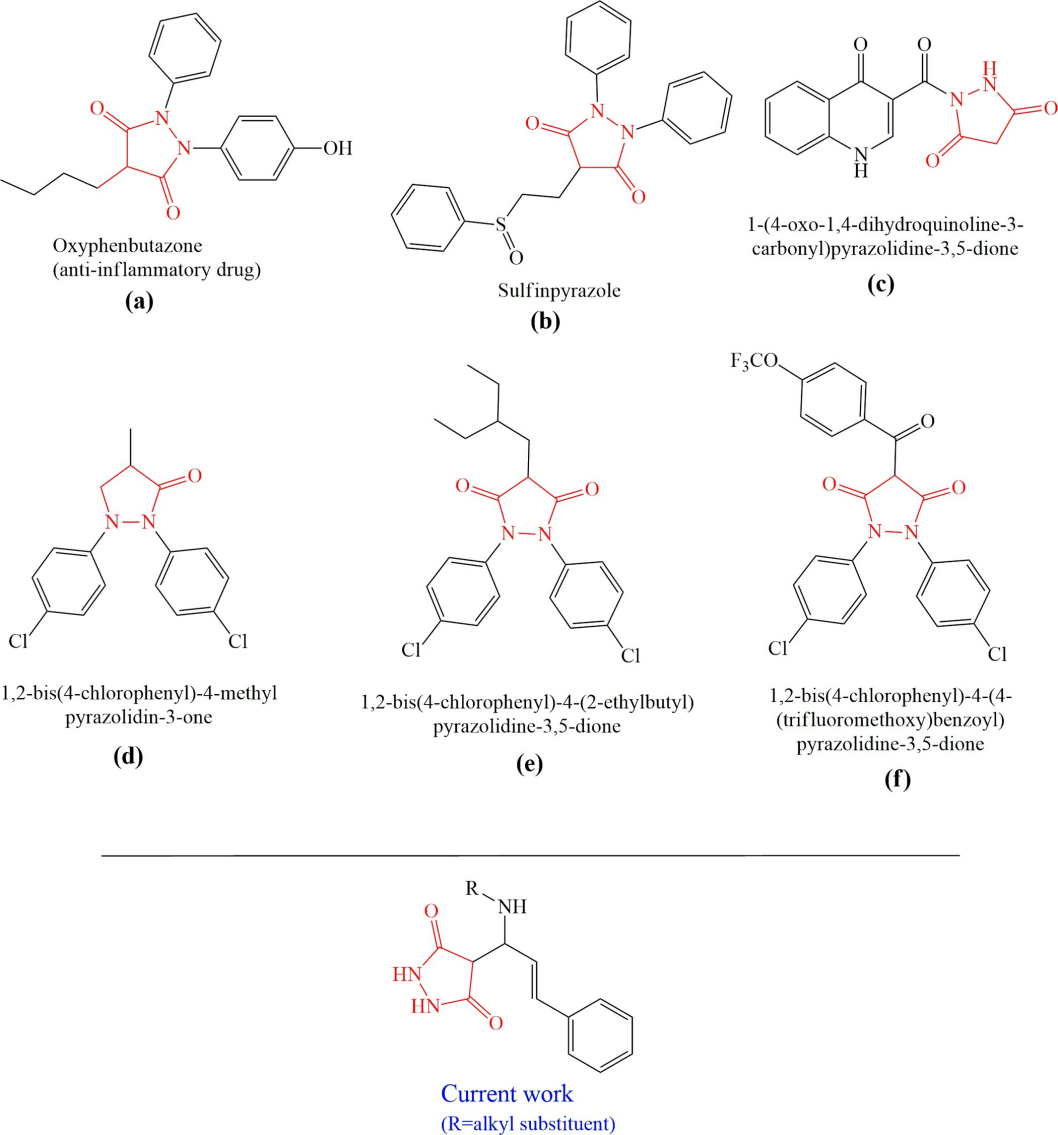
Some bioactive and previously reported pyrazolidine derivative.

Chemists are intrigued by heterocycles and their derivatives due to the diverse biological and pharmacological attributes within this molecular family, particularly those containing nitrogen [10, 11]. The carbonyl (C = O) group in pyrazolidine-3,5-dione derivatives is pivotal for various pharmaceutical and biological activities [12]. Pyrazolidine-3,5-dione derivatives have attracted considerable attention owing to their broad spectrum of biological activities, including antitumor, anti-HIV, anti-inflammatory, COX-2 inhibitory, and significant anti-metastatic effects [13–16]. Fig 1 illustrates some bioactive pyrazolidine derivatives previously reported [11, 17, 18].

The dinuclear copper core of mushroom tyrosinase catalyses hydroxylation and oxidation processes [19–25]. Cu(II)-O_2_^2—^Cu(II) forms a μ-ŋ2:ŋ2 side-on bridging bond with dioxygen, where O_2_^2-^ is in the oxy state. The met type [Cu(II)-Cu(II)] designates a state in which only the Cu atoms at the active site undergo oxidation and are not linked to dioxygen. Tyrosinase functions as a catalyst and contains water molecules or hydroxide ions along with Cu^+2^, connected by one or two small ligands. Mannich-type reactions, while facilitating the formation of compounds, often encounter challenges in terms of reaction conditions, catalyst reaction time, separation, and toxicity of the final product(s) [26]. The aim of developing the Cu(II)-Tyr enzyme as a novel eco-friendly catalyst was to address the challenges of low yields, harsh conditions, and lengthy reaction times. Previously reported literature on Cu(II)tyrosinase catalysts and proteins, specifically focusing on larvicidal and antifeedant activity, was considered [27–29]. Pyrazolidine-3,5-dione derivatives have been reported to exhibit various activities. For the first time, this derivative was synthesised using Cu(II)-Tyr catalyst and screened for larvicidal and antifeedant activities.

In this study, we successfully synthesised novel one-pot multicomponent pyrazolidine-3,5-dione derivatives via the grindstone method using a Cu(II)tyrosinase catalyst. Moreover, the study aimed to investigate the larvicidal and antifeedant activities of all synthesised compounds and computationally evaluated them as potential agents against 3OGN through molecular docking simulations. Based on the findings of this study, the synthesised derivatives demonstrate larvicidal activity and potential as eco-friendly pesticides for pest control.

## Materials and methods

### Chemistry

From Sigma-Aldrich in St. Louis, Missouri, United States, all analytical-grade chemicals were acquired. Melting points were noted in the open capillary tubes. FTIR (4000–400 cm^−1^) was checked from Thermo Scientific Nicolet iS5. NMR spectra (^1^H and ^13^C) were checked with Bruker (DRX-75 and 300 MHz) instrumentation. The elements C, H, S, and N were examined using a Vario EL III elemental analyzer. Mass spectrum was employed using PerkinElmer GCMS model Clarus sq8 (EI).

### Synthesis of 4-(1-hydrazinyl-3-phenylallyl)pyrazolidine-3,5-dione (1a)

A mixture 1.32 mL of cinnamaldehyde (0.01 mol), 1.20 mL of pyrazolidine-3,5-dione (0.01 mol), hydrazine (0.01 mol) and 0.5 g of Cu(II)-tyr enzyme were mixed in a grout and powdered at ambient temperature. To recover the catalyst, pH 6.0 potassium phosphate buffer (2 mL, 50 mM) was added and the mixture was filtered. The progress of the reaction was monitored by TLC. Column chromatography (Ethyl acetate 4: hexane 6) was used to separate the final solid material, the procedure was followed by remaining compounds **1(b-m).** Detailed physical values, spectral, mass, and Analytical values of compounds (**1a-m**) were reported in supporting information (SI) file.

### Biological activities

#### Larvicidal activity

The synthesis of (**1a-m**) was carried out according to a previously reported protocol [30] (More information in SI file).

#### Antifeedant activity

The compounds were examined for antifeedant activity and assessed in aquatic species that were not intended targets. The antifeedant activity was assessed using a previously described method [30] (More information in SI file).

### Molecular docking study

Molecular docking experiments were conducted to evaluate the binding and interaction of compound **1c**, permethrin, and 3OGN protein using AutoDock Vina 1.1.2 software (http://mgltools.scripps.edu) [31]. The detailed experimental given in SI file.

### MD simulation

To assess the stability of the docked complexes found by IFD analysis, a molecular dynamics simulation was performed using Desmond (Schrodinger Biosuite). The MD simulation procedure followed a previously reported method [32, 33].

### DFT calculation

Comparable theoretical data were obtained by DFT calculations using the B3LYP/6–31G (d, p) basis set. These results were congruent with the experimental findings from FT-IR, and the energy gap between the HOMO and LUMO [34]. The detailed experimental given in SI section.

## Results and discussion

### Chemistry

**Scheme 1:**
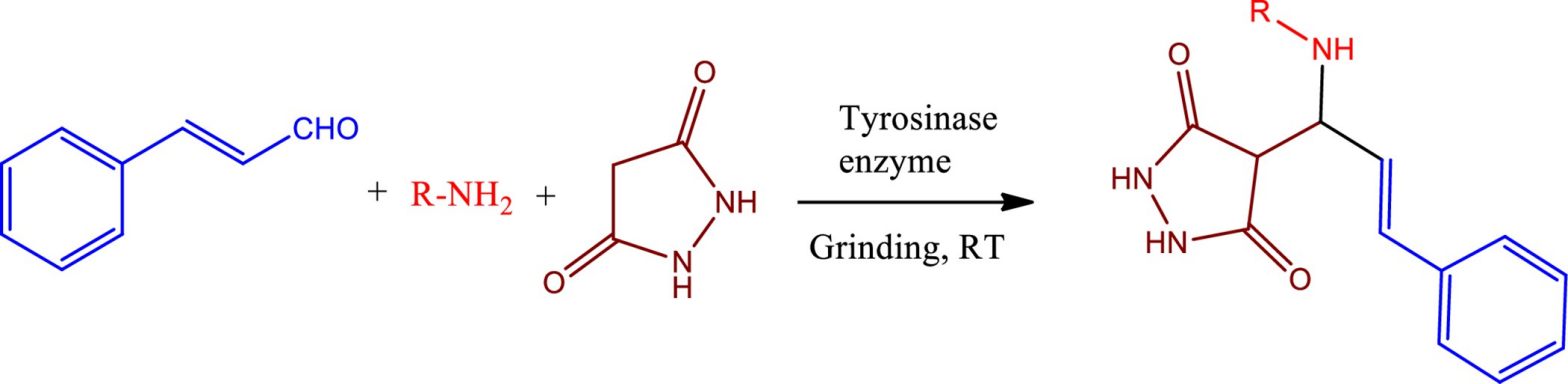
Synthetic pathway for the Mannich base derivative.

In a one-pot multicomponent synthesis, a series of pyrazolidine-3,5-dione derivatives (**1a-m**) were prepared using Cu(II)tyrosinase as the catalyst and the grindstone technique. A mixture of pyrazolidine-3,5-dione, cinnamaldehyde, hydrazine hydrate, and catalytic amounts of the Cu(II)-Tyr enzyme was ground in a mortar and subsequently purified by column chromatography. Scheme 1 depicts the general layout of the synthetic pathway for the Mannich base derivative. The amines and chemicals used to optimize the reaction conditions are detailed in Table 1.

**Table 1:** Optimization of reactants and yield with final product for compounds (1a-m).

Compounds	-R	Final product	Yield (%)
**1a**	NH_2_-NH_2_	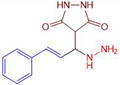	96
**1b**	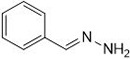	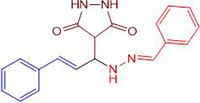	91
**1c**	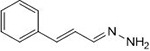	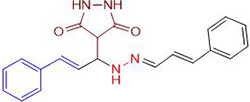	83
**1d**	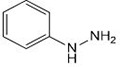	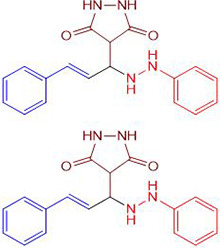	87
**1e**	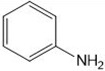	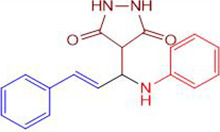	91
**1f**	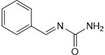	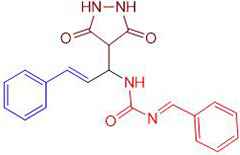	92
**1g**	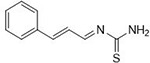	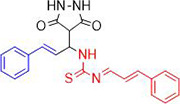 >	88
**1h**	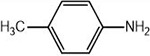	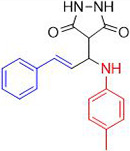	84
**1i**	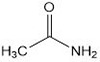	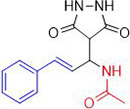	86
**1j**	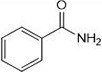	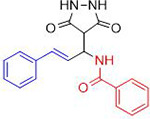	83
**1k**	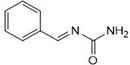	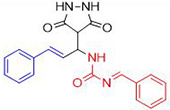	92
**1l**	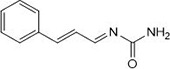	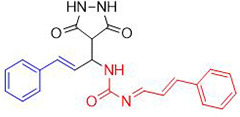	94
**1m**	H_3_C-NH_2_	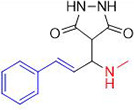	84

Various Cu(II) catalysts were employed to optimize the reaction using both the grindstone method without solvent and the conventional method with CHCl_3_ solvent. The reaction for compound **1a** was conducted at room temperature, and a total of 14 catalysts were employed for its optimization. Cu(II)-catalysed reactions (0.5 equivalent) in the grindstone method at 2 min yielded lower compared to the conventional method. Conversely, no yield was obtained without a catalyst. Three enzyme catalysts were optimized for the reaction, with the tyrosinase from mushroom catalysing well compared to other enzyme catalysts. The tyrosinase-catalysed reaction achieved a 96% yield in the grindstone method without solvent, while the conventional method yielded only 45% after 1 h of reaction time. The catalyst optimization results are summarized in Table 2.

**Table 2:** Catalyst optimization for compound 1a with room temperature.

Entry	Catalyst	Equiv.	Grindstone method Without solvent		Conversional method With CHCl_3_	
			Time (min)	Yield (%)	Time (min)	Yield (%)
1	No catalysis		2	-	30	-
2	CuCl_2_.2H_2_O	0.5	2	12	30	22
3	Copper(II) oxide	0.5	2	14	30	25
4	CuSO_4_	0.5	2	15	30	22
5	Cu(OTf)_2_	0.5	2	10	30	23
6	Dichloro(1,10-phenanthroline) copper(II)	0.5	2	-	30	-
7	Copper(II) *tert*-butylacetoacetate	0.5	2	-	30	-
8	Copper(II) acetate	0.5	2	6	3	12
9	Copper(II) acetylacetonate	0.5	2	-	30	10
10	Ammonium tetrachlorocuprate(II) dehydrate (NH_4_)_2_CuCl_4 _· 2H_2_O	0.5	2	-	30	-
11	copper(II)ethyl acetoacetate	0.5	2	5	30	18
12	Trypsin from bovine pancreas	0.5	2	55	30	22
13	Lipase from Candida antarctica	0.5	2	61	30	26
14	Cu(II)-Tyrosinase from mushroom	**0.5**	**2**	**92**	**30**	**36**
		**1**	**5**	**96**	**1hr**	**45**

However, the Cu(II)acetylacetonate catalyst in various solvents (toluene, CHCl_3_, n-hexane, CH_2_Cl_2_, THF, and dichloroethane) at room temperature yielded product **1a** in varying percentages, namely 32%, 36%, 22%, 13%, 15%, and 10%, respectively. When the reaction was conducted in CHCl_3_ under reflux, a significantly higher yield of 57% was achieved (Table 3, entry 6). Conversely, the presence of catalysts, including CuCl_2_, at room temperature for a maximum of 2 minutes resulted in a lower yield of 12% (Table 2, entry 2). The Cu(II) tyrosinase catalyst (0.5 equivalent) at room temperature provided a high yield of 92%, and using 1 equivalent of this catalyst yielded an even higher percentage of 96% (entry 14, Table 2), surpassing yields obtained with much higher proportions of other catalysts (0.5 equiv.).

**Table 3:** Optimization of solvent using conversion method synthesis of compound 1a.

Entry	Solvent	Condition Reflux, rt	Yield [%]
1	No solvent	30min	-
		1hr	-
		2hr	32
1	Acetonitrile	30min	10
		1hr	16
		2hr	19
2	Methanol	30min	20
		1hr	23
		2hr	35
3	Ethanol	30min	17
		1hr	23
		2hr	31
4	Benzene	30min	9
		1hr	15
		2hr	21
5	Toluene	30min	32
		1hr	40
		2hr	48
6	**CHCl**_**3**_	**30min**	**36**
		**1hr**	**45**
		**2hr**	**57**
7	n-hexane	30min	22
		1hr	36
		2hr	40
8	CH_2_Cl_2_	30min	13
		1hr	24
		2hr	32
9	THF	30min	15
		1hr	26
		2hr	36
10	Dichloroethane	30min	10
		1hr	15
		2hr	20

**Scheme 2:**
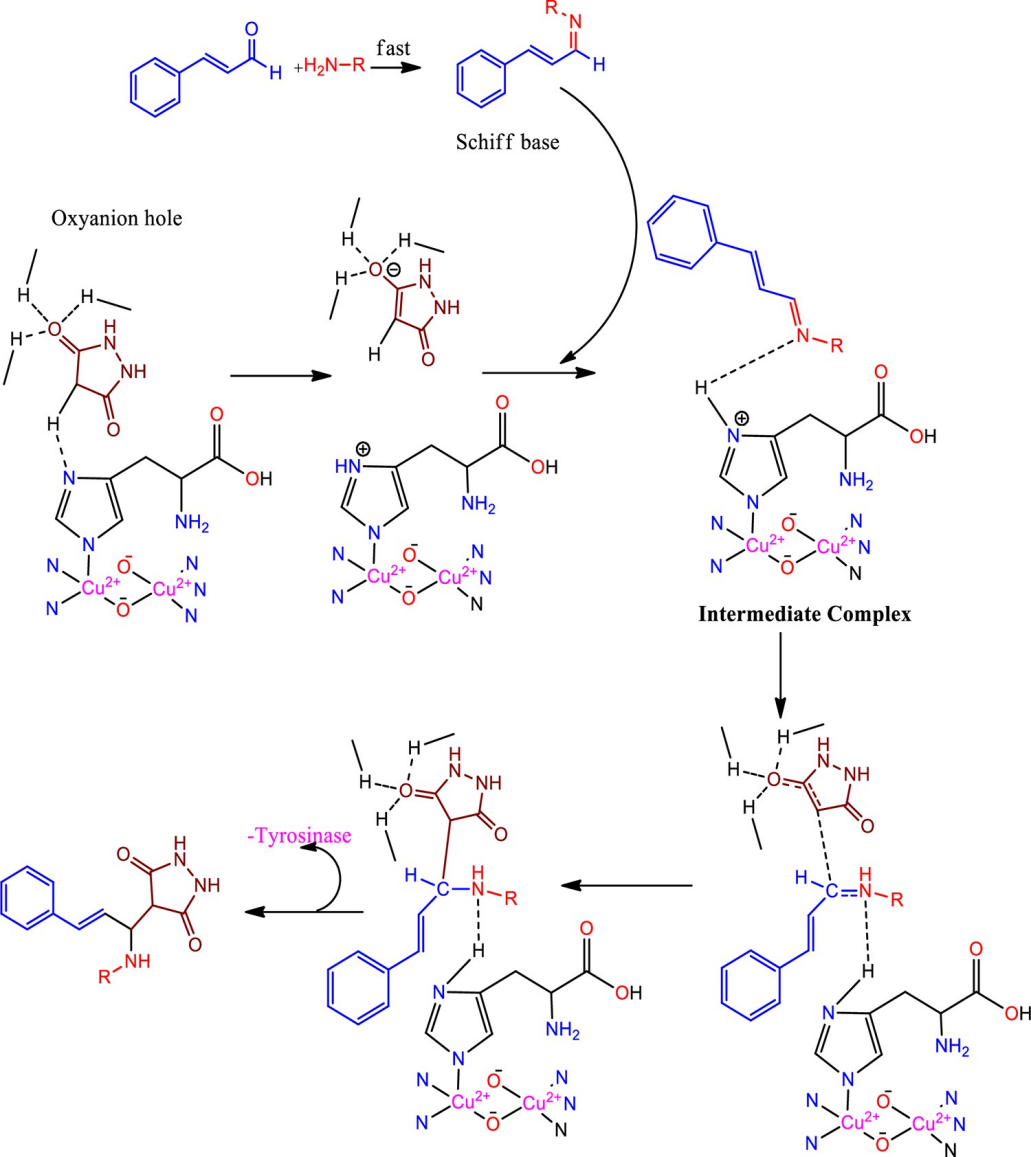
Mechanism of the synthesis of compound 1a.

In Scheme 2, we propose a model of the Cu(II)-Tyr-catalysed Mannich reaction. The Schiff base is initially formed by the reaction of an aldehyde and an amine, and pyrazolidine-3,5-dione is preactivated by Cu(II)-Tyr, yielding the enolate anion. Subsequently, the Schiff base collaborates with the Cu(II)-Tyr-His molecule to create an intermediate complex.

Mannich base reactions relying on Cu-containing substances, such as Cu(CF_3_SO_4_)_2_, Cu(CH_3_COO)_2_, CuBr, and Cu NPs, as well as enzymes like trypsin, lipase, and protease, have been employed to catalyse the one-pot multicomponent Mannich process. This study focused on the synthesis of *N*-Mannich base derivatives (**1a-m**), which were catalysed by a copper-containing enzyme called Cu(II)-Tyr.

The catalysts were optimized using trypsin, lipase, CuCl_2_.2H_2_O, and Cu(II)-Tyr, resulting in yields of 55%, 61%, 12%, and 92%, respectively. Table 2 outlines the optimized reaction conditions for the catalysts, which were employed to assess the reaction yield. No reaction occurred without solvent, even after 2 h of reflux at room temperature using the conventional method. Acetonitrile as the solvent under reflux produced a 19% yield after 2 h, while methanol and ethanol yielded 35% and 31% at 2 h, respectively (Table 3, entries 2,3). In contrast, using benzene as the medium enhanced the yield to 21% (Table 3, entry 4). However, all solvents tested were less active than CHCl_3_, which exhibited high activity after 2 h. The optimization of the solvent and its performance is summarized in Table 3.

NMR (^1^H and ^13^C) and FT-IR spectroscopies were employed to evaluate the synthesised compounds. In the IR spectra, the compounds exhibited significant bands indicative of–NH–,–CO, and–C = C–groups at 3495–3571, 1700–1767, and 1610–1680 cm^-1^, respectively. The ^1^H NMR signals were observed at 8.03–2.0, 7.40–7.24, and 6.56 ppm, corresponding to -NH, Ph-ring, and Ph-CH protons. The ^13^C NMR displayed peaks at 207.1–164.5, 136.4–127.9, and 56.2–45.4 ppm, corresponding to -CO, -Ph-ring, and -NH-CH- atoms, respectively. The conformation of each synthesised compound was confirmed through mass spectrometry and elemental analysis. Detailed Mass, FTIR, and NMR (^1^H and ^13^C) spectra are provided in the SI (S1-S52 Figs in S1 File).

“Imine precursors are often used to organise *E*-alkenyl imines from equivalent *E*-alkenyl aldehydes. The process involves the formation of carbon-carbon bonds, which enables the conversion of in situ generated *E*-alkenyl imine from E-alkenyl aldehydes and secondary amines, as well as acetophenone, in the presence of 5 mol% Cu(II)-tyrosinase catalyst. This results in the production of Mannich adducts (**1a-m**) with moderate to good yields and high *E*-selectivity”. The stereochemistry of the *E* isomers in compound **1a** was unequivocally established by NOE NMR data (see S1 File), which is supported by the following evidence. Consequently, the results of this study show that the downfield shift in the spectroscopic characteristics is more pronounced for the *E*-isomer of pyrazolidine-3,5-dione than for the *Z*-isomer [35].

### Catalyst recovery studies

**Fig 2:**
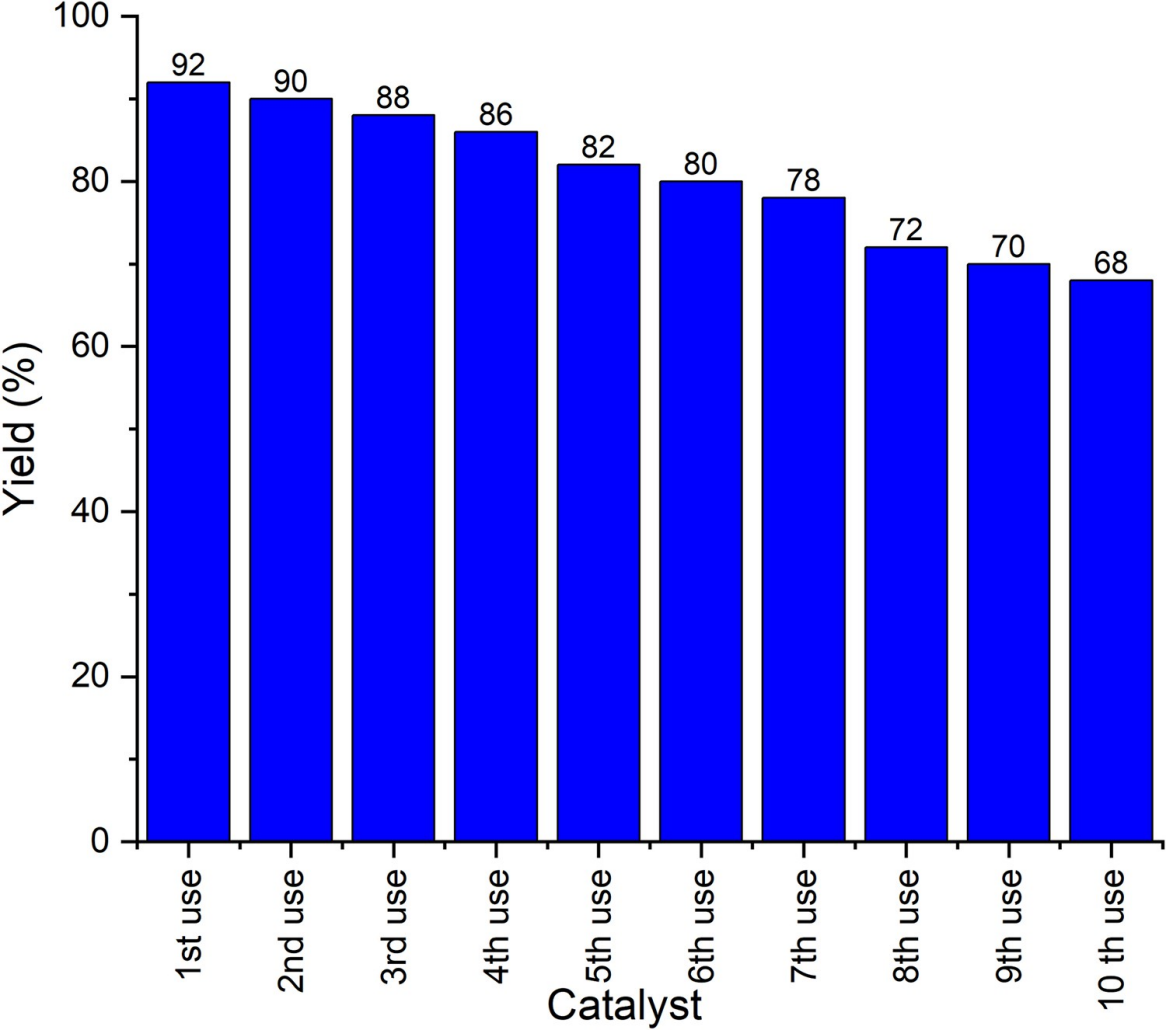
Recovery of catalyst.

At least 10 recycling runs were performed to test the catalytic efficiency. The recyclability of the catalyst was analysed using compound **1a** in the Cu(II)-Tyr enzyme catalyst (Fig 2). The 1^st^ use of the catalyst reached 92%, whereas the 2^nd^ and up to 10^th^ cycles of the reaction were readily used in low yields compared to the 1^st^ cycle of the reaction.

### Biological activity

#### Larvicidal activity

The effectiveness of the synthesised compounds **1(a-m)** was assessed in *C*. *quinquefasciatus* (second-instar larvae). Compound **1c** caused 100% mortality at 100 μg/mL, whereas compounds **1a**, **1f**, **1g**, and **1m** caused 80% mortality at the same concentration. Compound **1c** was highly active (LD_50_ value of 9.7 μg/mL) compared with permethrin (LD_50_ value of 17.1 μg/mL) and other compounds. Compounds **1a**, **1b**, and **1(d-m)** showed low activity against compound **1c** and permethrin. Because of their chemical performance, compounds **1f** and **1g** were equipotential active (LD_50_ values of 58.7 and 59.4 μg/mL); yet, the biological activity was manifested in distinct ways by a few significant functional groups. Table 4 shows that Larvicidal activity of compounds **1(a-m**).

**Table 4:** Larvicidal activity of pyrazolidine-3,5-dione derivatives (1a-m).

Compounds	% of Mortality at concentration (μg/mL)			LD_50_ (μg/mL)^a^
	25	50	100	
**1a**	25.3 ± 0.4	46.9 ± 0.1	82.2 ± 0.6	56.4
**1b**	15.6 ± 0.7	29.3 ± 0.5	45.1 ± 0.2	>100
**1c**	53.8 ± 0.1	81.1 ± 0.1	100 ± 0.0	9.7
**1d**	21.3 ± 0.1	34.3 ± 0.2	45.4 ± 0.2	>100
**1e**	33.3 ± 0.1	48.3 ± 0.2	60.4 ± 0.2	66.1
**1f**	25.0 ± 0.2	44.1 ± 0.2	80.0 ± 0.2	58.7
**1g**	24.1 ± 0.2	43.2 ± 0.1	80.2 ± 0.2	59.4
**1h**	11.2 ± 0.2	27.1 ± 0.2	40.1 ± 0.1	>100
**1i**	19.3 ± 0.1	26.3 ± 0.1	40.2 ± 0.1	>100
**1j**	10.9 ± 0.1	21.7 ± 0.2	41.5 ± 0.1	>100
**1k**	31.0 ± 0.1	46.1 ± 0.1	59.4 ± 0.2	70.7
**1l**	24.1 ± 0.2	43.1 ± 0.2	78.1 ± 0.1	60.5
**1m**	29.0 ± 0.1	49.1 ± 0.1	83.0 ± 0.2	53.1
**Permethrin**	51.1 ± 1.0	76.3 ± 0.1	100 ± 0.0	17.1

^a^ Values are presented as the mean ± SD (n = 3).

#### Antifeedant activity

The harmfulness of the synthesised compounds was investigated using antifeedant activity (marine fish *O*. *mossambicus*). Many of the synthesised compounds were highly toxic. Compounds **1a**, **1f**, **1g**, **1j**, and **1k** exhibited 100% mortality at 100 μg/mL, whereas compounds **1c** and **1d** were less toxic, with 0% mortality at 100 μg/mL. Compound **1e** was highly active (LD_50_ value of 9.7 μg/mL) compared with other compounds. Compounds **1j** and **1k** showed equipotential activity owing to the presence of different functional groups. Table 5 presents the antifeedant activity results.

**Table 5:** Antifeedant activity of pyrazolidine-3,5-dione derivatives (1a-m).

Compounds	% of Mortality at concentration (μg/mL)				LD_50_ (μg/mL)^a^
	10	25	50	100	
**1a**	32.15 ± 0.21	68.19 ± 0.10	89.20 ± 0.15	100 ± 0.00	12.48
**1b**	20.12 ± 0.13	25.26 ± 0.30	31.08 ± 0.20	35.03 ± 0.83	>100
**1c**	0 ± 0.00	0 ± 0.00	0 ± 0.00	0 ± 0.00	>100
**1d**	0 ± 0.00	0 ± 0.00	0 ± 0.00	0 ± 0.00	>100
**1e**	41.02 ± 0.25	69.2 ± 0.14	83.98 ± 0.14	100 ± 0.00	6.01
**1f**	39.05 ± 0.74	50.13± 0.00	74.08 ± 0.00	100 ± 0.00	22.97
**1g**	-	06.21 ± 0.03	12.91 ± 0.23	23.55 ± 0.01	>100
**1h**	8.12 ± 0.02	15.13 ± 0.12	39.43 ± 0.10	43.09 ± 0.03	>100
**1i**	10.02 ± 0.12	20.26 ± 0.56	38.03 ± 0.01	46.09 ± 0.13	>100
**1j**	42.22± 0.41	59.25 ± 0.35	88.23 ± 0.00	100 ± 0.00	10.35
**1k**	33.12 ± 0.00	67.11 ± 0.74	87.87 ± 0.00	100 ± 0.00	12.84
**1l**	-	05.18 ± 0.13	10.31 ± 0.13	20.18 ± 0.13	>100
**1m**	0 ± 0.00	0 ± 0.00	0 ± 0.00	0 ± 0.00	>100

^a^ Values are presented as the mean ± SD (n = 3).

### Molecular docking

**Fig 3:**
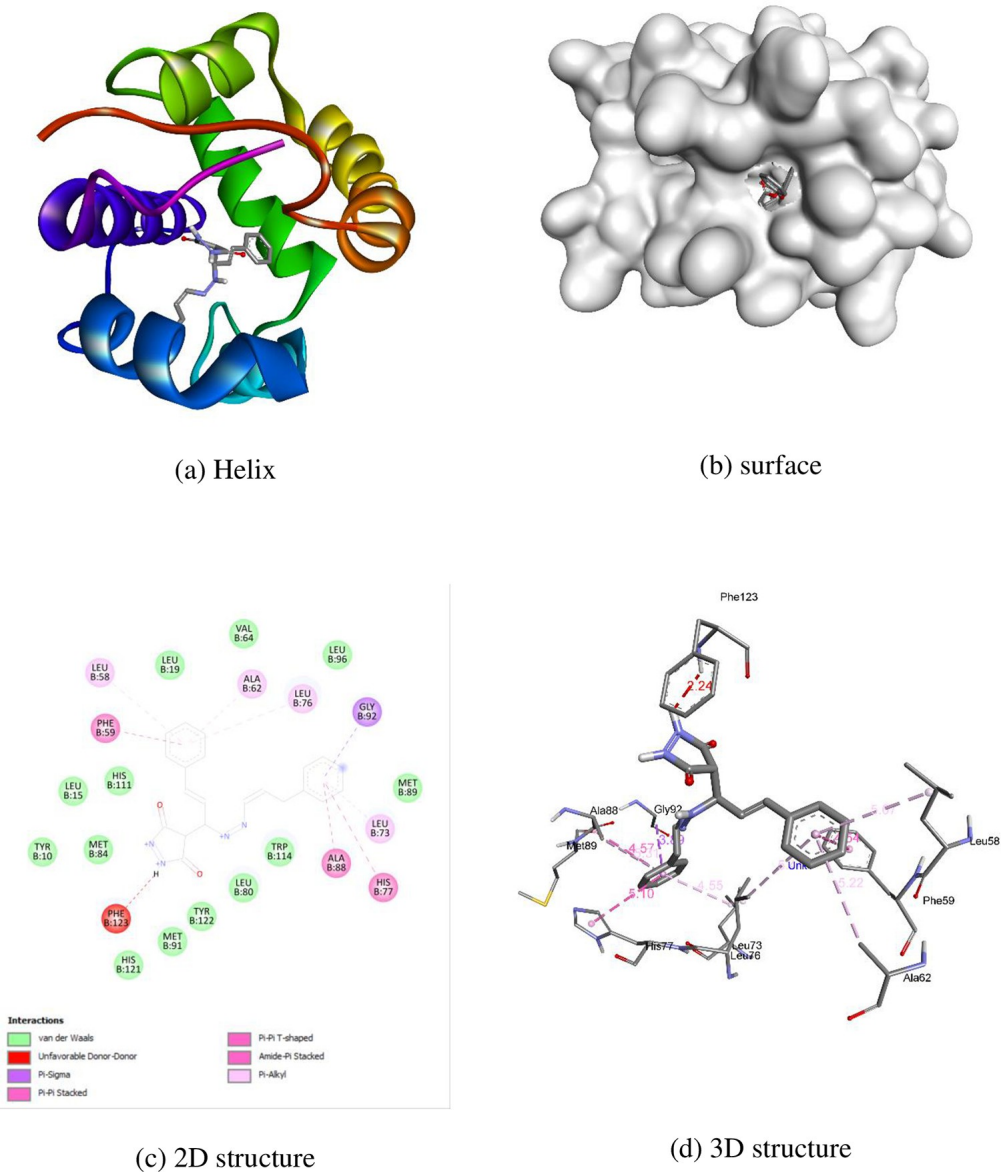
Molecular docking representation of compound 1c with 3OGN protein.

**Fig 4:**
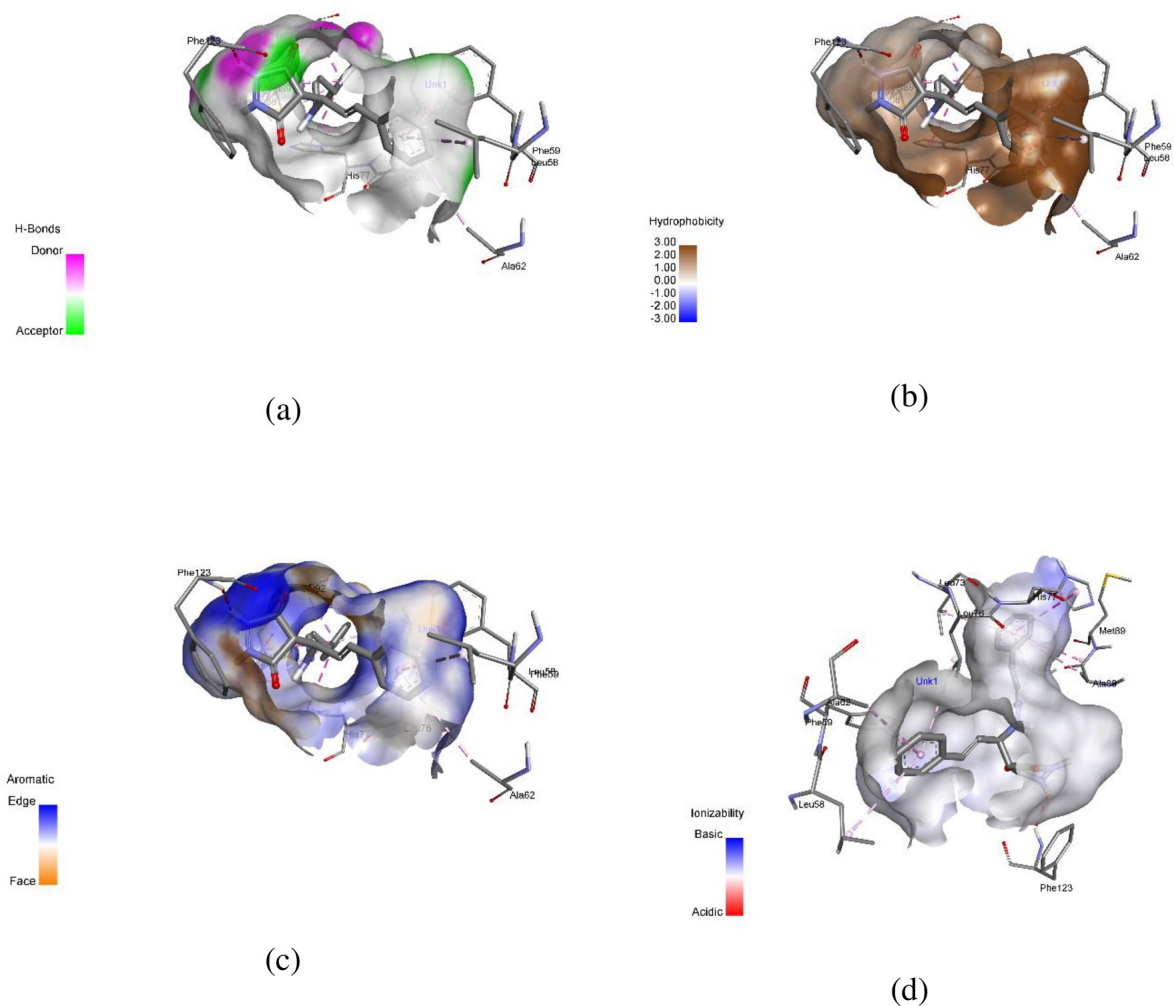
Representation of (a) hydrogen bonding, (b) hydrophobic interactions, (c) aromatic interactions, and (d) ionizability of the complex between 3OGN and compound **1c**.

**Fig 5:**
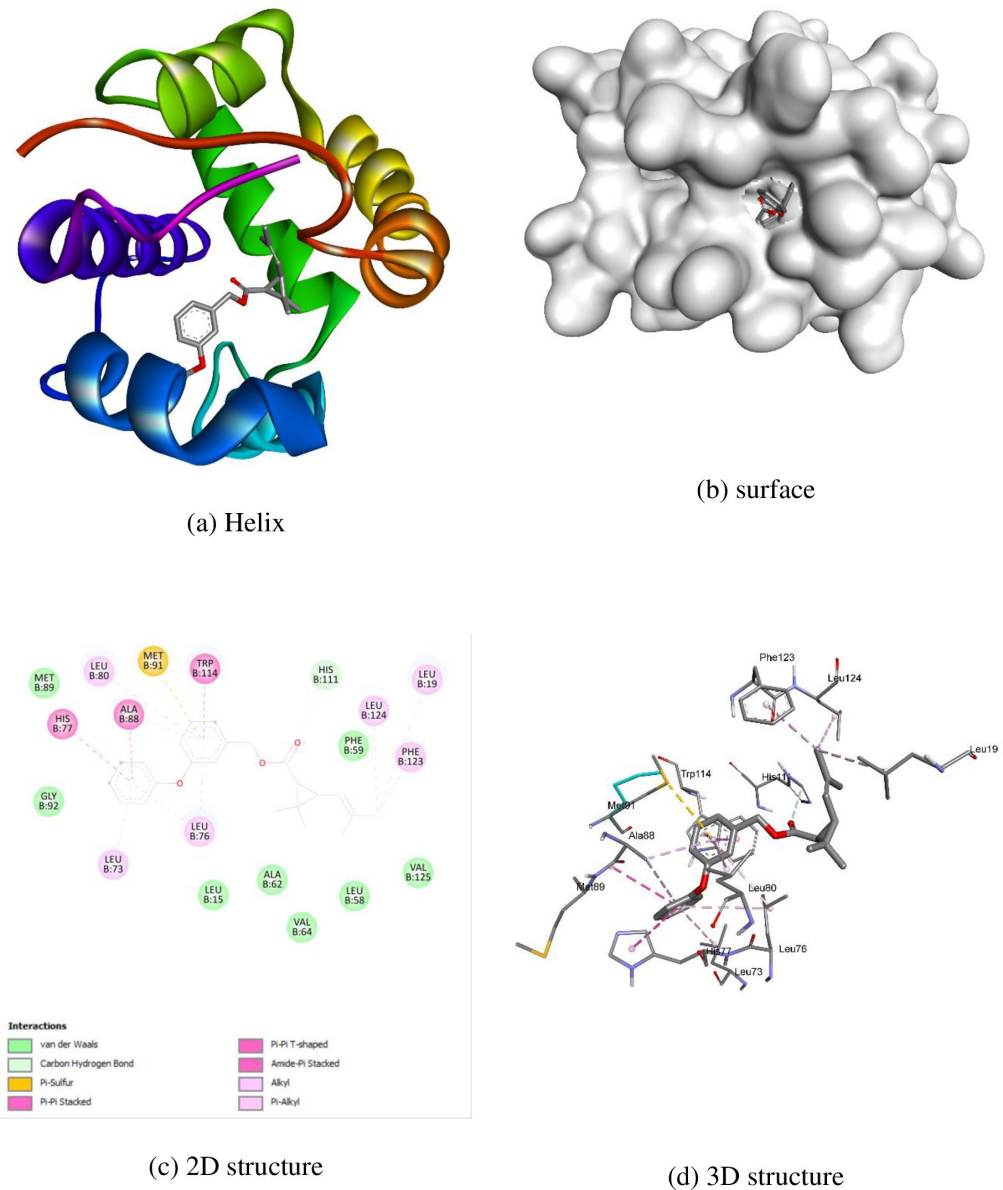
Molecular docking representation of permethrin with 3OGN protein.

**Fig 6:**
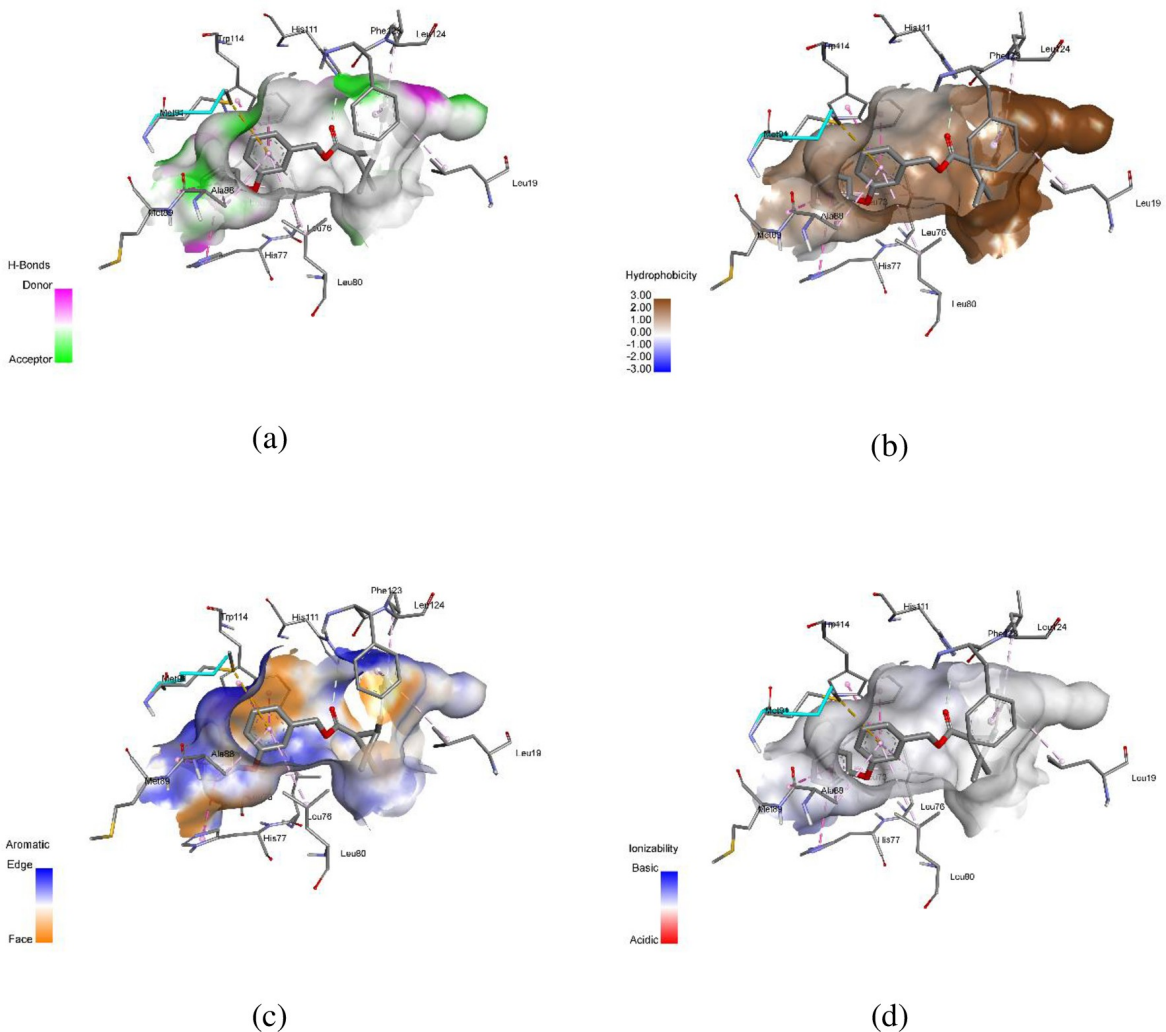
Representation of (a) hydrogen bonding, (b) hydrophobic interactions, (c) aromatic interactions, and (d) ionizability of the complex between 3OGN and permethrin.

AutoDock Vina software was used to conduct molecular docking studies. The highly active compound **1c** was compared with permethrin concerning 3OGN protein. Compound **1c** exhibited a higher binding affinity (-10.4 kcal/mol) compared to standard permethrin (-9.5 kcal/mol). Table 6 presents the results for compound **1c** and standard permethrin with **3OGN**. Neither the standard nor the compound **1c** formed hydrogen bonds. In compound **1c**, residues TYR10, LEU15, LEU19, LEU58, PHE59, ALA62, VAL64, LEU73, LEU76, HIS77, LEU80, MET84, ALA88, MET89, MET91, GLY92, LEU96, HIS111, TRP114, HIS121, TYR122, and PHE123 engaged in hydrophobic connections. The interactions of compound **1c** helix (a), surface (b), 2D structure (c), and 3D structure are shown in Fig 3. (a) Hydrophobic, (b) ionizable, (c) aromatic, and (d) hydrogen bond surfaces at the interaction sites of **1c** and 3OGN are depicted in Fig 4. Amino acid residues LEU15, LEU19, LEU58, PHE59, ALA62, VAL64, LEU73, LEU76, HIS77, LEU80, ALA88, MET89, MET91, GLY92, HIS111, TRP114, PHE123, and LEU124 are involved in hydrophobic interactions with each other in permethrin. The permethrin helix (a), surface (b), 2D structure (c), and 3D structural interactions are presented in Fig 5. (a) Hydrophobic, (b) ionizable, (c) aromatic, and (d) hydrogen bond surfaces at the interaction sites of permethrin and 3OGN are depicted in Fig 6 [36, 37].

**Table 6:** Docking result of compound 1c and standard permethrin with 3OGN.

S. No	Compound/Drug	Dock Score	Interacting residues	Bond Length
**1.**	**1c**	-10.4	Phe 123	-
**2.**	**Permethrin**	-9.5	-	-

### MD simulations

The stability of ligand 1c inside its docked complex with 3OGN was examined by molecular dynamics simulations using both Desmond and Schrödinger tools. The PRODRG server was employed to generate the ligand topology, which was subsequently combined with the protein topology using the GROMOS 43a1 force field and a solvation method utilising a single-point charge water model. The system was constructed with a cubic box that extended 2 nm from the protein surface. To ensure neutrality, the necessary ions were added, and the docked complex energy was minimised using the steepest descent algorithm.

The PME method was used to compute the electrostatics and bond lengths, which were subsequently constrained using the LINCS algorithm. The NVT and NPT ensembles were utilised to reach equilibrium in the systems for each 100 ps simulation, employing a V-rescale thermostat with a reference temperature of 300 K. For every 100 ps simulation, the NVT and NPT ensembles were used to bring the systems to equilibrium using a V-rescale thermostat set to 300 K as a reference. The coordinates of the docked complex structure were stored every 10 picoseconds (ps) for further study throughout the 10-nanosecond (ns) production MD simulation, which used a 2-femtosecond (fs) time step. The analysis of the results was carried out using RMSD, RMSF, gyration, and hydrogen bond plots, and the graphs were plotted using Xmgrace software [38]. In previous studies, we compared molecular dynamics simulations to examine the interactions and stability of compounds with native ligands (permethrin) and their native ligands [39].

### Root Mean Square Deviation (RMSD) analysis

**Fig 7:**
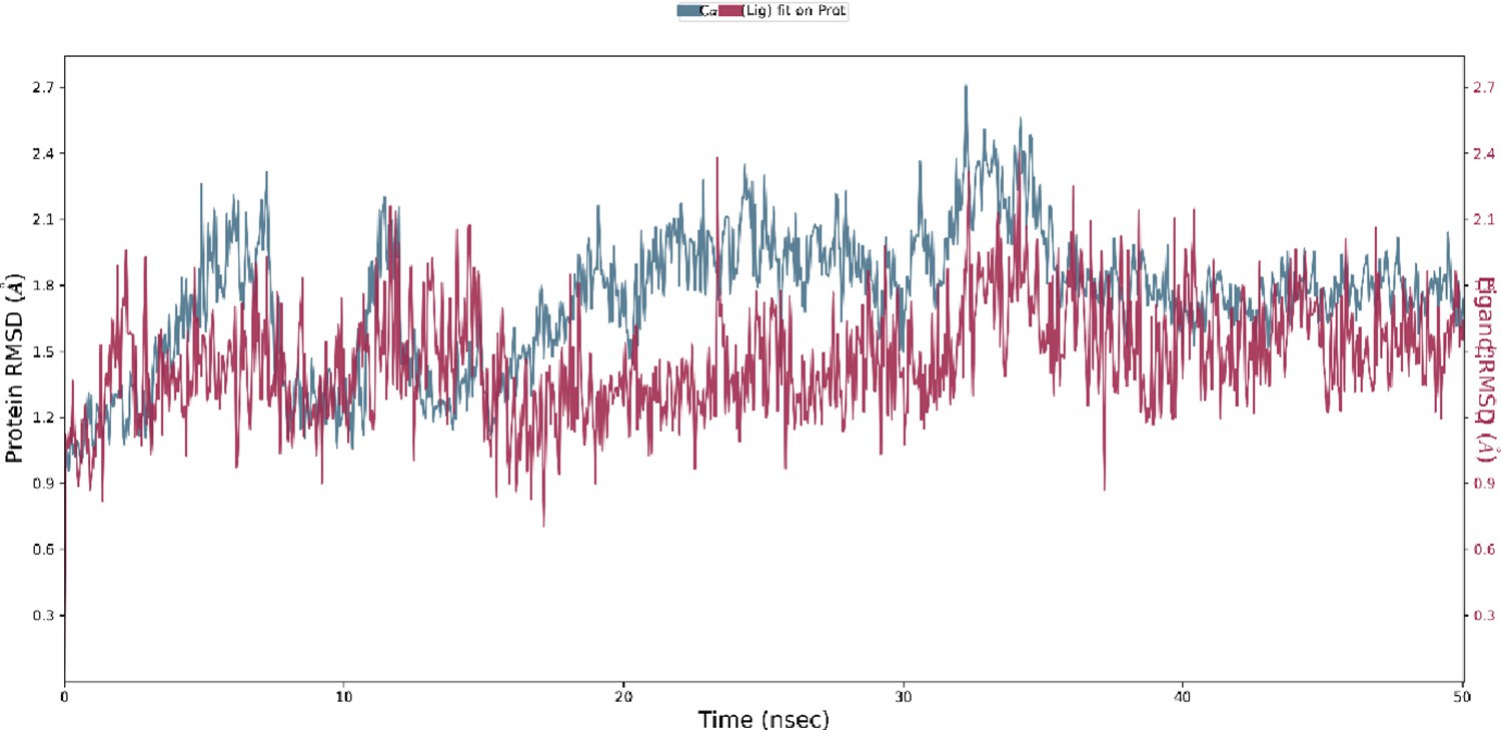
RMSD plot of 3OGN with 1c.

The RMSD values are indicative of the stability of complex structures. The optimal position of compound **1c**, as determined by the highest docking score at 50 ns MD generated by AutoDock Vina, was selected for further analysis. After examining the RMSD plot of 3OGN with **1c**, it was determined that the complex remained stable between 30 and 40 ns, as well as between 20 and 40 ns. This was because the peak fluctuation of the Cα 3OGN protein and the heavy atoms of the ligand fell within the range depicted in Fig 7. RMSD analysis of 3OGN in the presence of **1c** revealed its stability.

### Root Mean Square Fluctuation (RMSF) analysis

**Fig 8:**
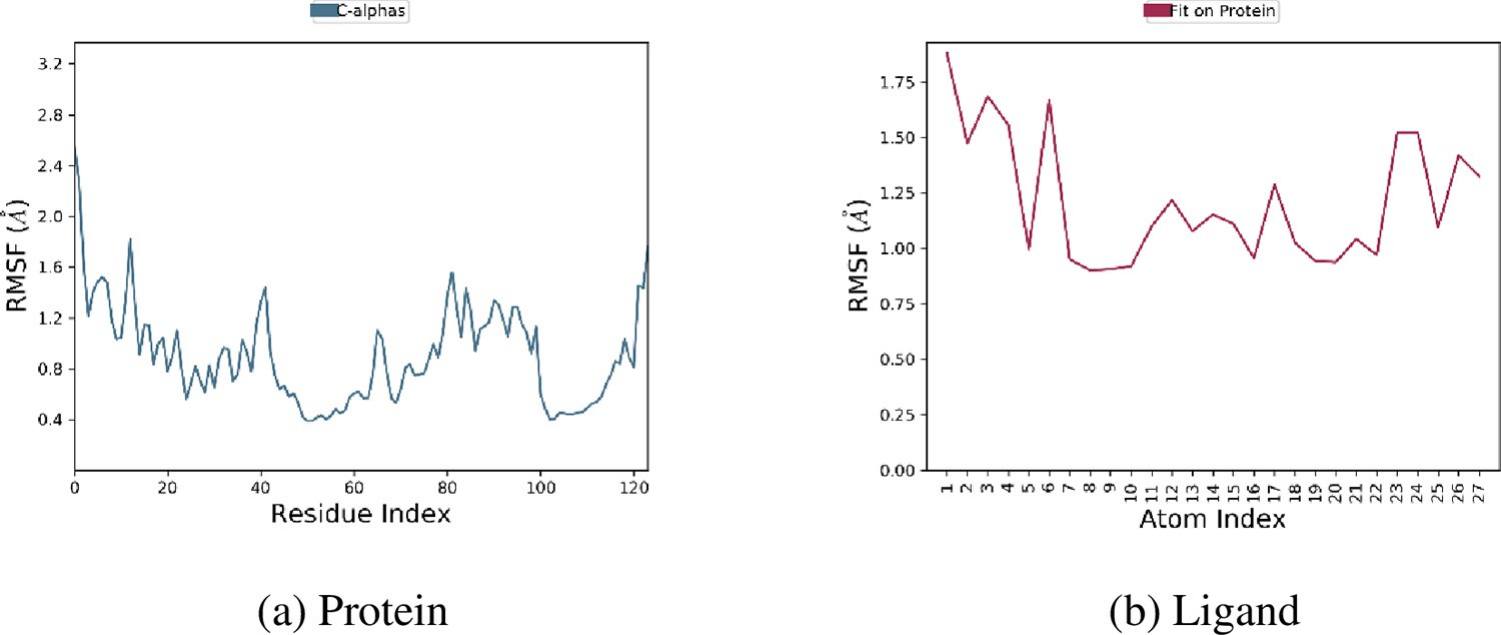
RMSF plot of 3OGN with 1c.

**Fig 9:**
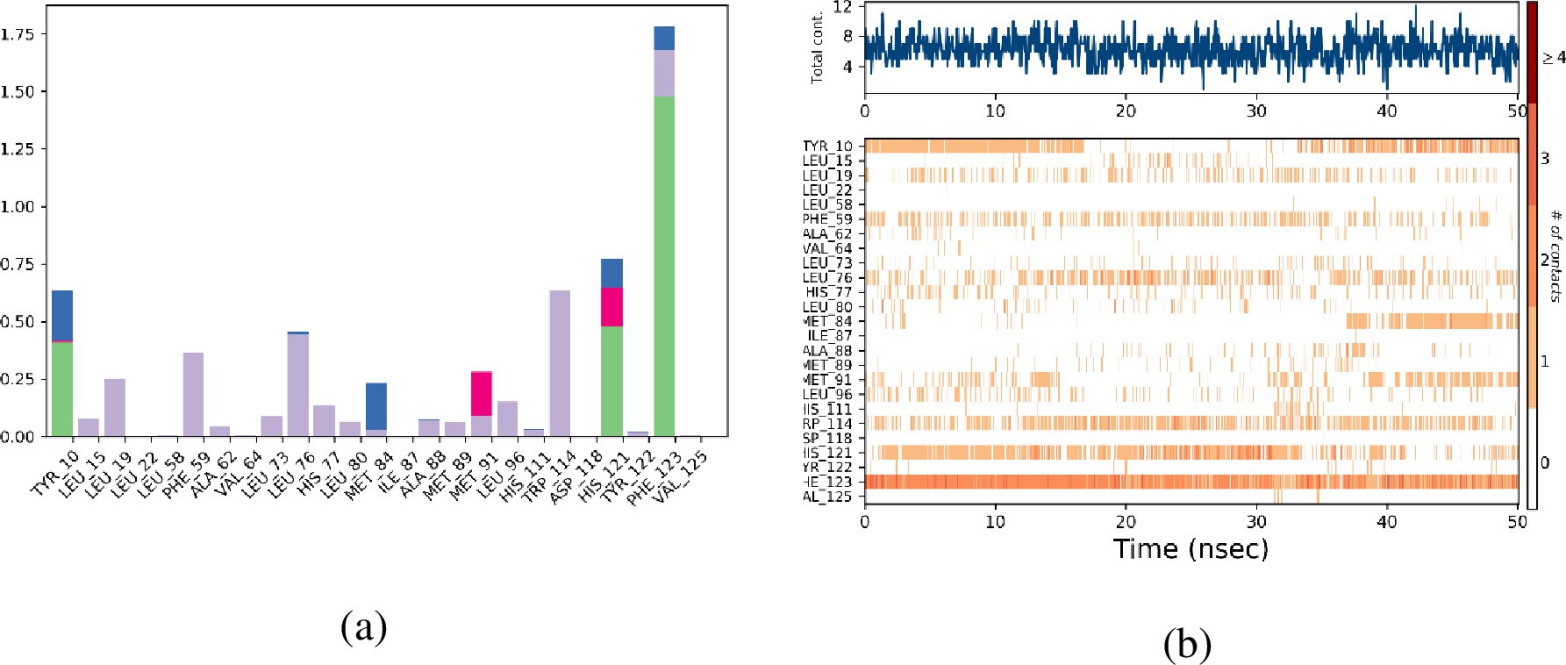
Histogram (a), and Timeline (b) representation of protein-ligand contacts of 3OGN with **1c**.

Alterations in the simulation’s protein chain were assessed using RMSF analysis. No fluctuations were observed in the amino acid residues, except for the *N*- and *C*-terminal residues. All residues were within an unacceptable range (Fig 8A and 8B). Based on this MD simulation analysis, compound **1c** were stable and exhibited good interactions with important protein residues and timeline (Fig 9A and 9B). Therefore, these compounds may be effective inhibitors of the 3OGN proteins.

### DFT calculation

**Fig 10:**
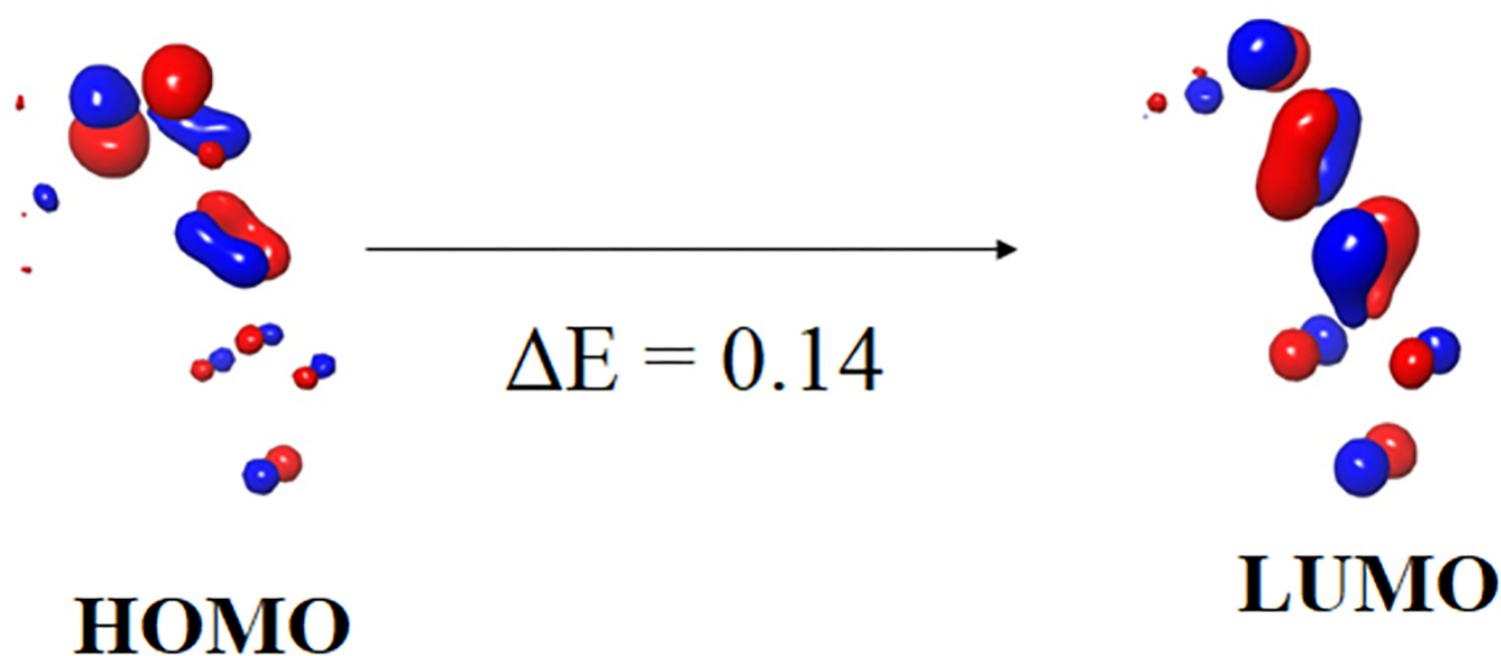
HOMO-LUMO energy diagram of compound 1c.

**Fig 11:**
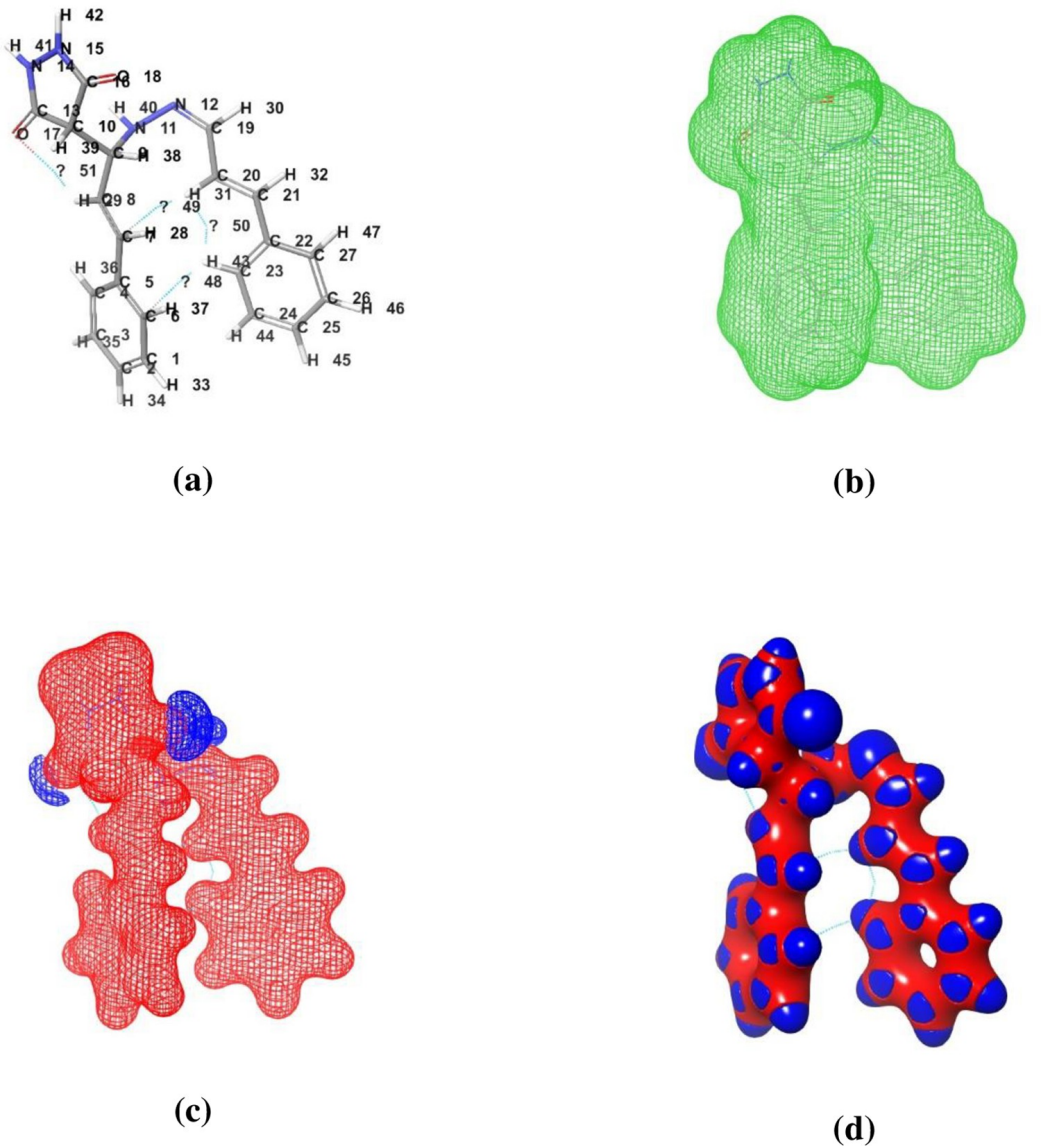
Representation of (a) compound with atomic number, (b) Electron density, (c) Electrostatic potential, and (d) Interaction strength.

**Fig 12:**
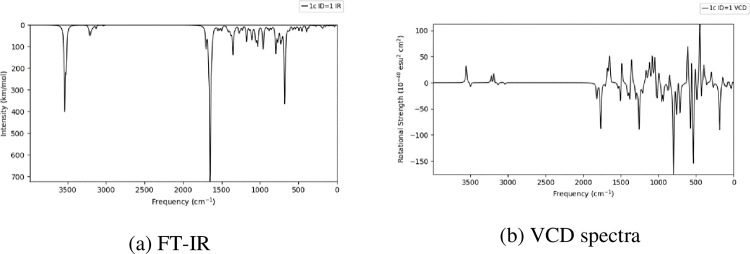
Shows the theoretical FT-IR (a) and VCD spectra (b) spectra of highly active compound 1c.

The B3LYP/6-31G (d, p) basis set was used to theoretically investigate the HOMO-LUMO, and FT-IR spectra [34, 40]. The highly active compound **1c** energy gap (ΔE) was 0.14 (Fig 10). Fig 11 shows the representation of (a) compound with atomic number, (b) electron density, (c) electrostatic potential, and (d) interaction strength of compound 1c. Fig 12 shows the theoretical FT-IR (a) and VCD spectra (b) spectra of highly active compound **1c**. A detailed discussion is provided in the supporting information file.

## Conclusions

In this study, a novel one-pot multicomponent synthesis of pyrazolidine-3,5-dione derivatives (**1a-m**) was achieved via the grindstone method using Cu(II)-Tyr as a catalyst under mild reaction conditions, resulting in high yields (84–96%). The synthesised compounds (**1a-m**) were screened for larvicidal and antifeedant activities against *C*. *quinquefasciatus* and the marine fish *O*. *mossambicus*. In larvicidal activity, compound **1c** (LD_50_ value of 9.7 μg/mL) exhibited higher activity than standard permethrin (LD_50_ value of 17.1 μg/mL) and showed lower toxicity (0% mortality at 100 μg/mL) in antifeedant activity. The highly active compound **1c** was investigated by molecular docking using 3OGN, displaying higher binding affinity (-10.4 kcal/mol) than standard permethrin (-9.5 kcal/mol), and MD simulations were discussed. DFT calculations were performed on the highly active compound **1c**, and the HOMO-LUMO, and Fourier transform infrared (FTIR) values were calculated and discussed. This investigation concluded that the pyrazolidine-3,5-dione derivative of compound **1c** was the most effective insecticide, suggesting these compounds may serve as larvicidal agents and eco-friendly pesticides for pest control.

## Supporting information

S1 File (PDF)
