# Supplementary material for: Cu(II)-tyrosinase enzyme catalyst mediated synthesis of mosquito larvicidal active pyrazolidine-3,5-dione derivatives with molecular docking studies and their ichthyotoxicity analysis
Source: PLoS One. 2024 Sep 19;19(9):e0298232. doi: 10.1371/journal.pone.0298232 (PMC11412543; doi:10.1371/journal.pone.0298232)
Supplement: S1 File — (PDF) [file pone.0298232.s001.pdf]

Supporting Information

Cu(II)-tyrosinase enzyme catalyst mediated synthesis of mosquito larvicidal active pyrazolidine-3,5-dione derivatives with molecular docking studies and their Ichthyotoxicity analysis

Velmurugan Loganathan [1¶], Radhakrishnan SurendraKumar [1¶], Anis Ahamed [2¶], Raman Gurusamy [3¶], Omar H Abd-Elkader [4¶], and Akbar Idhayadhulla [1¶*]

[1] Research Department of chemistry, Nehru Memorial College (Affiliated Bharathidasan University), Puthanampatti, Tamilnadu, India

[2] Department of Botany and Microbiology, College of Science, King Saudi University, Riyadh, Saudi Arabia

[3] Department of Life Science, Yeungnam University, Gyeongsan, Gyeongbuk-do, South Korea

[4] Department of Physics and Astronomy, College of Science, King Saudi University, Riyadh, Saudi Arabia

* Corresponding author

E-mail: a.idhayadhulla@gmail.com (A.I)

S. No.	Contents	Page no.
**1**	**Experimental section**	**3-11**
	Physical values, Spectral, Mass, and Analytical values	3-8
	Larvicidal activity	9
	Antifeedant activity	9
	Molecular Docking Studies	10
	DFT Calculation	10-11
**2**	**Results and Discussion**	**12-40**
	**Figure S1-S53:** ^{1}H , and ^{13}C NMR spectrum of Compound **(1a-1m)**	12 - 38
	**Table S1:** DFT Calculation	39-40

Experimental Section

Physical values, Spectral, Mass, and Analytical values

(*E*)-4-(1-hydrazinyl-3-phenylallyl)pyrazolidine-3,5-dione (1a)

White solid; Yield; 96%; mp = 110-112 °C; R_f=0.42; IR (KBr) *v*: (-NH) 3537.93, (-CH) 3011.48, (-CO) 1767.08, (-C=C-) 1620.58 cm^{-1}; ^{1}H NMR (300 MHz): δ 8.0 (s, -NH, 1H), 8.0 (s, -NH, 1H), 7.40-7.24(m, Ph-ring, 5H), 6.56 (s, Ph-CH, 1H), 6.19 CH-, s, 1H), 3.75 (s, -NH-CH-, 1H), 3.64 (s, CO-CH-, 1H), 2.0 (NH, s, 1H), 2.0 (-NH_2, s, 2H); ^{13}C NMR (75 MHz,): 174.8 (2-CO, 2C), 136.4, 128.6, 128.5, 127.9 (Ph-ring, 6C), 134.4 (Ph-C, 1C), 128.8 (=C-, 1C), 65.6 (-CH-, 1C), 55.7 (NH-C-, 1C); EIMS (*m/z*): 246.27 (M^+,14%); Anal. Calcd. For $C_{12}H_{14}N_4O_2$: C, 58.53; H, 5.73; N, 22.75%; Found: C, 58.51; H, 5.71; N, 22.73%.

4-((*E*)-1-((*E*)-2-benzylidenehydrazinyl)-3-phenylallyl)pyrazolidine-3,5-dione (1b)

Greenish solid; Yield; 91%; mp =145-148°C; R_f=0.40; IR (KBr) *v*: (-NH) 3535.63, (-CH) 3050.44, (-CO) 1750.33, (-C=C-) 1655.20 cm^{-1}; ^{1}H NMR (300MHz,): δ 8.36 (s, Ar-CH, 1H), 8.0 (-NH, s, 1H), 8.0 (-NH, s, 1H), 7.83-7.52 (m, Ar-ring, 5H), 7.40-7.24 (m, Ph-ring, 5H), 6.56 (s, Ph-CH, 1H), 6.19 (s, =CH-, 1H), 3.75 (s, -NH-CH-, 1H), 3.64 (s, CO-CH-, 1H), 2.0 (-NH, s, 1H); ^{13}C NMR (75 MHz): 174.8 (2-CO, 2C), 143.3 (Ar-ring, 1C), 136.4, 128.6, 128.5, 127.9 (Ph-ring, 6C), 134.4 (Ph-C, 1C), 133.7, 131.0, 129.2, 128.8 (Ar-ring, 6C), 128.8 (=C-, 1C), 65.6 (-CH-, 1C), 52.6 (NH-C-, 1C); EIMS(*m/z*) 333.15 (M^+,21%); Anal. Calcd. For $C_{19}H_{18}N_4O_2$: C, 68.25; H, 5.43; N, 16.76%; Found: C, 68.22; H, 5.41; N, 16.77%.

4-((*E*)-3-phenyl-1-((*E*)-2-((*E*)-3-phenylallylidene)hydrazinyl)allyl) pyrazolidine-3,5-dione (1c)

Light green powder; Yield; 83%; mp = 148-151°C; R_f=0.46; IR (KBr) *v:* (-NH) 3537.87, (-CH) 3048.44, (-CO) 1720.29, (-C=C-) 1610.24 cm^{-1}; ^{1}H NMR (300 MHz,): δ 8.0 (s, -NH, 1H), 8.0 (s, -NH, 1H), 7.60-7.33 (m, Ar-ring, 5H), 7.50 (s, =CH-, 1H), 7.40-7.24 (m, Ph-ring, 5H), 7.22 (s, Ph-CH, 1H), 7.0 (s, NH, 1H), 6.85 (s, N=CH, 1H), 6.56 (s, Ph-CH, 1H), 6.19 (s, =CH-, 1H), 3.75 (s, -NH-CH-, 1H), 3.64 (s, CO-CH-, 1H); ^{13}C NMR (75MHz,): 174.8 (2-CO, 2C), 137.2 (-N=C-, 1C), 136.4, 128.6, 128.5, 127.9 (Ph-ring, 6C), 135.2, 128.6, 128.5, 127.9 (Ar-ring, 6C), 134.4 (-Ph-C, 1C), 134.1 (Ar-C, 1C), 128.8 (=C-, 1C), 126.3 (=C-, 1C), 65.6 (-CH-, 1C), 52.6 (NH-CH_2, 1C); EIMS(*m/z*): 360.41 (M^+, 24.5%); Anal. Calcd. For $C_{21}H_{20}N_4O_2$: C, 69.98; H, 5.59; N, 15.55 %; Found: C, 69.99; H, 5.61; N, 15.56 %.

(*E*)-4-(3-phenyl-1-(2-phenylhydrazinyl)allyl)pyrazolidine-3,5-dione (1d)

White powder; Yield; 87%; mp =143-145°C; R_f=0.46; IR (KBr) *v*: (-NH) 3540.50, (-CH) 3044.50, (-CO) 1765.09, (-C=C-) 1625.40 cm^{-1}; ^{1}H NMR (300MHz,): δ 8.0 (s, -NH, 1H), 8.0 (s, -NH, 1H), 7.40-7.24 (m, Ph-ring, 5H), 7.37-6.90 (m, Ar-ring, 5H), 6.56 (s, Ph-CH, 1H), 6.19 (s, =CH-, 1H), 3.74 (s, -NH-CH-, 1H), 3.64 (s, CO-CH-, 1H), 4.0(NH, s, 1H), 2.0 (s, -NH, 1H); ^{13}C NMR (75 MHz,): 174.8 (2-CO, 2C), 151.0, 129.2, 122.8, 113.2 (Ar-ring, 6C), 136.2, 128.6, 128.5, 127.9 (Ph-ring, 6C), 134.3 (Ph-C, 1C), 128.7 (=C-, 1C), 65.4 (-CH-, 1C), 53.1 (NH-CH, 1C); EIMS(*m/z*): 322.36 (M^+, 19.8%); Anal. Calcd. for $C_{18}H_{18}N_4O_2$: C, 67.07; H, 5.63; N, 17.38 %; Found: C, 67.08; H, 5.61; N, 17.40 %.

(*E*)-4-(3-phenyl-1-(phenylamino)allyl)pyrazolidine-3,5-dione (1e)

Yellow powder; Yield; 91%; mp = 101-103°C; R_f=0.41; IR (KBr) *v*: (-NH) 3495.40, (-CH) 3010.45, (-CO) 1735.07, (-C=C-) 1655.42 cm^{-1}; ^{1}H NMR (300MHz,): δ 8.0 (-NH, s, 1H),

8.0 (-NH, s, 1H), 7.40-7.24 (m, Ph-ring, 5H), 7.23-6.83 (m, Ar-ring, 5H), 6.56 (s, Ph-CH, 1H), 6.20 (s, =CH-, 1H), 3.75 (s, -NH-CH-, 1H), 3.64 (s, CO-CH-, 1H), 4.0 (s, NH, 1H); ^{13}C NMR (75 MHz,): 174.8 (2-CO, 2C), 147.6, 129.5, 120.8, 119.7 (Ar-ring, 6C), 136.4, 128.6, 128.5, 127.9 (Ph-ring, 6C), 134.3 (Ph-C, 1C), 128.8 (=C-, 1C), 67.6 (-CH-, 1C), 53.7 (-NH-C-, 1C); EIMS(*m/z*): 307.13 (M^+, 19.7%); Anal. Calcd. for $C_{18}H_{17}N_3O_2$: C, 70.34; H, 5.58; N, 13.67 %; Found: C, 70.35; H, 5.60; N, 13.69 %.

(*E*)-1-benzylidene-3-((*E*)-1-(3,5-dioxopyrazolidin-4-yl)-3-phenylallyl)urea (1f)

Brown powder; Yield; 92%; mp = 111-119°C; R_f=0.35; IR (KBr) *v*: (-NH) 3497.54, (-CH) 3044.20, (-CO) 1745.09, (-C=C-) 1680.22 cm^{-1}; ^{1}H NMR (300MHz) δ 8.17 (s, N=CH, 1H), 8.03 (s, NH, 1H), 8.0 (s, NH, 1H), 8.0 (s, -NH, 1H,), 7.83-7.53 (m, Ar-ring, 5H), 7.40-7.24 (m, Ph-ring, 5H), 6.56 (s, Ph-CH, 1H), 6.18 (s, =CH-, 1H), 4.72 (-NH-CH-, s, 1H), 4.03 (s, CO-CH-, 1H); ^{13}C NMR (75 MHz,): 174.8 (2-CO, 2C), 164.5 (-CO-, 1C), 163.7 (=CH-, 1C), 136.4, 128.6, 128.5, 127.9 (Ph-ring, 6C), 134.4 (Ph-C, 1C), 147.6, 129.5, 120.8, 119.7 (Ar-ring, 6C), 128.8 (=C-, 1C), 67.0 (-CH-, 1C), 47.3 (NH-CH, 1C); EIMS(*m/z*): 362.14 (M^+, 22%); Anal. Calcd. for $C_{20}H_{18}N_4O_3$: C, 66.29; H, 5.01; N, 15.46 %; Found: C, 66.30; H, 5.03; N, 15.43 %.

(*E*)-1-((*E*)-1-(3,5-dioxopyrazolidin-4-yl)-3-phenylallyl)-3-((*E*)-3-phenylallylidene)thiourea (1g)

Light yellow powder; Yield; 88%; mp =276-279°C; R_f=0.46; IR (KBr) *v*: (-NH) 3545.40, (-CH) 3052.24, (-CO) 1710.25, (-C=C-) 1674.20 cm^{-1}; ^{1}H NMR (300MHz,): δ 8.0 (-NH, s, 1H), 8.0 (-NH, s, 1H), 7.60-7.33 (m, Ar-ring, 5H), 7.50 (s, N=CH, 1H), 7.40-7.24 (m, Ph-ring, 5H), 7.22 (s, Ar-CH, 1H), 6.85 (s, =C-, 1H), 6.56 (s, Ph-CH, 1H), 6.19 (s, =CH-, 1H),

3.75 (s, -NH-CH-, 1H), 3.64 (s, CO-CH-, 1H), 2.0 (NH, s, 1H); ^{13}C NMR (75 MHz,): 189.3 (-CS-, 1C), 174.8 (2-CO, 2C), 163.7 (N=C, 1C), 136.4, 128.6, 128.5, 127.9 (Ph-ring, 6C), 135.2, 128.1, 128.4, 127.8 (Ar-ring, 6C), 134.4 (Ph-C, 1C), 133.3 (Ar-C, 1C) 128.8 (=C-, 1C), 119.9 (=C-, 1C), 67.7 (-CH-, 1C), 52.4 (NH-C-, 1C); EIMS(*m/z*) 404.13 (M^+, 26.1%); Anal. Calcd. for $C_{22}H_{20}N_4O_2S$: C, 65.33; H, 4.98; N, 13.85%; Found: C, 65.30; H, 4.95; N, 13.87%.

(*E*)-4-(3-phenyl-1-(*p*-tolylamino)allyl)pyrazolidine-3,5-dione (1h)

White powder; Yield; 84%; mp = 172-174°C; R_f=0.51; IR (KBr) *v*: (-NH) 3550.22, (-CH) 3084.22, (-CO) 1700.24, (-C=C-) 1679.25 cm^{-1}; ^{1}H NMR (300MHz,): δ 8.0 (-NH, s, 1H), 8.0 (-NH, s, 1H), 7.40-7.24 (m, Ph-ring, 5H), 7.01-6.48 (m, Ar-ring, 4H), 6.56 (s, Ph-CH, 1H), 6.19 (s, =CH-, 1H), 3.75 (s, -NH-CH-, 1H), 3.64 (s, CO-CH-, 1H), 4.0 (s, NH, 1H), 2.34 (s, -CH_3, 3H); ^{13}C NMR (75 MHz,): 174.8 (2-CO, 2C), 144.6, 129.8, 129.6, 113.4 (Ar-ring, 6C), 136.4, 128.6, 128.5, 127.9 (Ph-ring, 6C), 134.4 (Ph-C, 1C), 128.8 (=C-, 1C), 67.6 (-CH-, 1C), 53.7 (NH-C-, 1C), 21.3 (-CH_3, 1C); EIMS(*m/z*) 321.15 (M^+, 20.8%); Anal. Calcd. for $C_{19}H_{19}N_3O_2$: C, 71.01; H, 5.96; N, 13.08 %; Found: C, 71.00; H, 5.95; N, 13.05 %.

(*E*)-*N*-(1-(3,5-dioxopyrazolidin-4-yl)-3-phenylallyl)acetamide (1i)

Pale yellow powder; Yield; 86%; mp = 122-124°C; R_f=0.56; IR (KBr) *v*: (-NH) 3571.24, (-CH) 3044.20, (-CO) 1710.22, (-C=C-) 1670.42 cm^{-1}; ^{1}H NMR (300MHz,): δ 8.03 (-NH, s, 1H), 8.0 (s, NH, 1H), 8.0 (s, NH, 1H), 7.40-7.24 (m, Ph-ring, 5H), 6.56 (s, Ph-CH, 1H), 6.19 (s, =CH-, 1H), 4.72 (s, -NH-CH-, 1H), 4.03 (s, CO-CH-, 1H), 1.84 (s, -CH_3, 3H); ^{13}C NMR (75 MHz,): 174.8 (2-CO, 2C), 170.7 (CO, 1C), 136.4, 128.6, 128.5, 127.9 (Ph-ring, 6C), 134.4 (Ph-C, 1C), 128.8 (=C-, 1C), 67.5 (-CH-, 1C), 44.9 (NH-C-, 1C), 23.7 (-CH_3, 1C); EIMS(*m/z*) 273.11 (M^+, 16.3%); Anal. Calcd. for $C_{14}H_{15}N_3O_3$: C, 61.53; H, 5.53; N, 15.38 %; Found: C, 61.50; H, 5.55; N, 15.35 %.

(*E*)-*N*-(1-(3,5-dioxopyrazolidin-4-yl)-3-phenylallyl)benzamide (1j)

Brown powder; Yield; 83%; mp = 208-204°C; R_f=0.58; IR (KBr) *ν*: (-NH) 3565.42, (-CH) 3050.24, (-CO) 1730.45, (-C=C-) 1680.24 cm^{-1}; ^{1}H NMR (300MHz): δ 8.45 (s, NH, 1H), 8.03-7.70 (m, Ar-ring, 5H), 8.0 (s, -NH, 1H), 8.0 (s, -NH, 1H), 7.40-7.24 (m, Ph-ring, 5H), 6.56 (s, Ph-CH, 1H), 6.19 (=CH-, s, 1H), 4.72 (s, -NH-CH-, 1H), 4.03 (s, CO-CH-, 1H); ^{13}C NMR (75 MHz,): 174.8 (2-CO, 2C), 167.5 (CO, 1C), 136.4, 128.6, 128.5, 127.9 (Ar-ring, 6C), 134.2, 132.1, 128.8, 127.5 (Ph-ring, 6C), 134.4 (Ph-C, 1C), 128.8 (=C-, 1C), 67.5 (-CH-, 1C), 45.7 (NH-C-, 1C); EIMS(*m/z*): 335.36 (M^+, 20.9%); Anal. Calcd. for $C_{19}H_{17}N_3O_3$: C, 68.05; H, 5.11; N, 12.53 %; Found: C, 68.03; H, 5.10; N, 12.55 %.

(*E*)-1-benzylidene-3-((*E*)-1-(3,5-dioxopyrazolidin-4-yl)-3-phenylallyl)urea (1k)

Green solid; Yield; 92%; mp = 131-129°C; R_f=0.46; IR (KBr) *ν*: (-NH) 3540.24, (-CH) 3092.21, (-CO) 1725.34, (-C=C-) 1655.22 cm^{-1}; ^{1}H NMR (300MHz) δ 8.17 (s, N=CH, 1H), 8.03 (s, NH, 1H), 8.0 (s, NH, 1H), 8.0 (s, NH, 1H), 7.83-7.52 (m, Ar-ring, 5H), 7.40-7.24 (m, Ph-ring, 5H), 6.56 (s, Ph-CH, 1H), 6.19 (=CH-, s, 1H), 4.72 (s, -NH-CH-, 1H), 4.03 (s, CO-CH-, 1H); ^{13}C NMR (75 MHz): 174.8 (2-CO, 2C), 164.5 (CO, 1C), 163.7 (N=C, 1C), 136.4, 128.6, 128.5, 127.9 (Ph-ring, 6C), 134.4 (Ph-C, 1C), 133.7, 131.0, 129.2, 128.8 (Ar-ring, 6C), 128.8 (=C-, 1C), 67.0 (-CH-, 1C), 47.3 (NH-C-, 1C); EIMS(*m/z*): 362.14 (M^+, 22%); Anal. Calcd. for $C_{20}H_{18}N_4O_3$: C, 66.29; H, 5.01; N, 15.46 %; found: C, 66.30; H, 5.03; N, 15.45 %.

(*E*)-1-((*E*)-1-(3,5-dioxopyrazolidin-4-yl)-3-phenylallyl)-3-((*E*)-3-phenylallylidene)urea (1l)

White greenish powder; Yield; 94%; mp = 145-148°C; R_f=0.48; IR (KBr) *v*: (-NH) 3545.47, (-CH) 3036.22, (-CO) 1710.45, (-C=C-) 1650.39 cm^{-1}; ^{1}H NMR (300MHz,): δ 8.03 (s, NH, 1H), 8.0 (s, NH, 1H), 8.0 (s, NH, 1H), 7.60-7.33 (m, Ar-ring, 5H), 7.50 (s, N=CH, 1H), 7.40-7.24 (m, Ph-ring, 5H), 7.22 (s, Ar-CH, 1H), 6.85 (=CH-, s, 1H), 6.56 (s, Ph-CH, 1H), 6.19 (=CH-, s, 1H), 4.72 (s, -NH-CH-, 1H), 4.03 (s, CO-CH-, 1H); ^{13}C NMR (75 MHz,): 174.8 (2-CO, 2C), 164.5 (CO, 1C), 163.7 (N=C, 1C), 136.4, 128.6, 128.5, 127.9 (Ph-ring, 6C), 135.2, 128.6, 128.5, 127.9 (Ar-ring, 6C), 134.4 (Ph-C, 1C), 133.3 (Ar-CH, 1C), 128.8 (=C-, 1C), 119 (=C-, 1C), 67.0 (-CH-, 1C), 47.3 (NH-C-, 1C); EI-MS(*m/z*): 388.15 (M^+, 24.1%); Elemental analysis: Anal. Calcd. for $C_{22}H_{20}N_4O_3$: C, 68.03; H, 5.19; N, 14.42 %; Found: C, 68.05; H, 5.20; N, 14.45 %.

(*E*)-4-(1-(methylamino)-3-phenylallyl)pyrazolidine-3,5-dione (1m)

Light yellow powder; Yield; 84%; mp =96-99°C; R_f=0.47; IR (KBr) *v*: (-NH) 3550.24, (-CH) 3050.25, (-CO) 1755.20, (-C=C-) 1670.42 cm^{-1}; ^{1}H NMR(300MHz) δ 8.0 (-NH, s, 1H), 8.0 (-NH, s, 1H), 7.40-7.24 (m, Ph-ring, 5H), 6.56 (s, Ph-CH, 1H), 6.19 (=CH-, s, 1H), 3.75 (s, -NH-CH-, 1H), 3.64 (s, CO-CH-, 1H), 3.26 ($-CH_3$, s, 3H), 2.0 (s, NH, 1H); ^{13}C NMR (75 MHz,): 174.8 (2-CO, 2C), 136.4, 128.6, 128.5, 127.9 (Ph-ring, 6C), 134.4 (Ph-C, 1C), 128.8 (=C-, 1C), 67.7 (-CH-, 1C), 53.8 (NH-C-, 1C), 33.4 ($-CH_3$, 1C); EIMS(*m/z*): 245.12 (M^+, 14.3%); Anal. Calcd. for $C_{13}H_{15}N_3O_2$: C, 63.66; H, 6.16; N, 17.13 %; Found: C, 63.67; H, 6.19; N, 17.15 %.

Larvicidal activity

Synthesized compounds were tested against the urban mosquito larvae, *Culex quinquefasciatus*. Eggs of *C. quinquefasciatus* were obtained from the city drainage system. Eggs were placed in clean water and kept at room temperature for hatching. Larval development was monitored for 7 d. Second stage larvae were collected using a pasture pipette, placed in cotton to remove excess water, and transferred to test vials. Larval mortality was observed using increasing concentrations of synthesized compounds (10, 20, 30, and 40 µg/mL). The susceptibility or resistance of the mosquito larvae to the selected concentration of the synthesized compounds was determined with a standard protocol. The synthesised compounds were prepared at concentrations of 10 µg/mL, 25 µg/mL, 50 µg/mL, and 100 µg/mL. The ratio of dead to live larvae was determined as the percentage of activity (%).

Antifeedant activity

Fingerlings (1.5–2.0 cm) of marine acclimated *Oreochromis mossambicus* were used for evaluating antifeedant activity. Ten fingerlings were introduced in experimental and control glass bowls, each containing one L of seawater and the selected concentration of compound. Immediate reflex changes and mortality were observed. After 24 h of exposure, the number dead and live fish was counted.

Molecular Docking study

Molecular docking experiments were conducted to evaluate the binding and interaction of compound **1c**, permethrin, and 3OGN protein using AutoDock Vina 1.1.2 software (http://mgltools.scripps.edu). 3OGN's crystal structure was obtained from the Protein Data Bank (PDB) at https://www.rcsb.org/. The 3D structure of compound **1c** and permethrin was accomplished using ChemDraw Ultra 12.0 and ChemDraw3D Pro 12.0 software. The 3OGN protein was fixed at centre _x: 15.694, centre _y: 47.766, centre _z: 9.394 with dimension size_x: 20, size_y: 20, size_z: 20 with spacing of 1.0 Å. Eight was set as the exhaustiveness value. Other parameters were set to their default values for Vina docking and will not be discussed further. The compound that displayed the lowest binding affinity was the most superior, as evidenced by its superior score. This superior compound was assessed using Discovery Studio 2019 software through visual analysis.

DFT calculation

To understand the spectral assignments and study the molecular geometry and electronic transitions of the synthesised compound **1c** (highly active compound), a computational study was conducted to explore the molecular interactions in more detail. The Gaussian 09 program was used for the theoretical studies. Gradient-corrected correlation with Pople's basis set B3LYP/6-31G (d, p) was applied. The gauge independent atomic orbital (GIAO) and Integral Equation Formalism Polarisable Continuum Model (IEFPCM) with the same functional set were used to compute the ^{1}H-NMR chemical shifts in the liquid phase. The compound **1c** were calculated for highest occupied molecular orbital (HOMO) and lowest unoccupied molecular orbital (LUMO). The IR frequencies calculated using this method were found to be positive, indicating that the optimised structure is at a minimum on the potential energy surface. In the FTIR spectrum, bands that appeared were assigned with full accuracy

using animated modes of vibrations. The frontier molecular orbitals help determine the chemical stability of the system. Moreover, structure-based molecular properties such as atomic charges, total energy, stabilisation energy, electronic properties, bond lengths, frontier molecular orbitals, and molecular electrostatic potential were calculated using this theory in the gas phase. For visualisation of the obtained DFT results, ChemCraft 1.5 software was used.

Results and Discussion

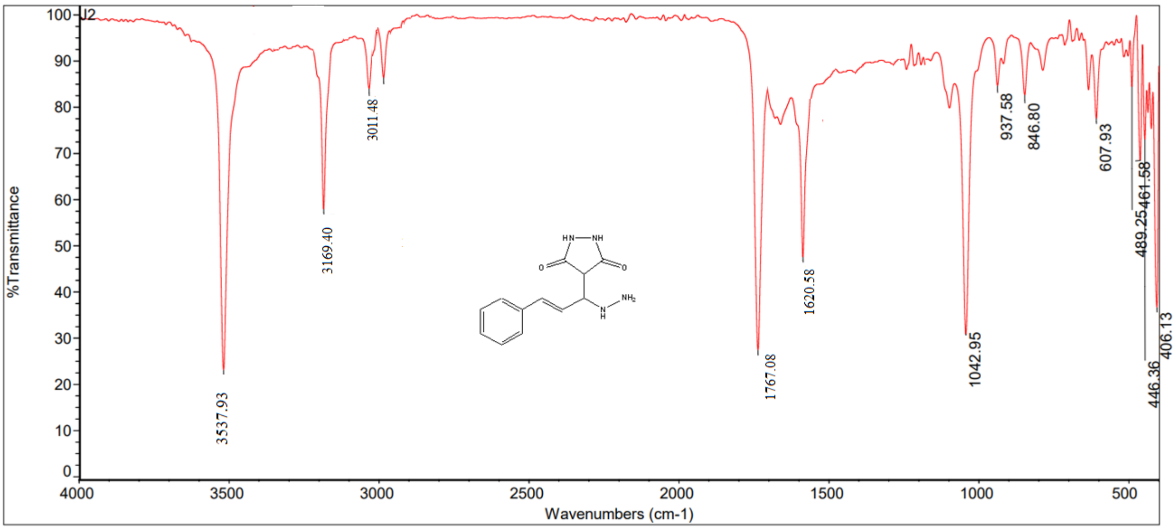

Figure S1 FTIR spectrum of the compound **1a**

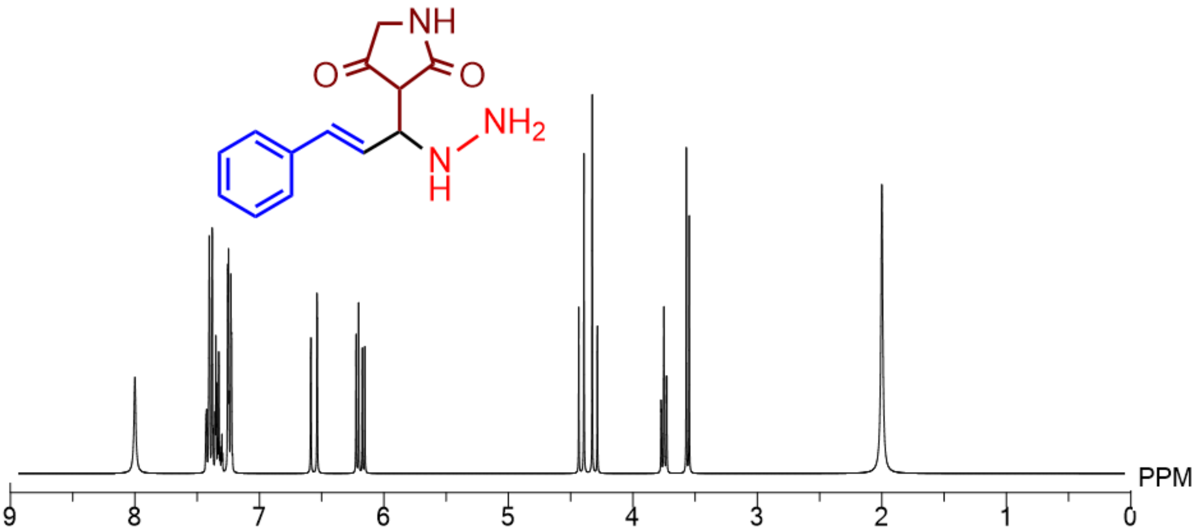

Figure S2 ^{1}H NMR spectrum of the compound **1a**

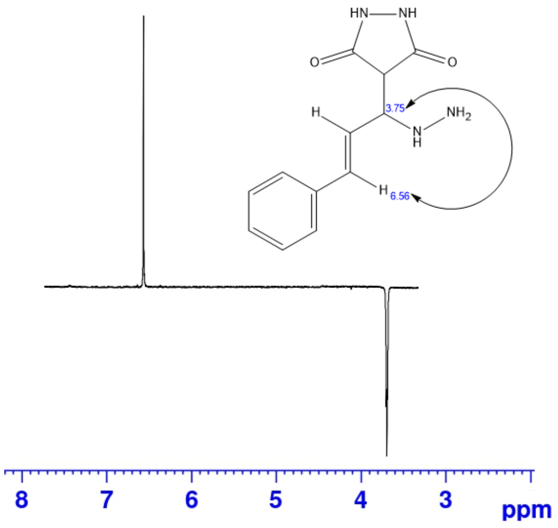

Figure S3 NOE data for compound 1a

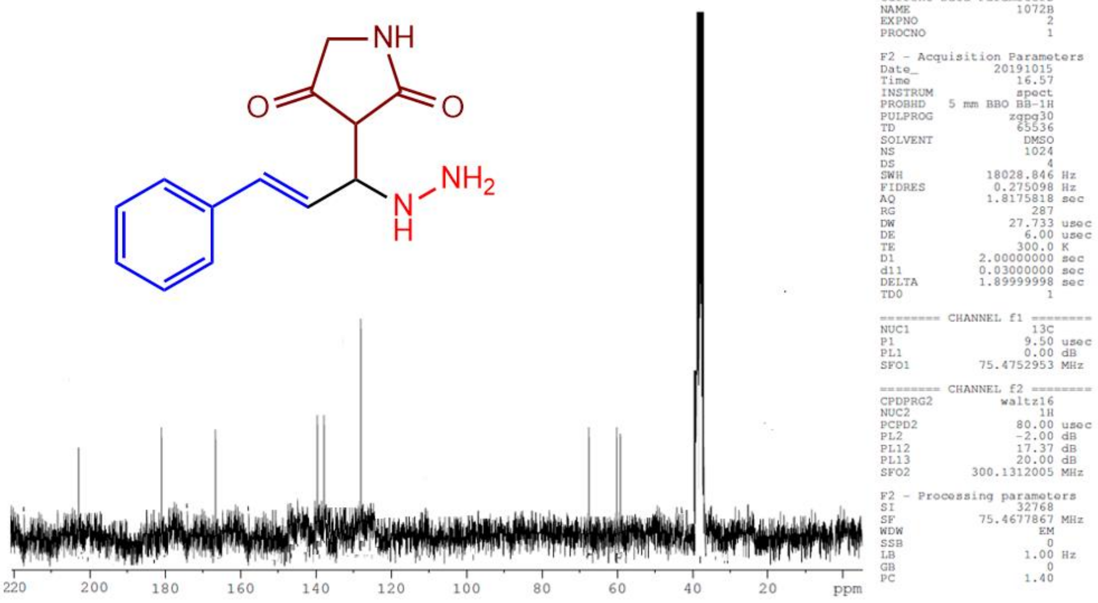

Figure S4 ^{13}C NMR spectrum of the compound **1a**

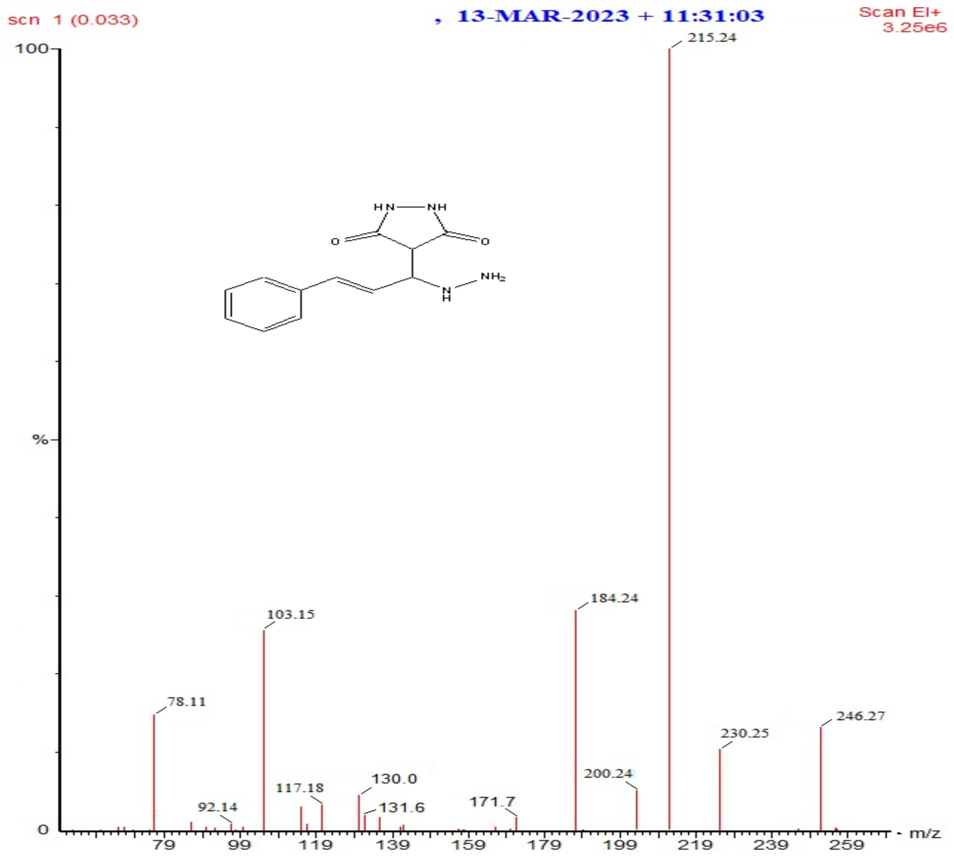

Figure S5 Mass spectrum of the compound **1a**

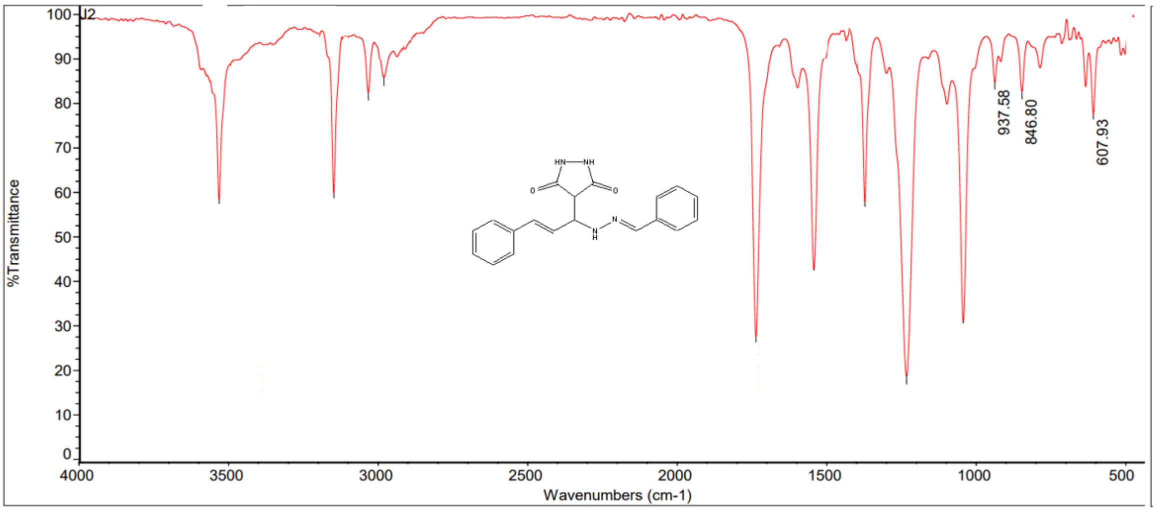

Figure S6 FTIR spectrum of the compound **1b**

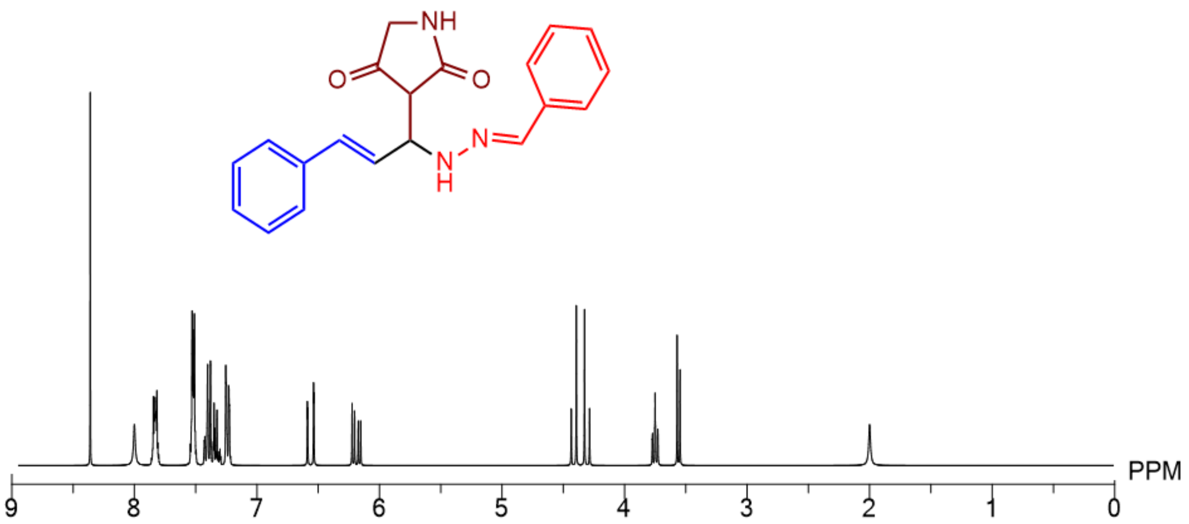

Figure S7 ^{1}H NMR spectrum of the compound **1b**

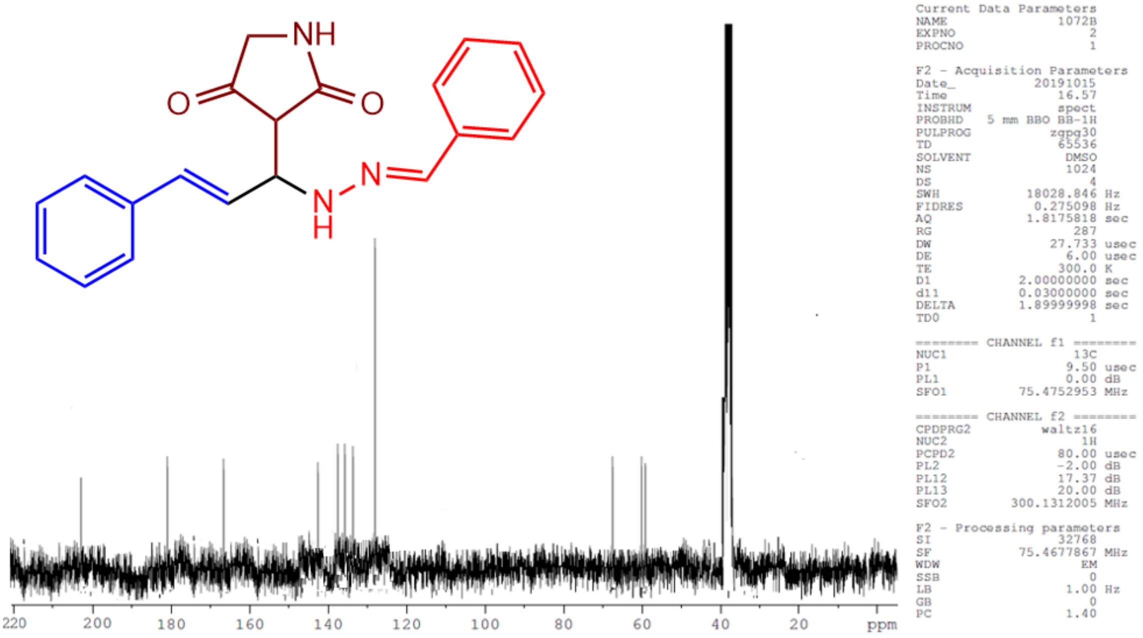

Figure S8 ^{13}C NMR spectrum of the compound **1b**

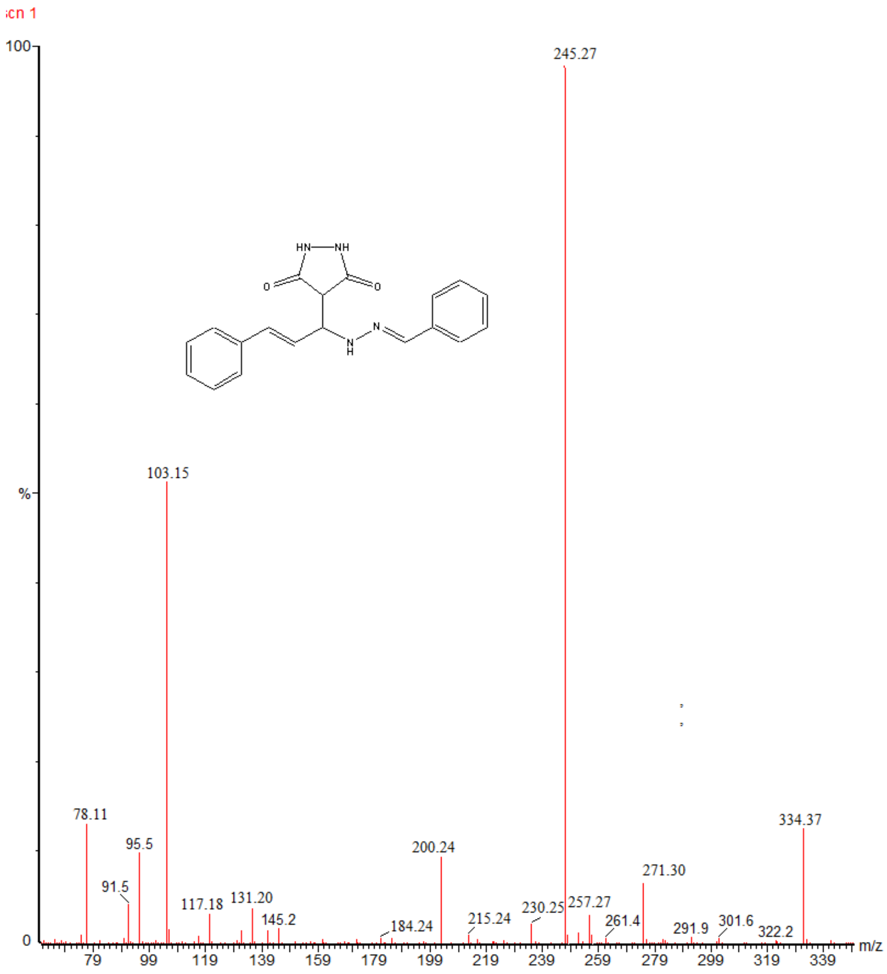

Figure S9 Mass spectrum of the compound **1b**

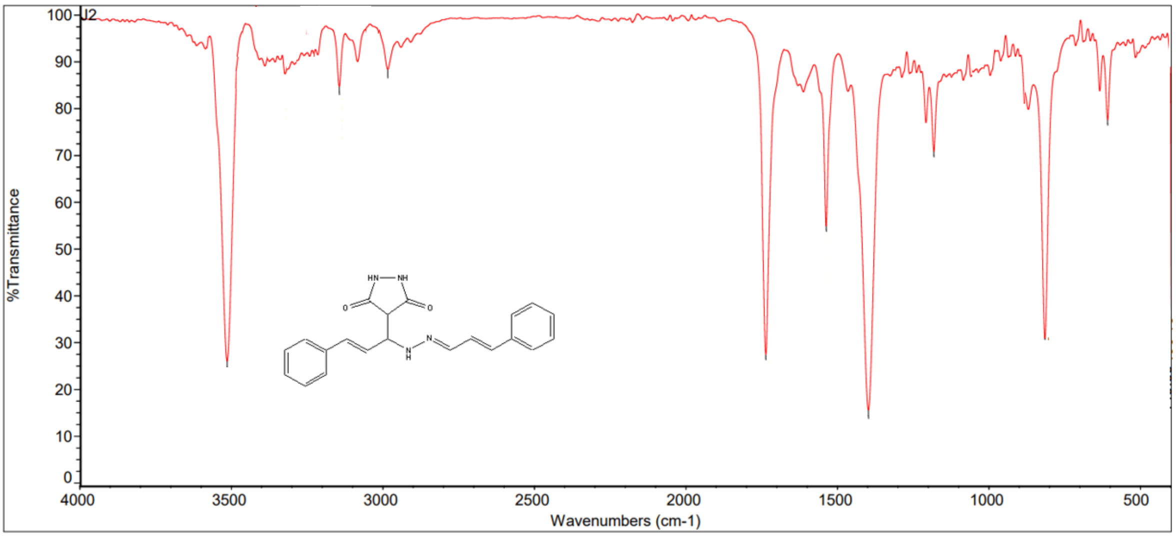

Figure S10 FTIR spectrum of the compound **1c**

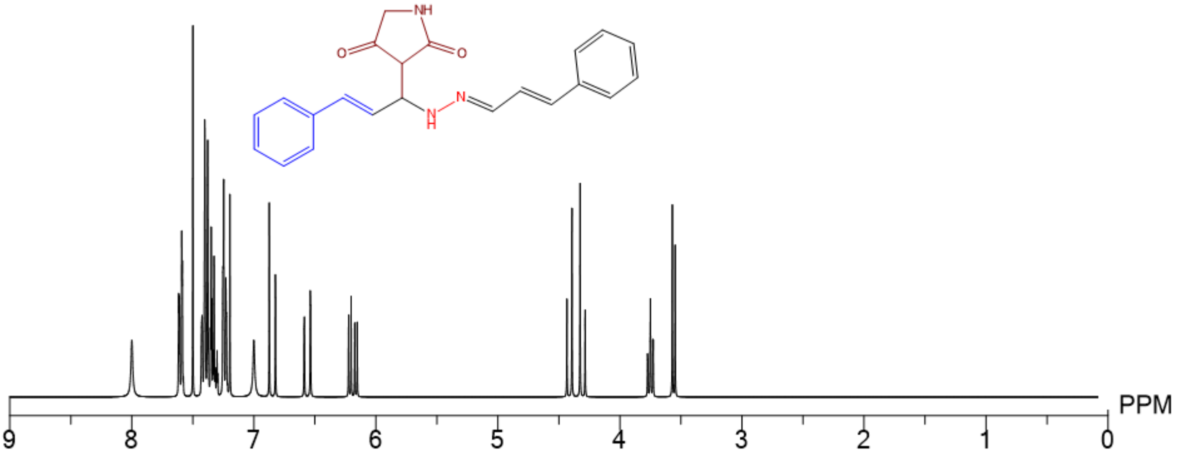

Figure S11 ^{1}H NMR spectrum of the compound **1c**

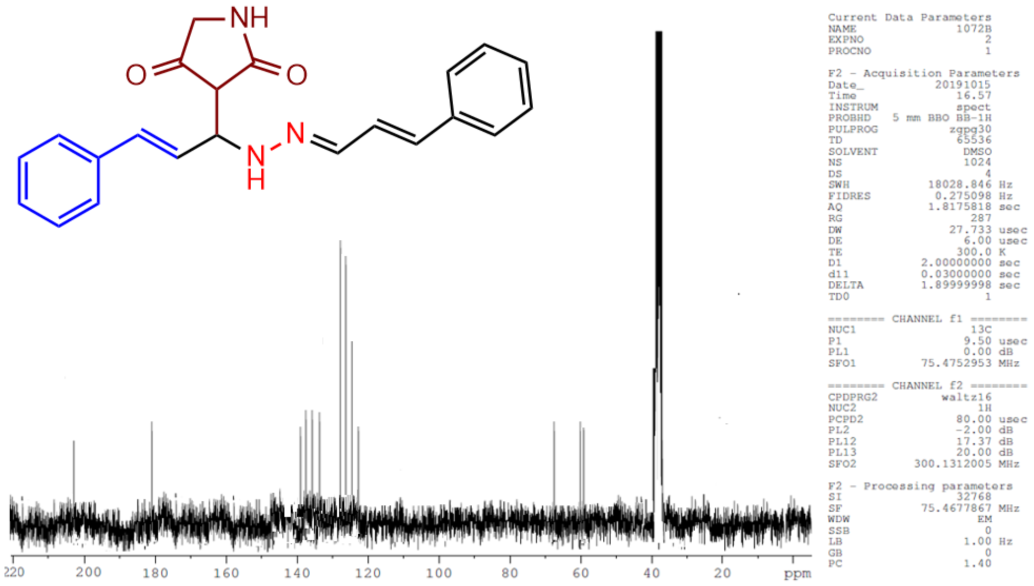

Figure S12 ^{13}C NMR spectrum of the compound **1c**

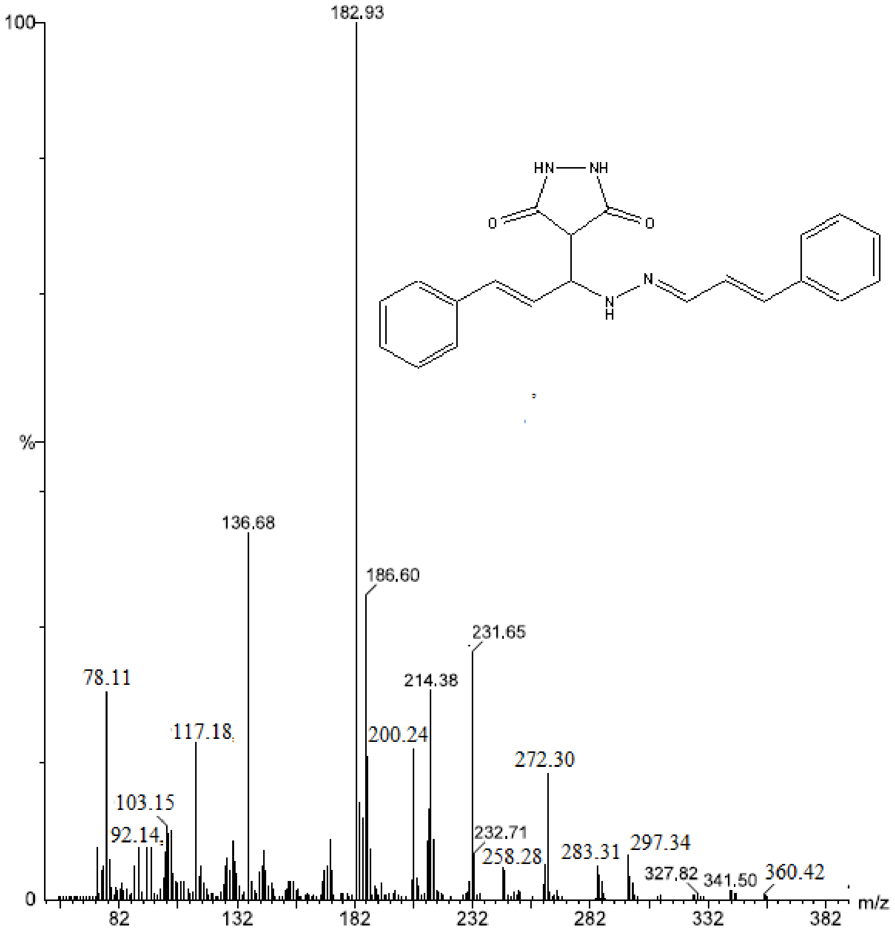

Figure S13 Mass spectrum of the compound **1c**

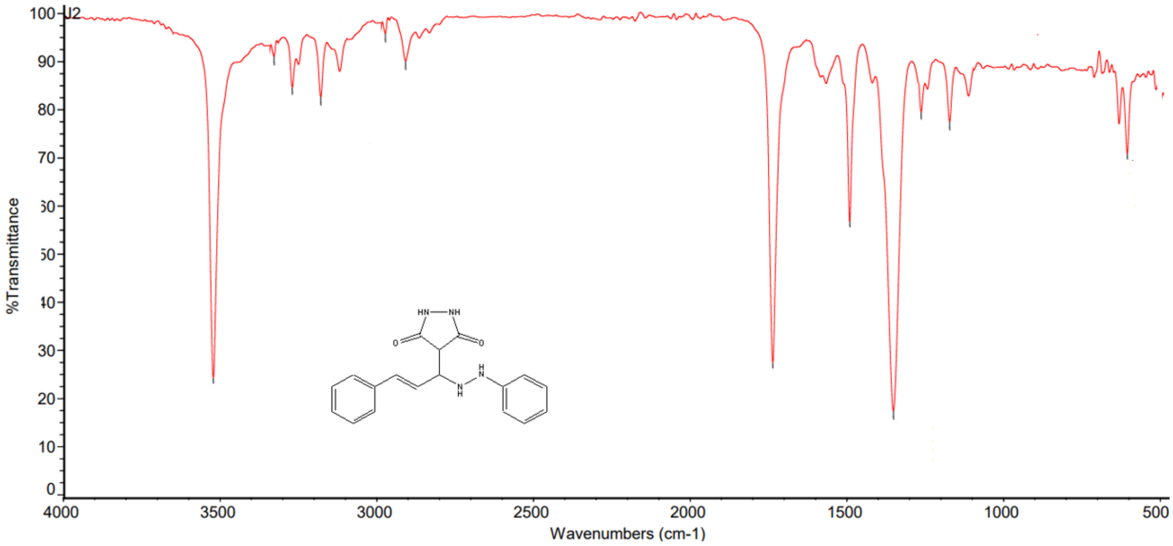

Figure S14 FTIR spectrum of the compound **1d**

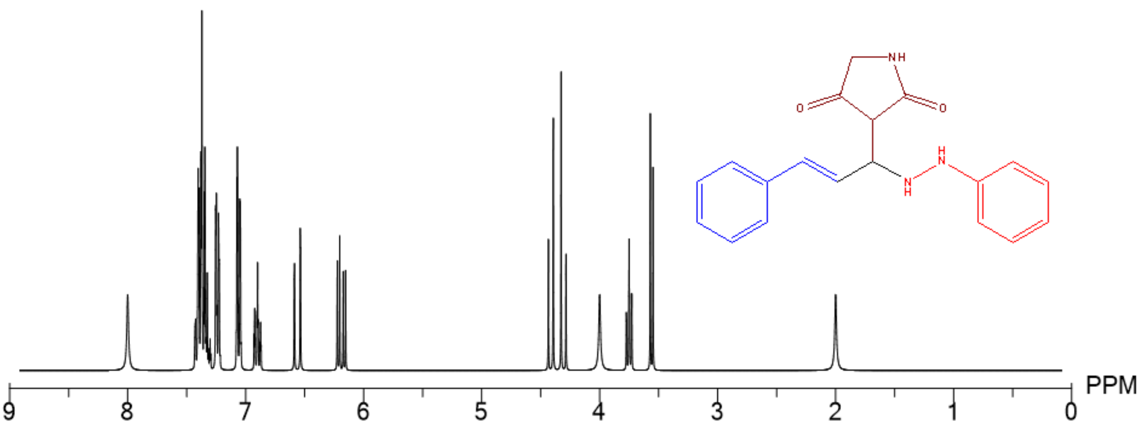

Figure S15 ^{1}H NMR spectrum of the compound **1d**

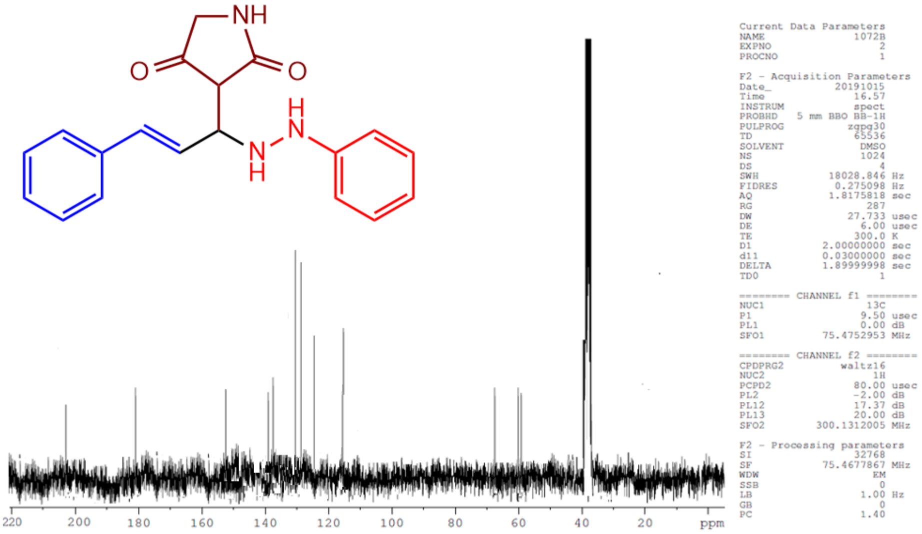

Figure S16 ^{13}C NMR spectrum of the compound **1d**

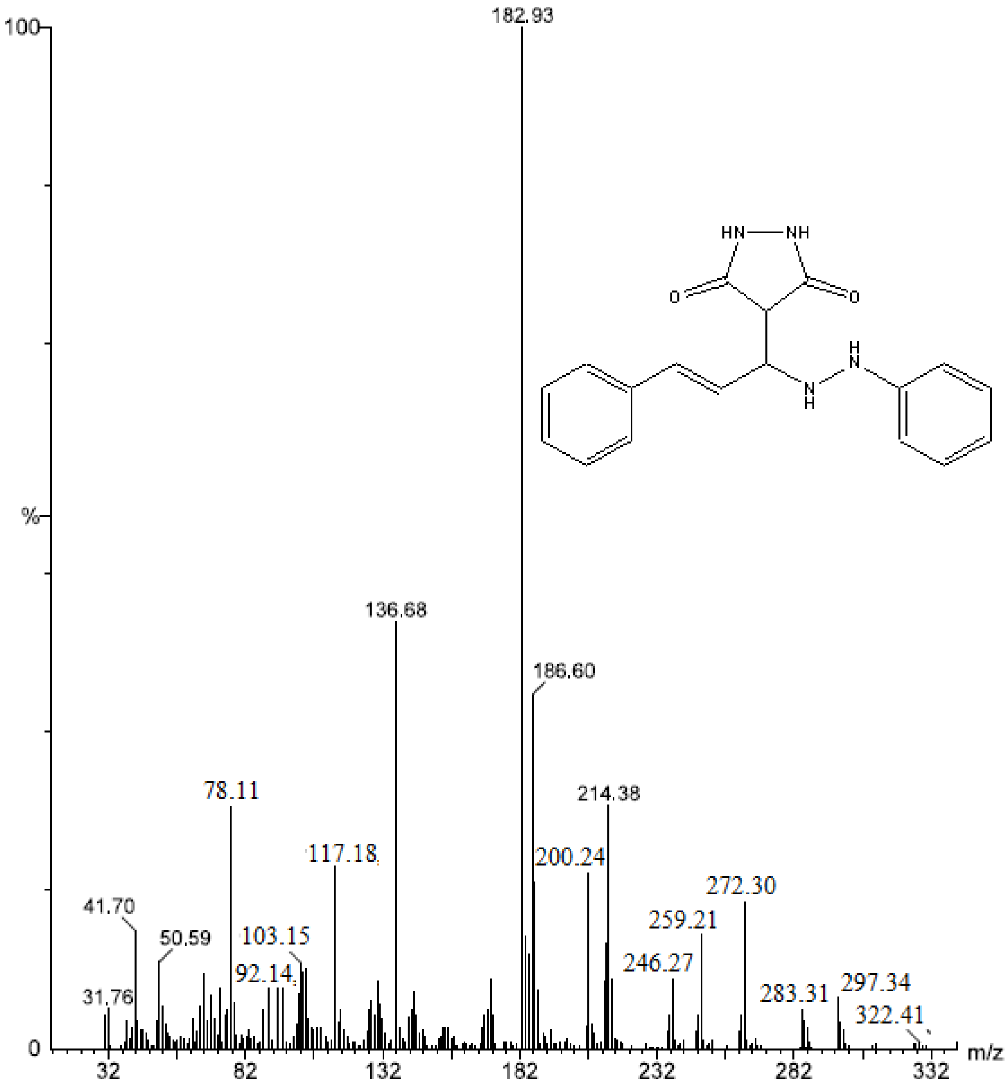

Figure S17 Mass spectrum of the compound **1d**

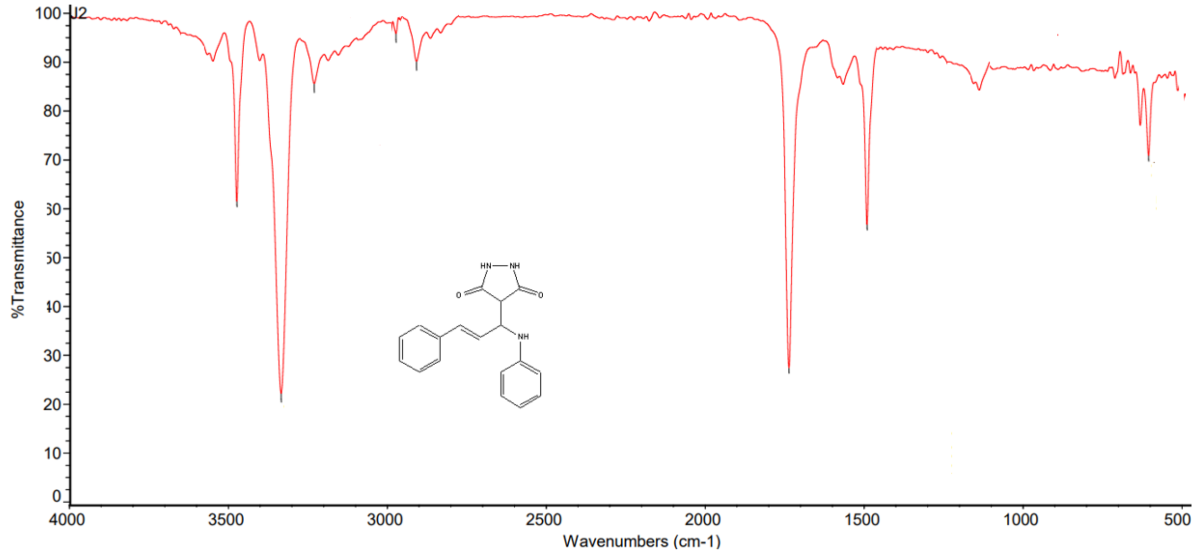

Figure S18 FTIR spectrum of the compound **1e**

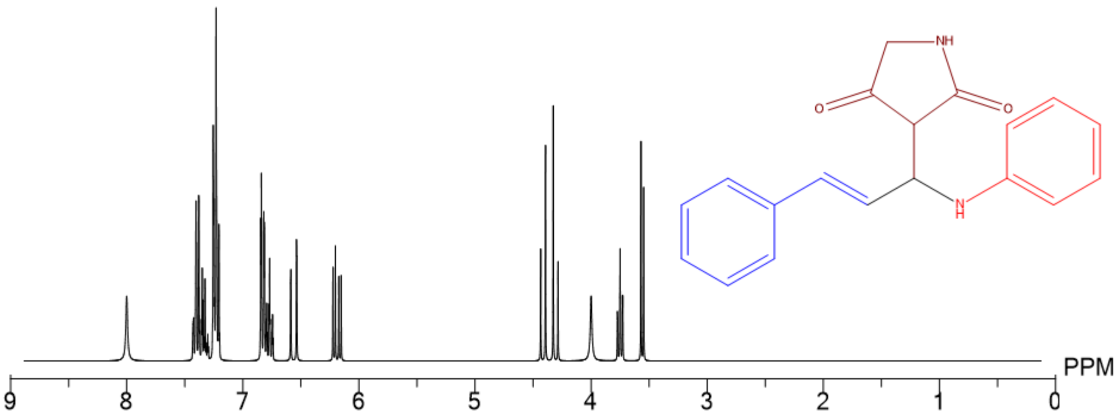

Figure S19 ^{1}H NMR spectrum of the compound **1e**

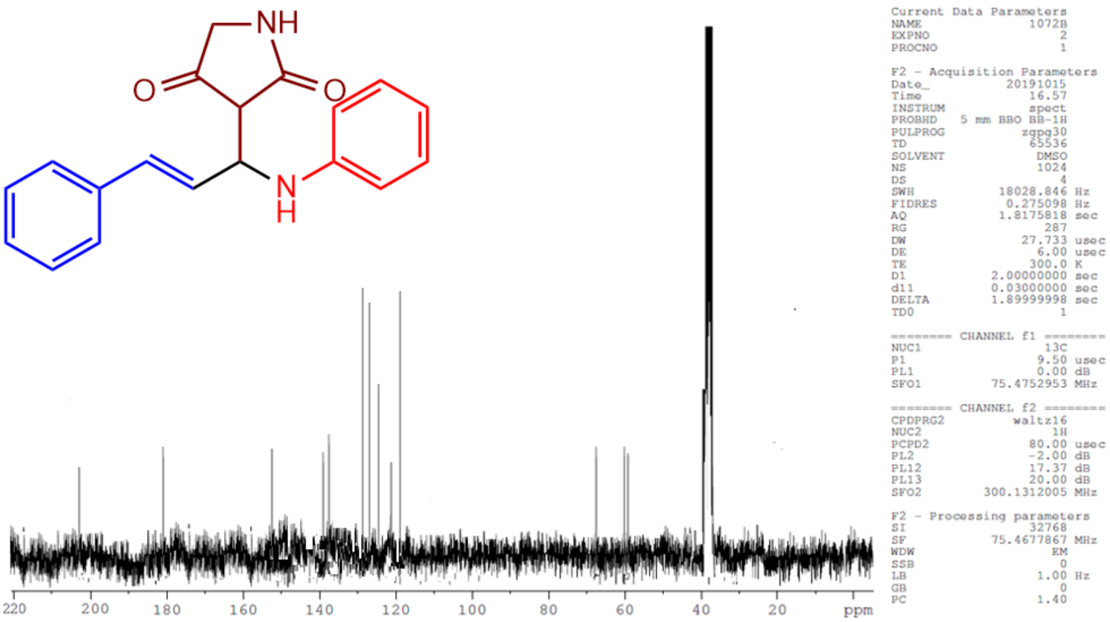

Figure S20 ^{13}C NMR spectrum of the compound **1e**

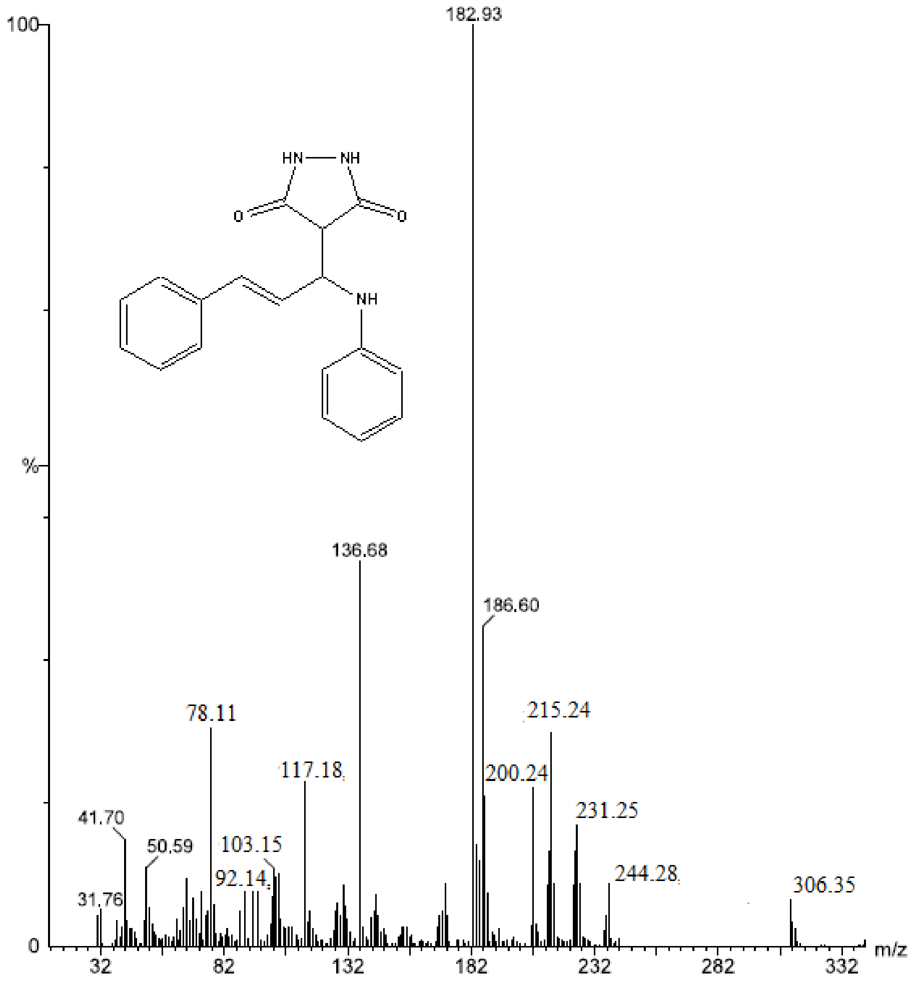

Figure S21 Mass spectrum of the compound **1e**

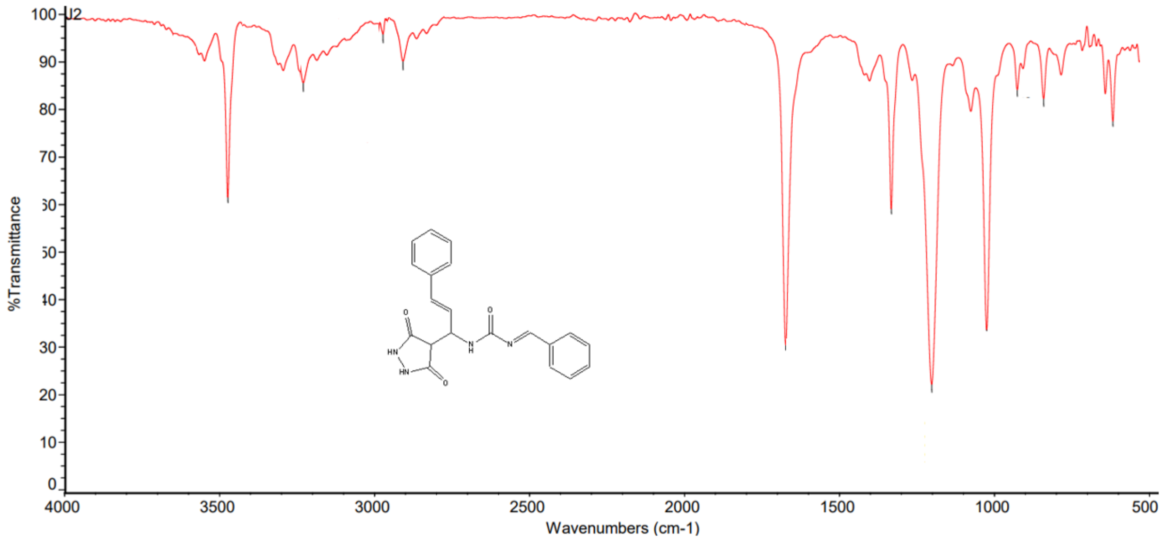

Figure S22 FTIR spectrum of the compound **1f**

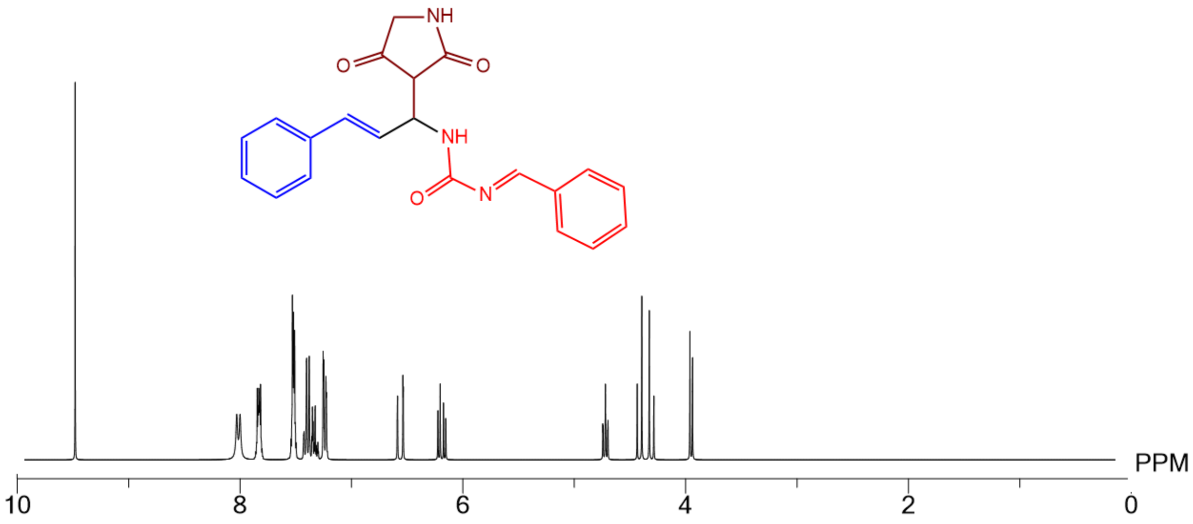

Figure S23 ^{1}H NMR spectrum of the compound **1f**

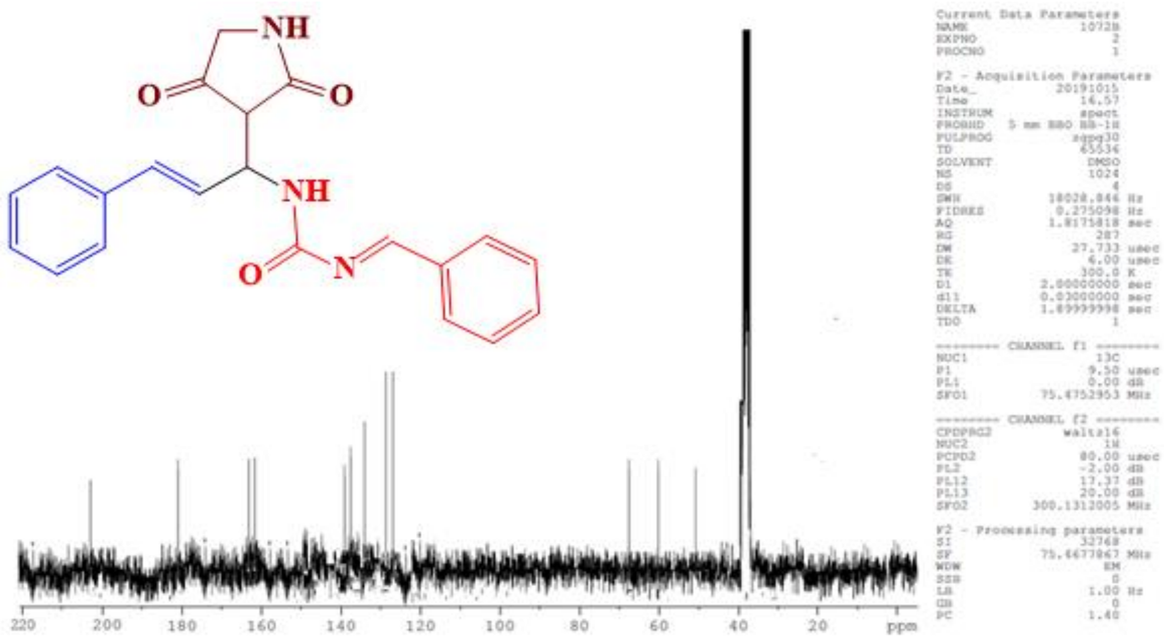

Figure S24 ^{13}C NMR spectrum of the compound **1f**

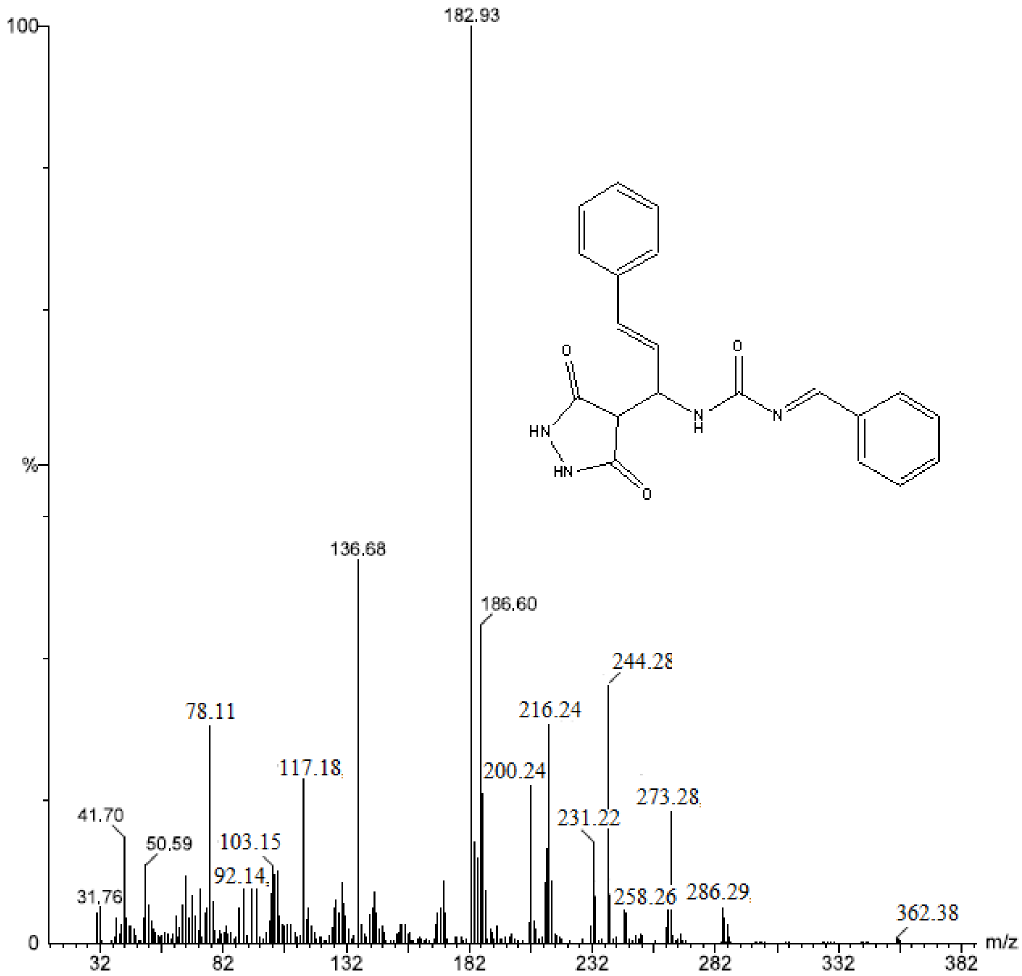

Figure S25 Mass spectrum of the compound **1f**

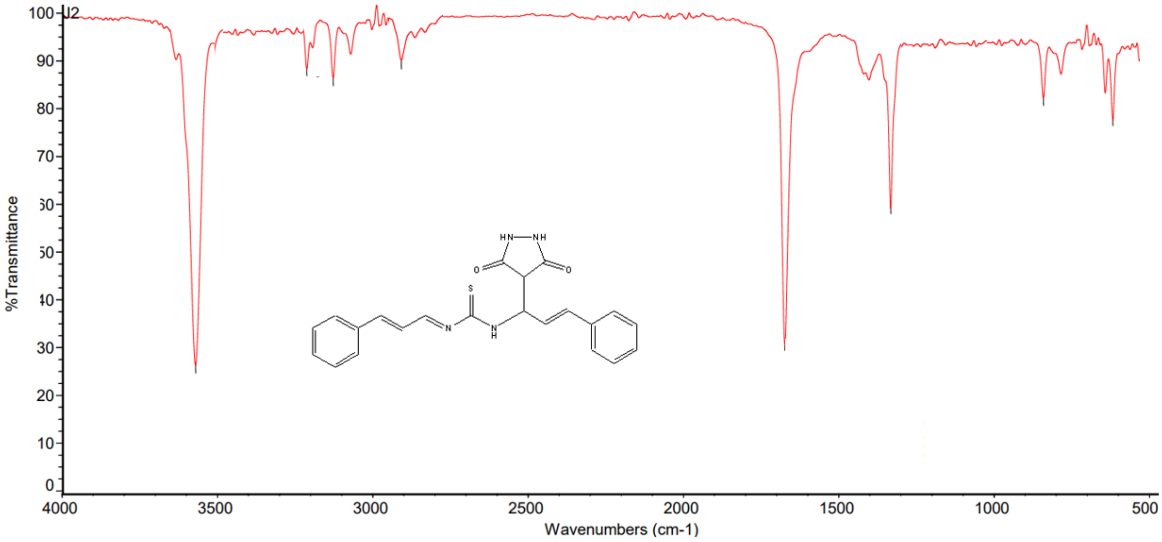

Figure S26 FTIR spectrum of the compound **1g**

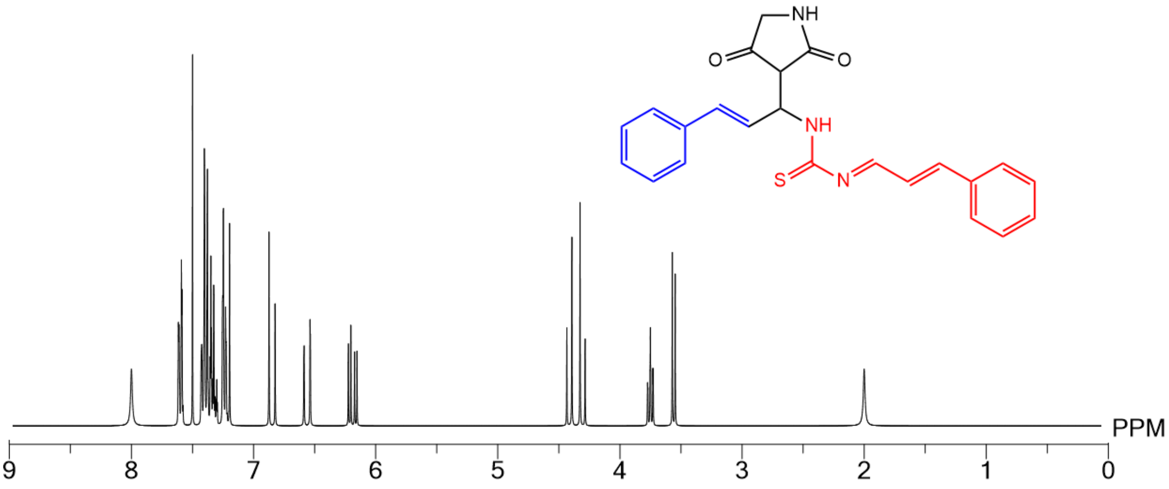

Figure S27 ^{1}H NMR spectrum of the compound **1g**

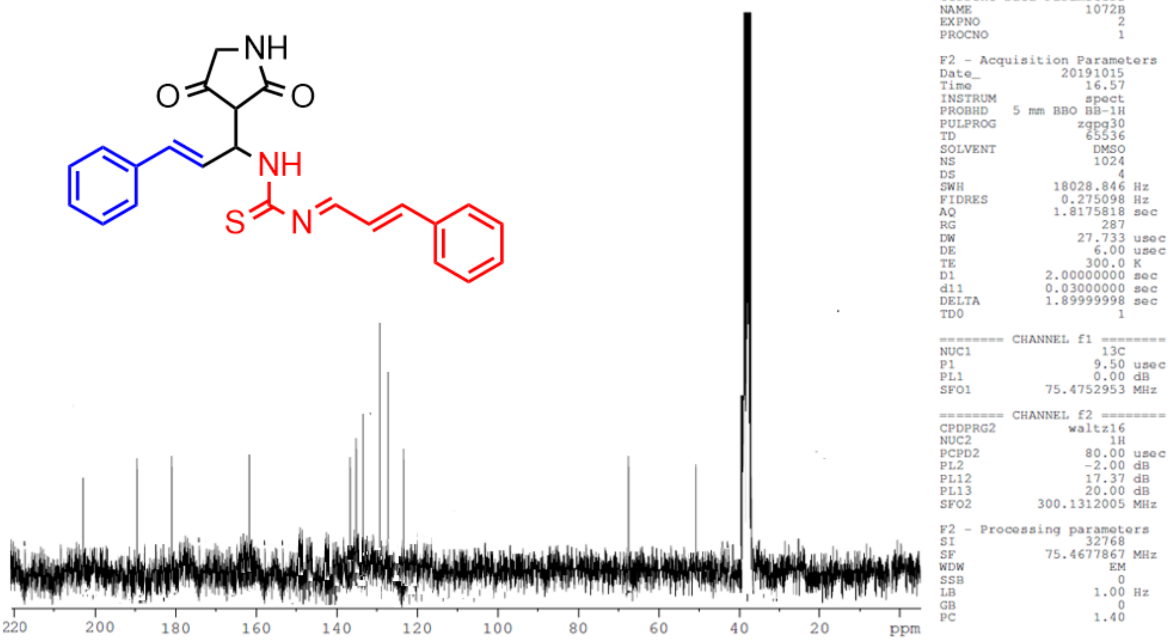

Figure S28 ^{13}C NMR spectrum of the compound **1g**

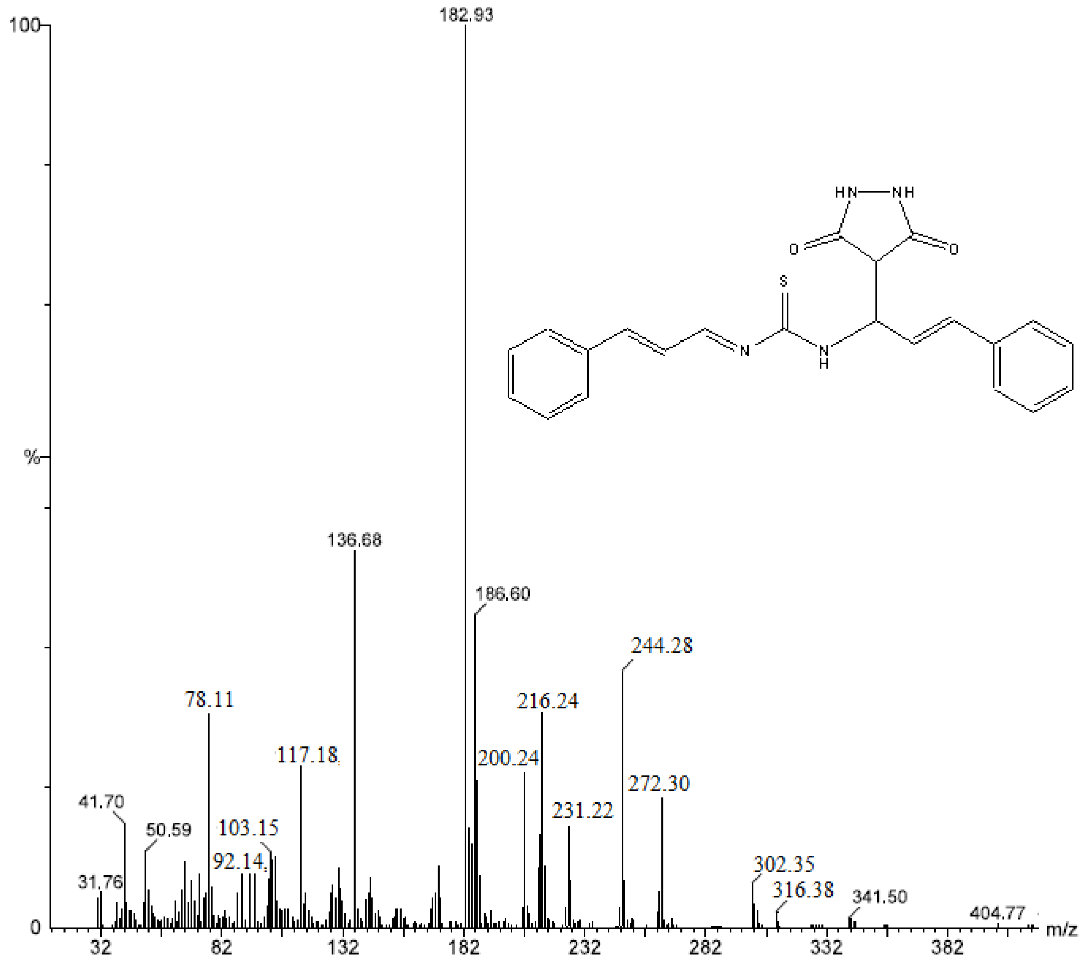

Figure S29 Mass spectrum of the compound **1g**

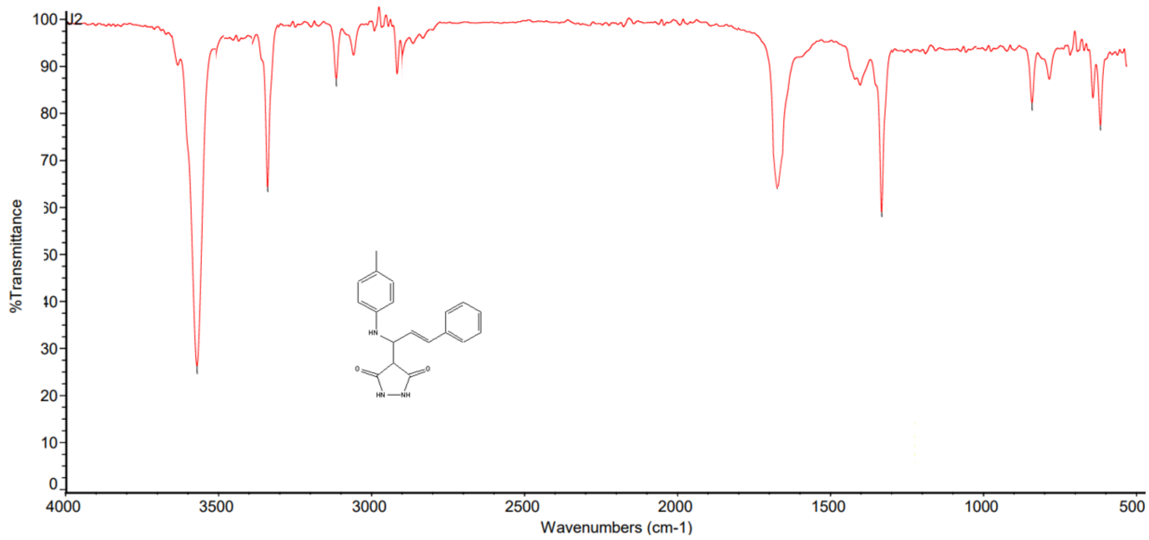

Figure S30 FTIR spectrum of the compound **1h**

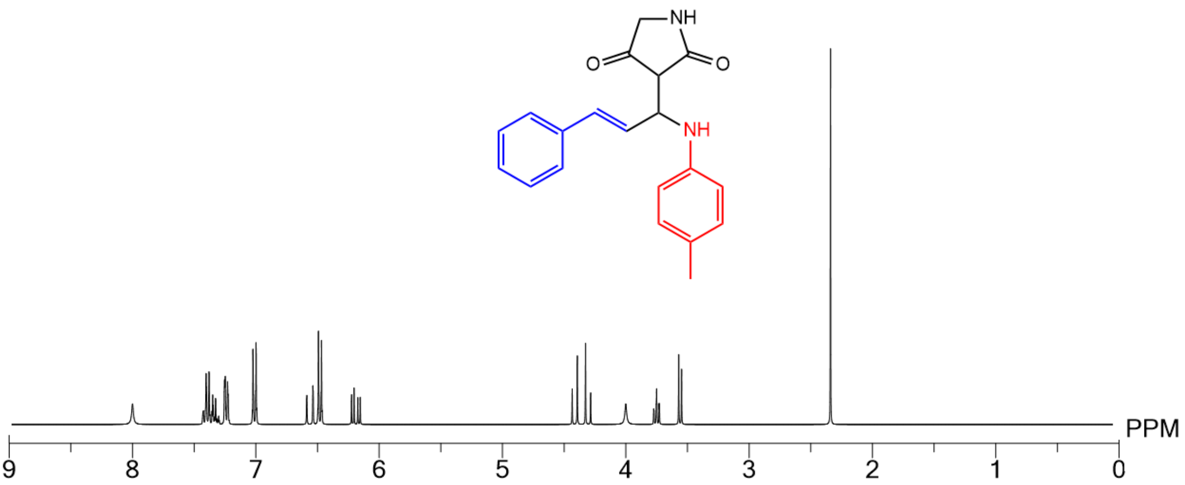

Figure S31 ^{1}H NMR spectrum of the compound **1h**

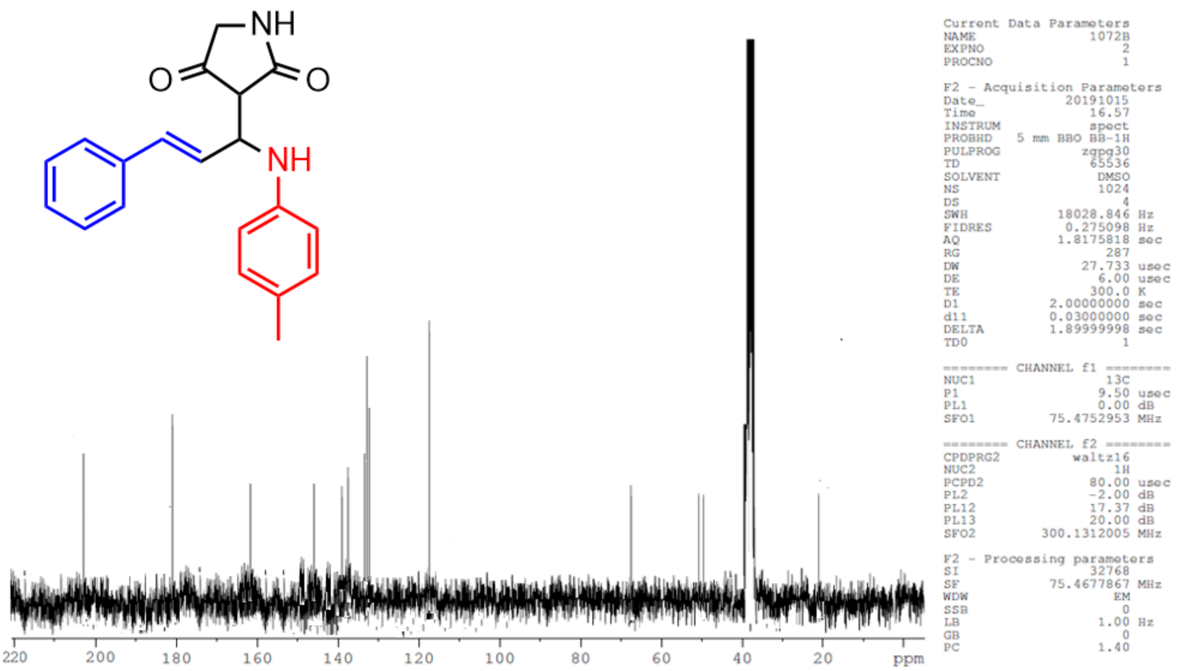

Figure S32 ^{13}C NMR spectrum of the compound **1h**

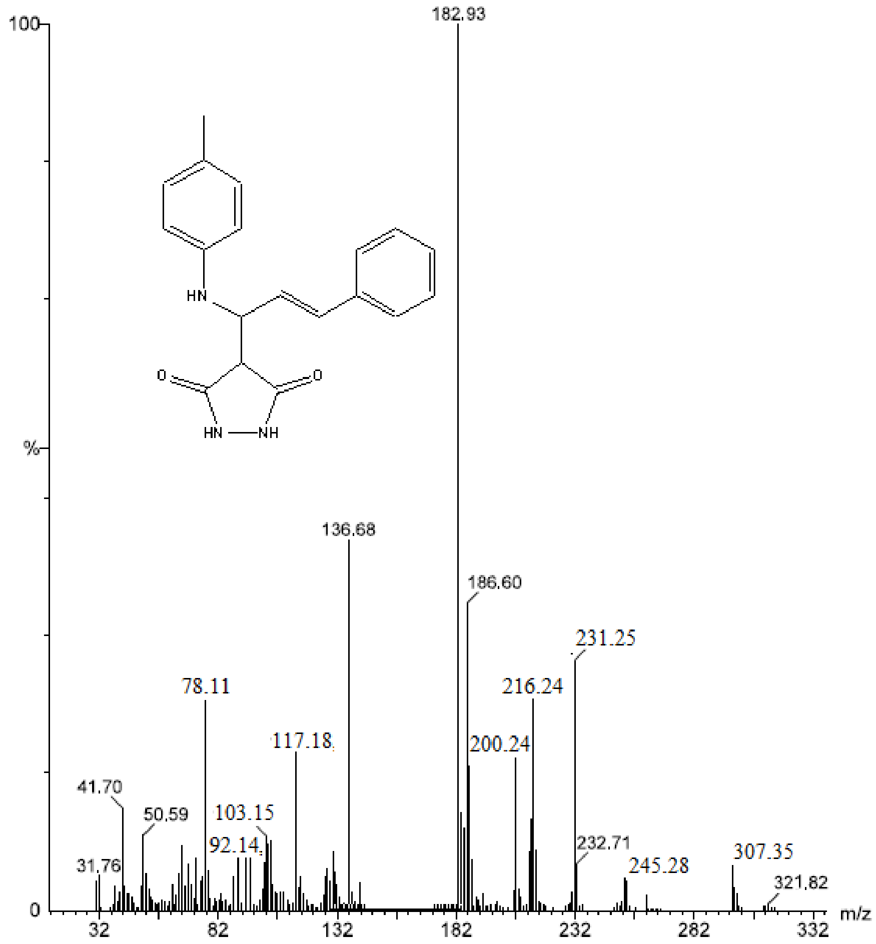

Figure S33 Mass spectrum of the compound **1h**

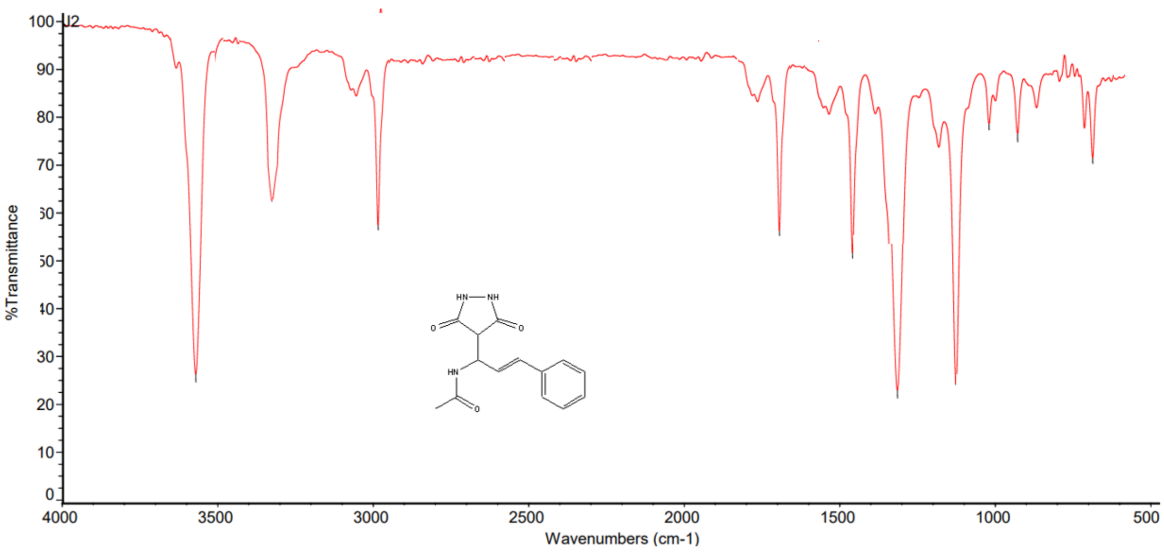

Figure S34 FTIR spectrum of the compound **1i**

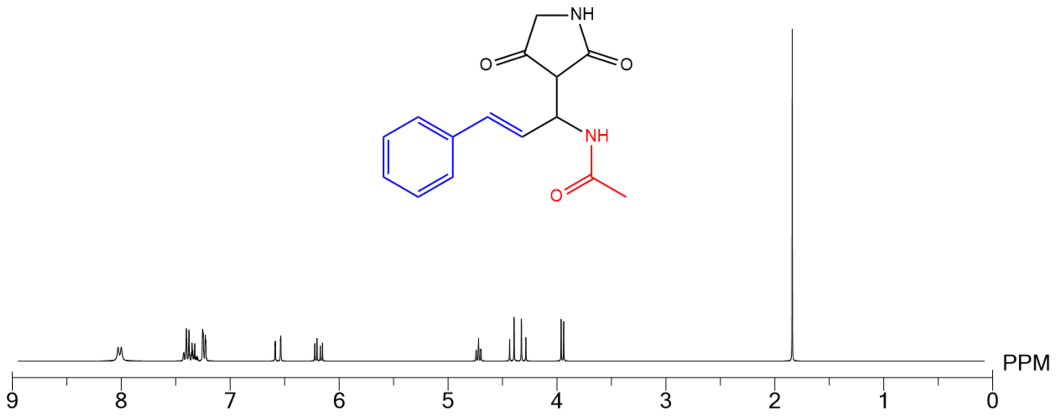

Figure S35 ^{1}H NMR spectrum of the compound **1i**

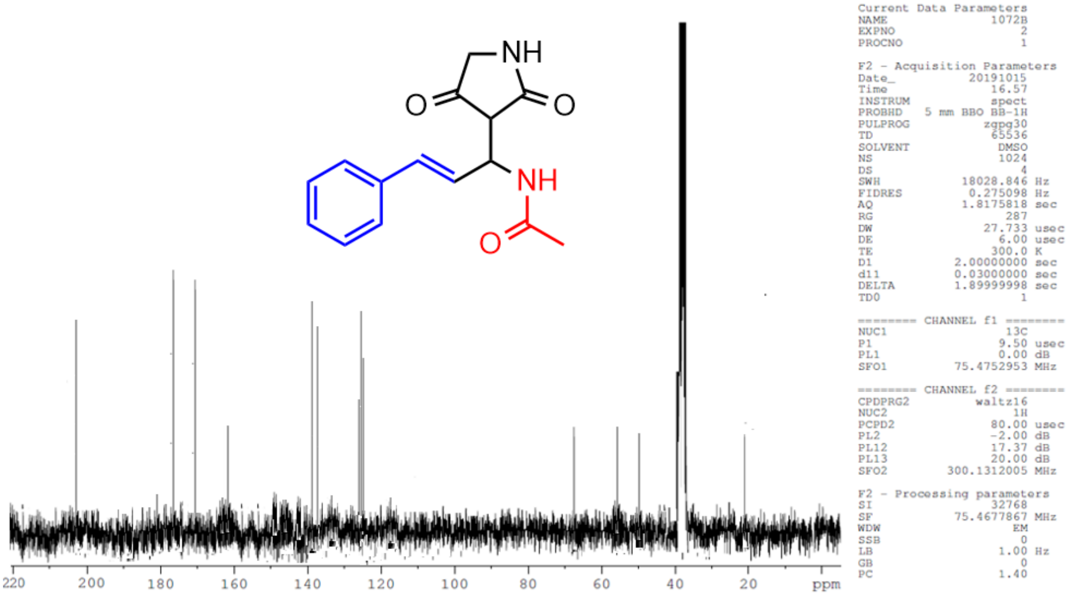

Figure S36 ^{13}C NMR spectrum of the compound **1i**

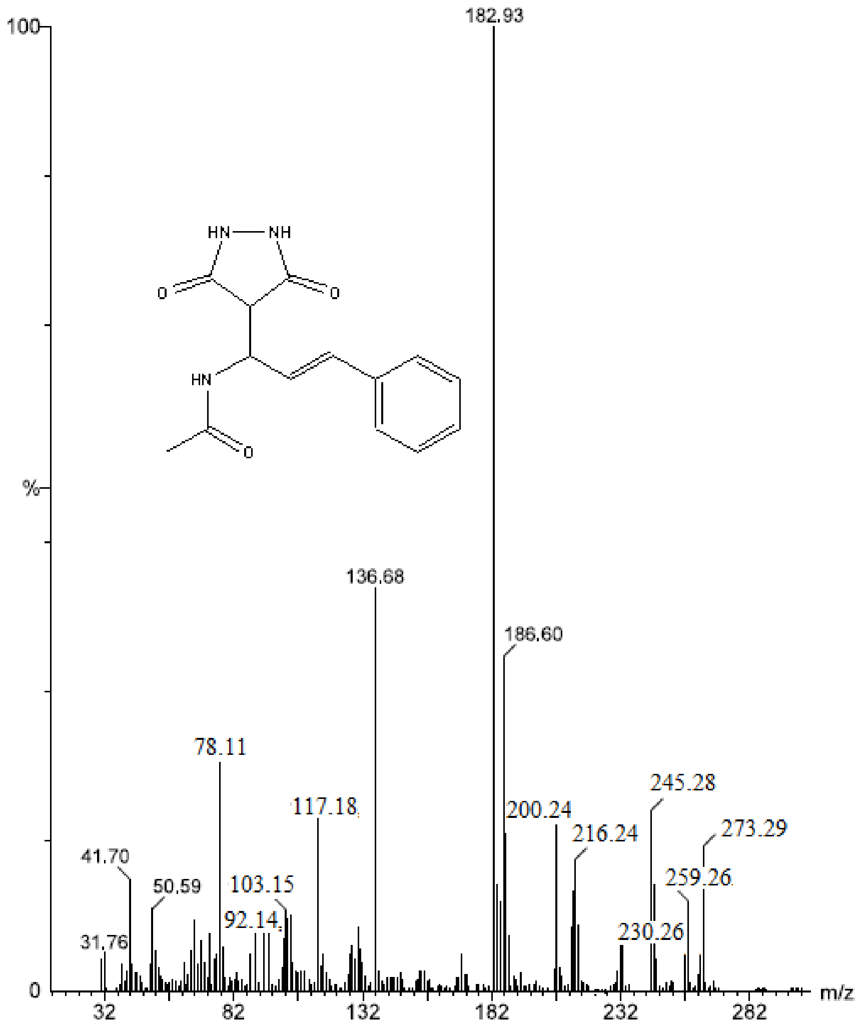

Figure S37 Mass spectrum of the compound **1i**

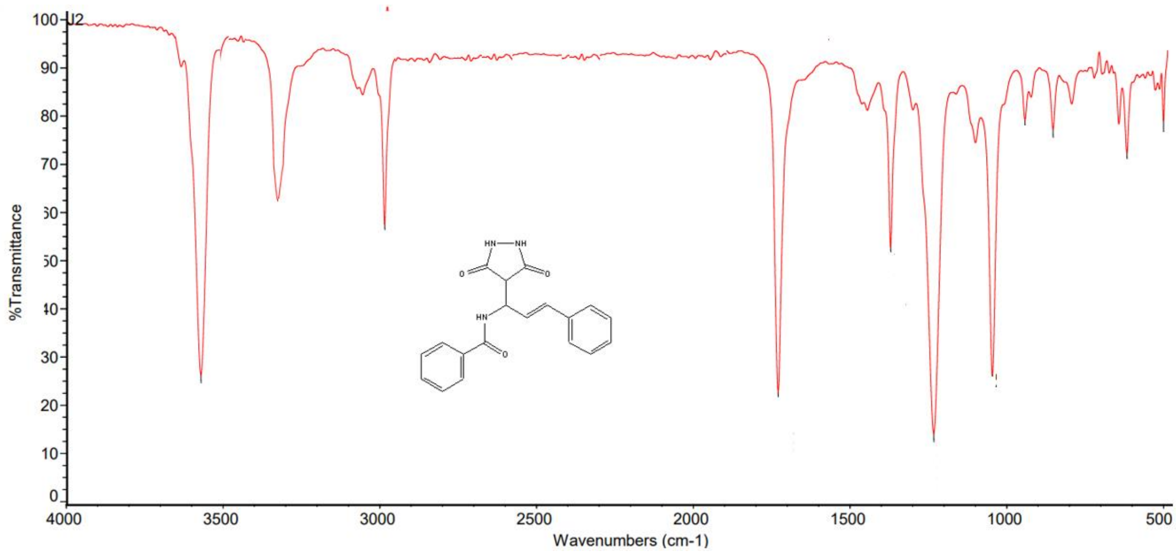

Figure S38 FTIR spectrum of the compound **1j**

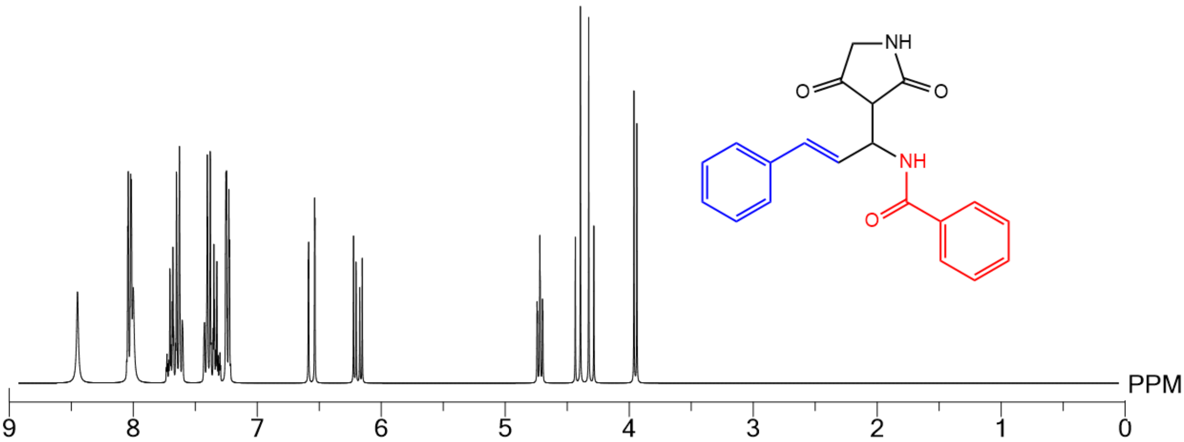

Figure S39 ^{1}H NMR spectrum of the compound **1j**

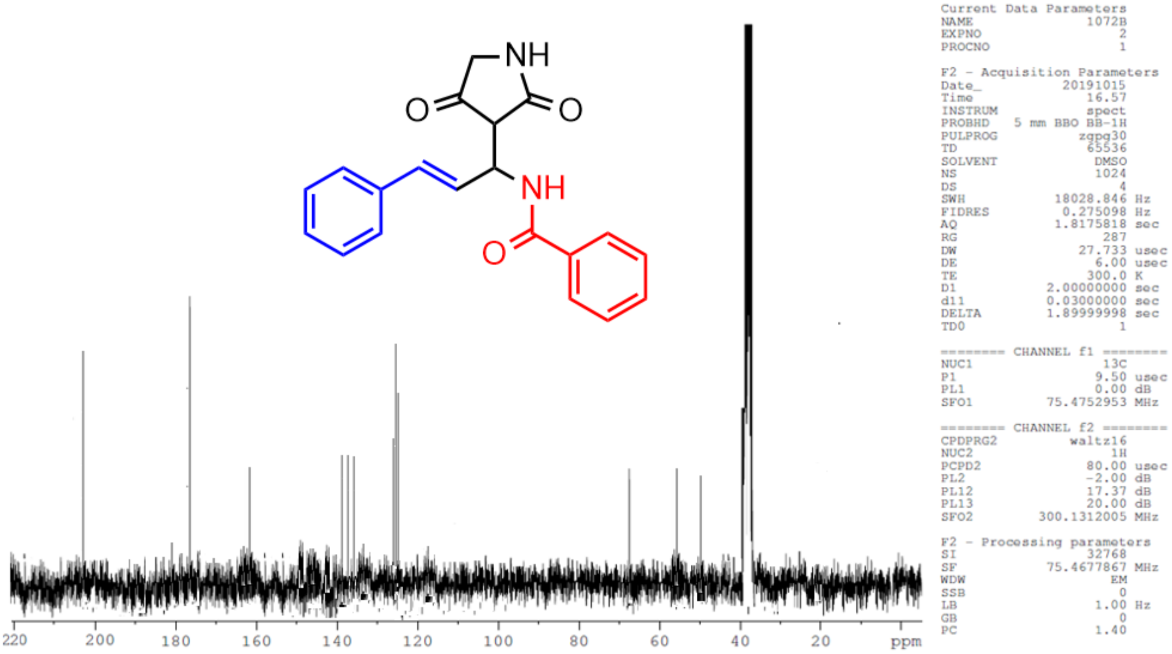

Figure S40 ^{13}C NMR spectrum of the compound **1j**

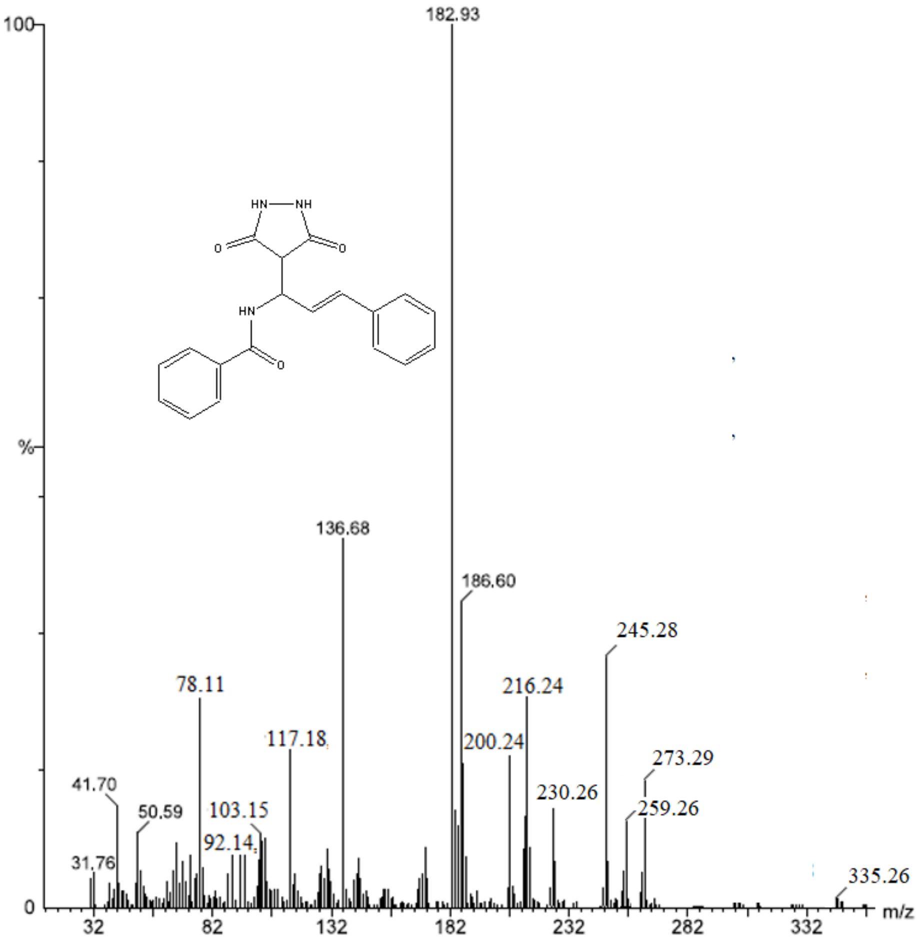

Figure S41 Mass spectrum of the compound **1j**

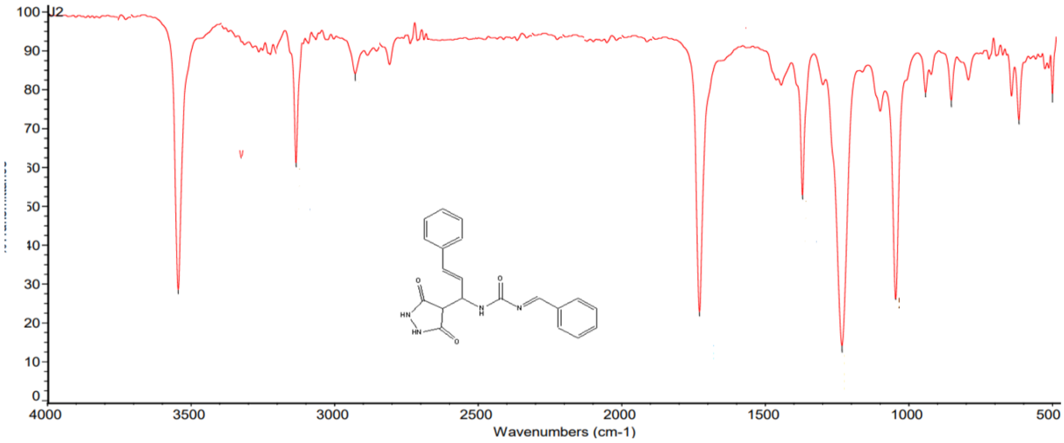

Figure S42 FTIR spectrum of the compound **1k**

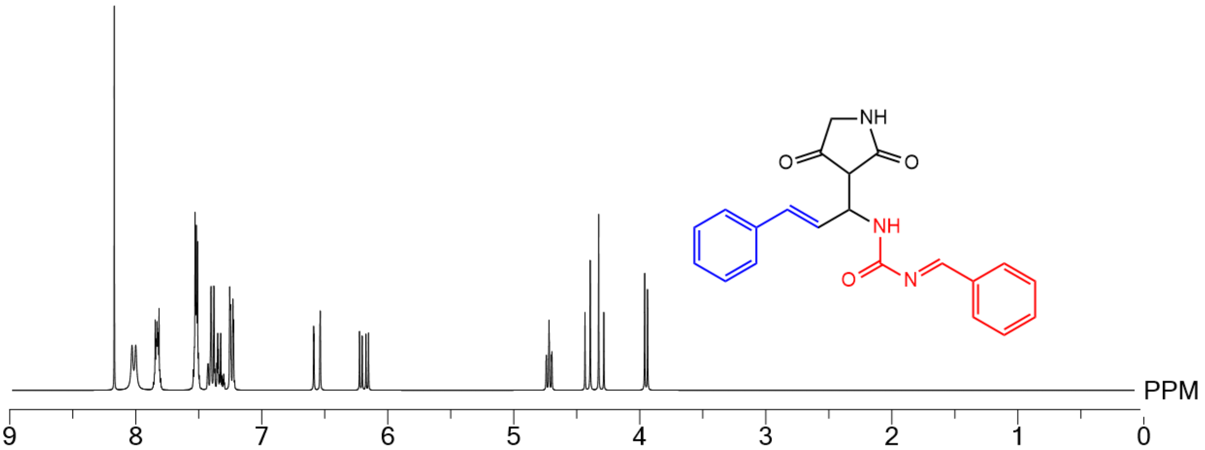

Figure S43 ^{1}H NMR spectrum of the compound **1k**

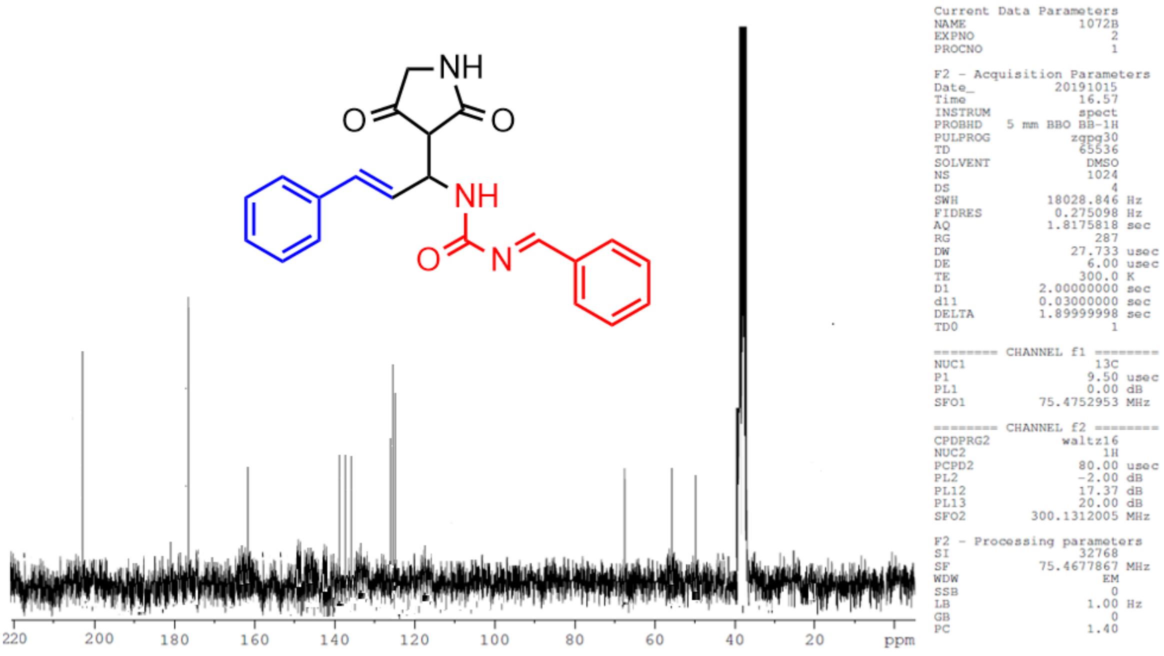

Figure S44 ^{13}C NMR spectrum of the compound **1k**

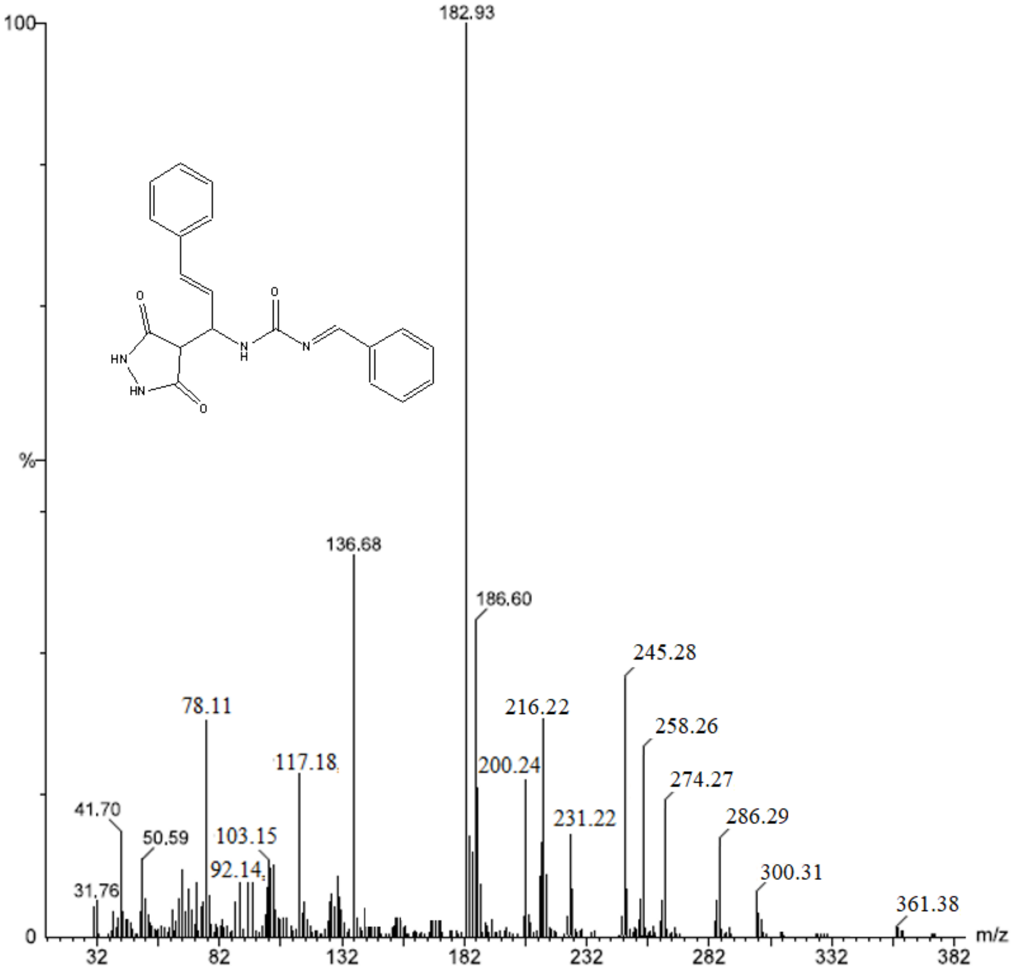

Figure S45 Mass spectrum of the compound **1k**

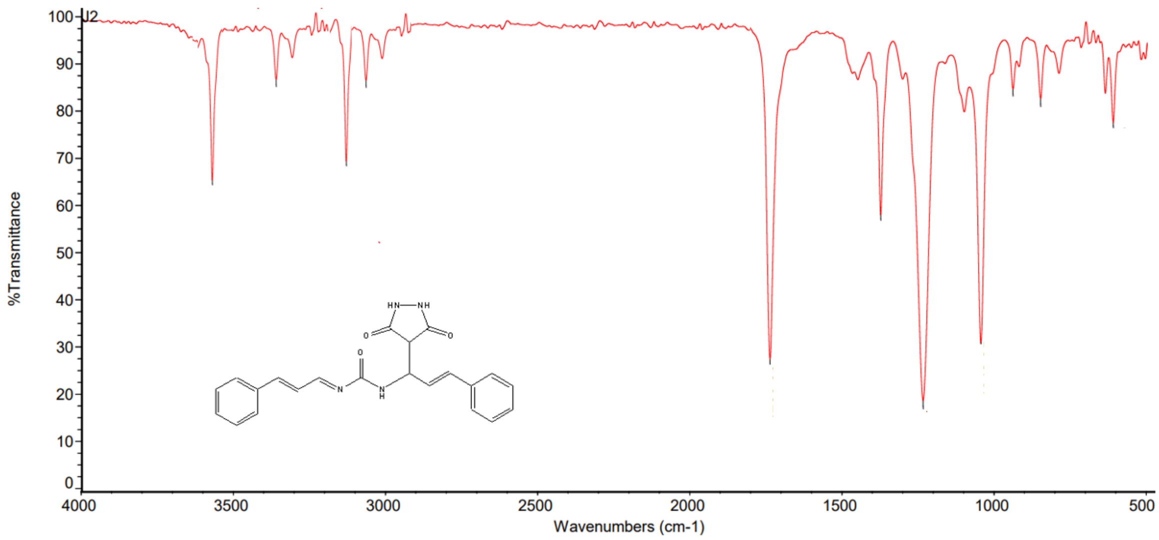

Figure S46 FTIR spectrum of the compound **1l**

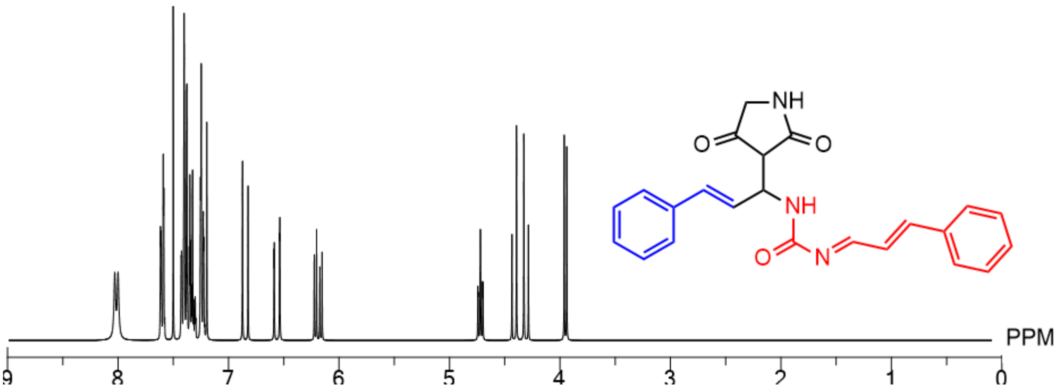

Figure S47 ^{1}H NMR spectrum of the compound **1l**

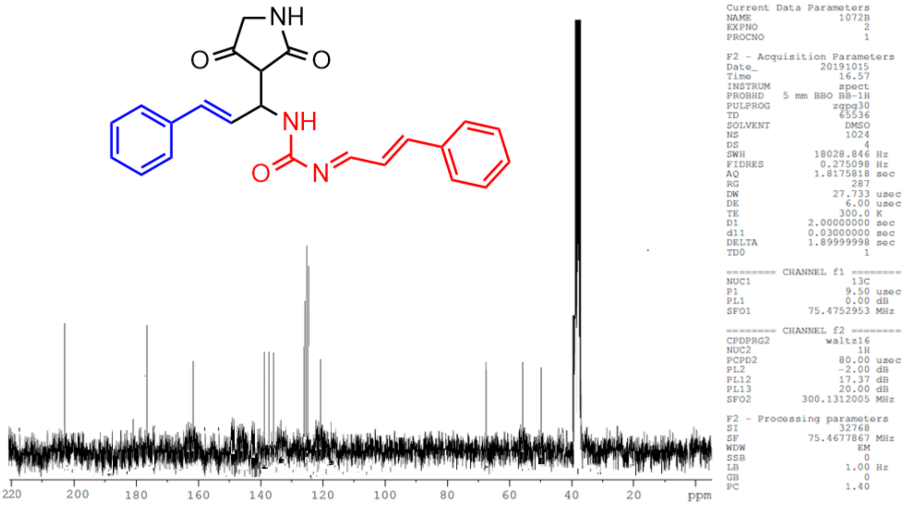

Figure S48 ^{13}C NMR spectrum of the compound **1l**

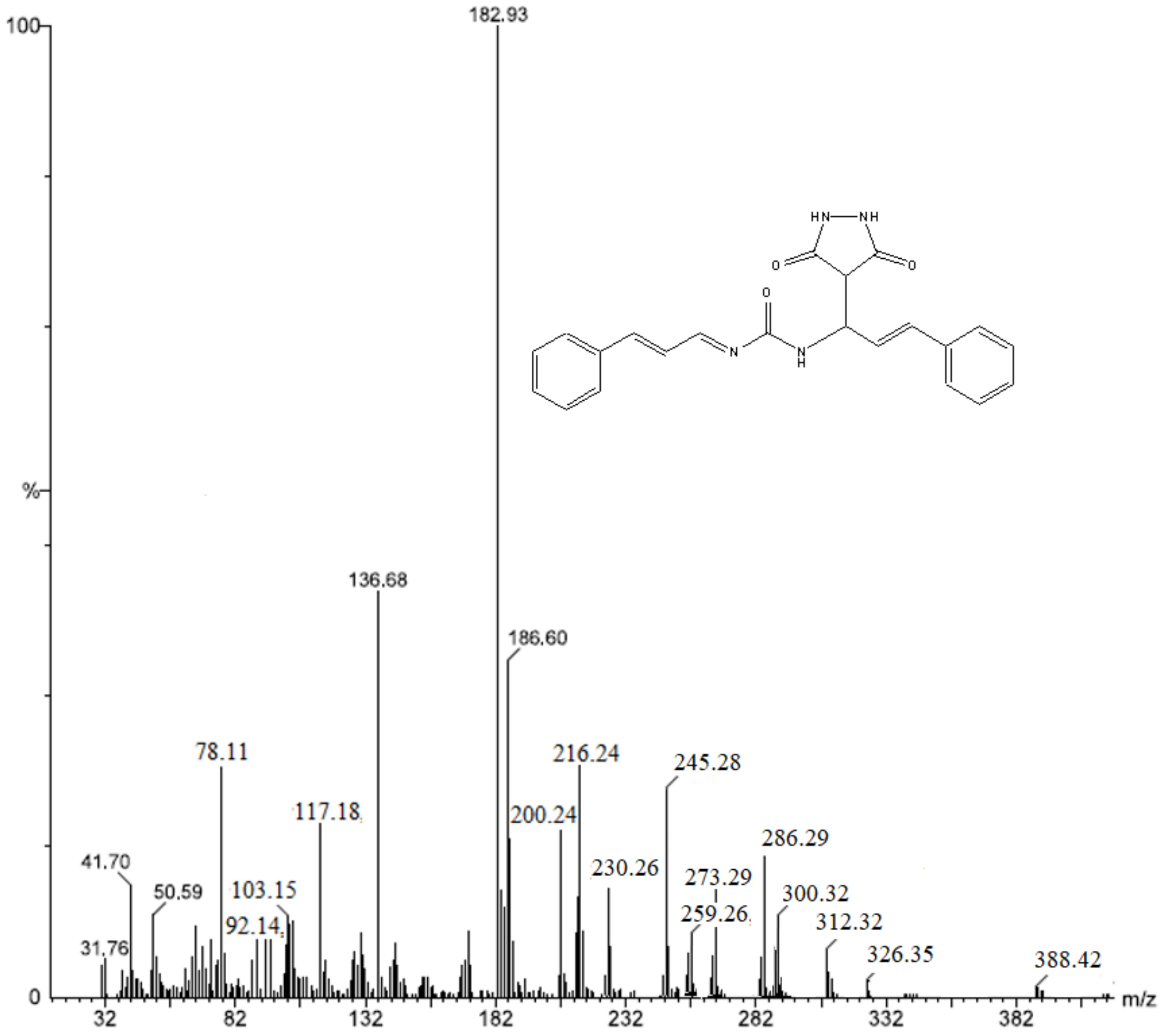

Figure S49 Mass spectrum of the compound **1l**

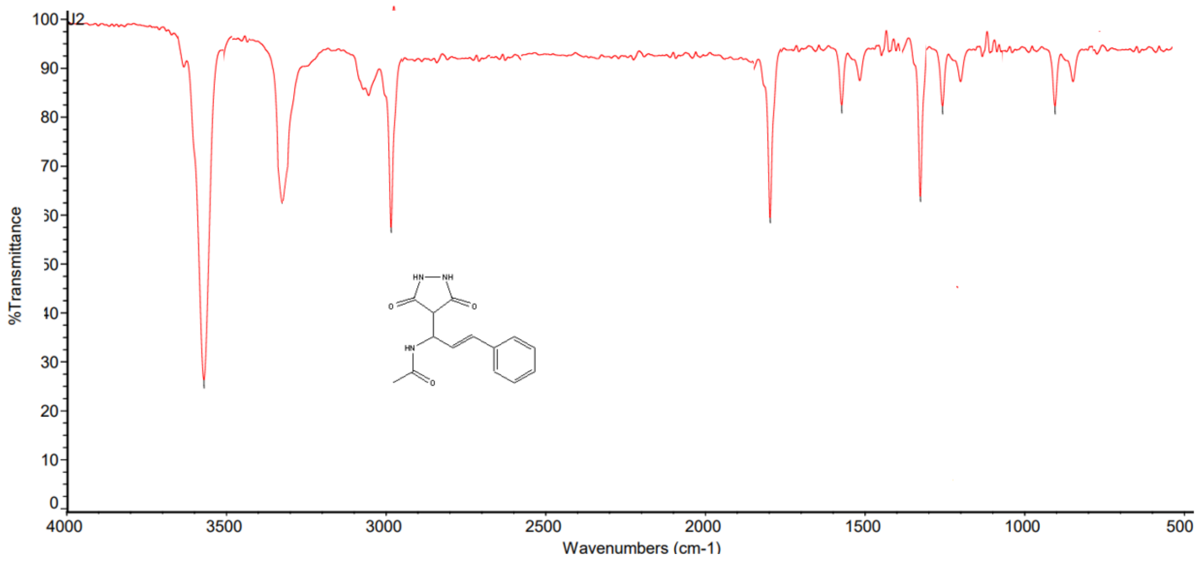

Figure S50 FTIR spectrum of the compound **1m**

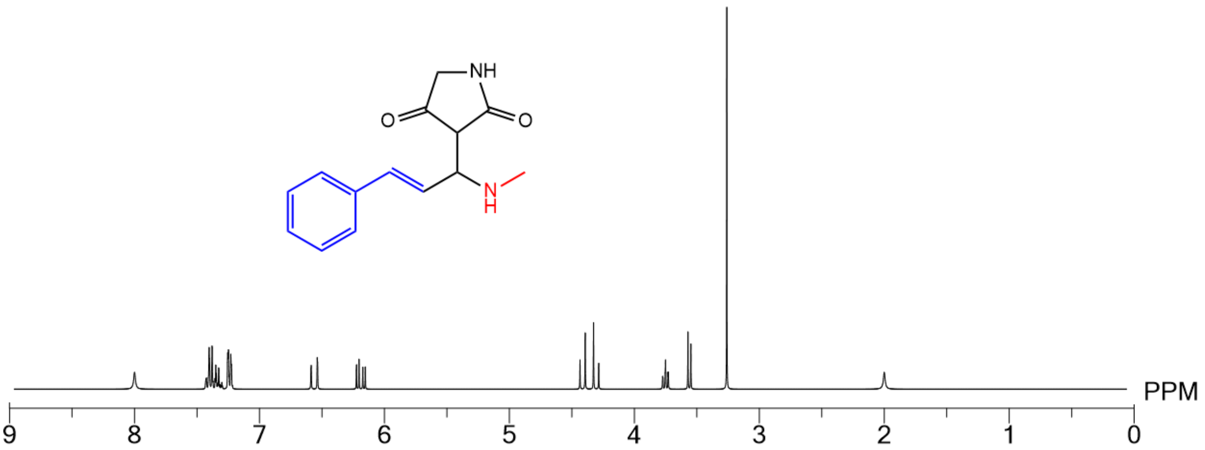

Figure S51 ^{1}H NMR spectrum of the compound **1m**

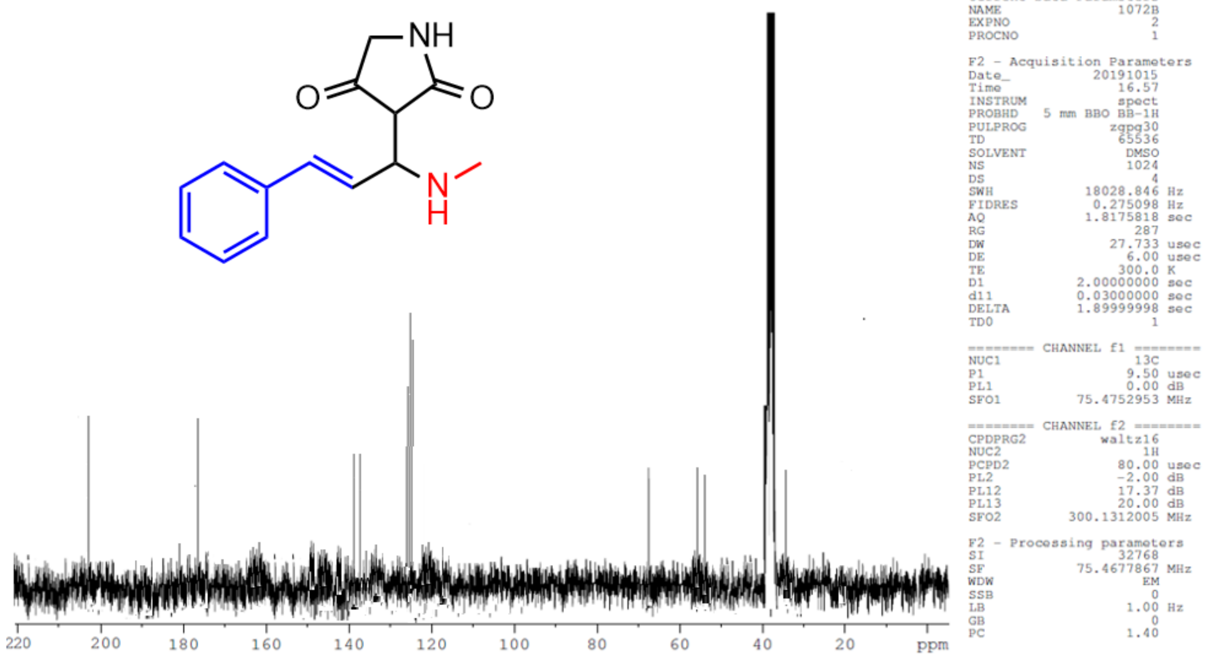

Figure S52 ^{13}C NMR spectrum of the compound **1m**

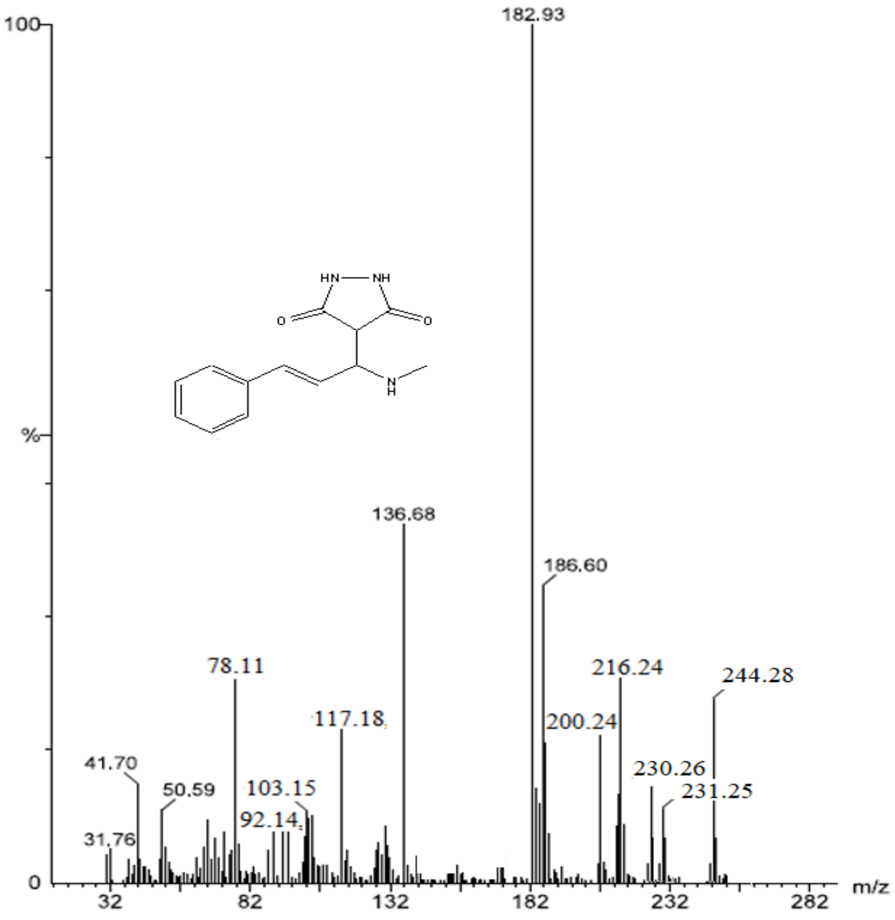

Figure S53 Mass spectrum of the compound **1m**

DFT calculation

The most significant contribution of the HOMO–LUMO analysis was from the electrical and chemical interactions of **1c**. The LUMO stands for "accept on electron," while the HOMO stands for "donate on electron." The efficiencies of the aforementioned compounds **1c** with the HOMO-LUMO value were determined by the DFT method utilising the B3LYP/6-31G (d, p) basic set. Hard compounds frequently have a larger HOMO-LUMO energy difference, whereas soft compounds have a smaller difference. The chemical stability and reactivity of the HOMO and LUMO molecules are important factors for their evaluation.

Koopman's theorem was applied to measure the HOMO and LUMO energies for 1c. The HOMO and LUMO energies were used to determine several parameters. All variables are generally related to one another and are generated from the equations given above. Because the electron affinity (**E**) and ionisation energy (**I**) of the HOMO orbital indicate that it prefers to donate electrons, whereas the LUMO orbital prefers to accept electrons, there is a strong correlation between the energies of the two orbitals. A high ΔE value indicates high stability and low chemical reactivity and serves as an indicator of chemical reactivity. Compound **1c** (ΔE gap = 0.14 eV) was chemically more stable. The global hardness (**η**), global softness (**s**), chemical potential (**μ**), and additional important factors for chemical stability, such as the **s** values for compound **1c** for 14.28 eV. The η and μ values for compound **1c** were 0.07 eV and **μ** values for compound **1c** for -0.15 eV respectively, and the **I** value for **1c** was 0.22 eV. The compounds have electronegativity (**χ**) values for 1c (χ = 0.15 eV), electrophilicity index (**ω**), and nucleophilicity (**N**) indices of **1c** (N = 6.25 eV and ω = 0.160 eV, respectively). Table S1 shows the DFT calculation properties of compound **1c.**

The B3LYP/6-31G (d, p) basis set was also used to theoretically investigate the IR spectrum. Comparison of the experimental FTIR spectra of the synthesised compounds with the simulated spectra for compound **1c**. The computed IR spectrum in animated modes was

compatible with a few significant experimental vibrational signals in the FTIR spectra of the compounds. The slight difference between the theoretical and experimental frequencies is due to the theoretical values being acquired in the gas phase and experimental values being obtained in the solid phase, and also due to neglecting the incompleteness and anharmonicity of the basis set. Therefore, to reduce these differences, a scaling factor was used.

Table S1 DFT calculation properties of compound **1c**

S. No	Property	1C
1	Homo	-0.22
2	Lumo	-0.08
3	Energy gap ΔE (LUMO-HOMO)	0.14
4	Ionization Energy (I=εHOMO=-HOMO)	0.22
5	Electron Affinity(A=εLUMO=-LUMO)	0.08
6	Global Hardness (η= (I-A)/2	0.07
7	Global Softness (s=1/η)	14.28
8	Chemical Potential (μ=-(I+A)/2	-0.15
9	Electronegative (χ = -μ)	0.15
10	Electrophilicity Index (ω= μ2/2η)	0.160
11	Nucleophilicity Index (N=1/ω)	6.25
